# Opto-electronic feedback control of membrane potential for real-time control of action potentials

**DOI:** 10.1016/j.crmeth.2023.100671

**Published:** 2023-12-11

**Authors:** Balázs Ördög, Tim De Coster, Sven O. Dekker, Cindy I. Bart, Juan Zhang, Gerard J.J. Boink, Wilhelmina H. Bax, Shanliang Deng, Bram L. den Ouden, Antoine A.F. de Vries, Daniël A. Pijnappels

**Affiliations:** 1 Laboratory of Experimental Cardiology, Department of Cardiology, Heart Lung Center Leiden, Leiden University Medical Center, 2333 ZA Leiden, the Netherlands; 2 Amsterdam Cardiovascular Sciences, Department of Cardiology, Amsterdam UMC, Location AMC, University of Amsterdam, 1105 AZ Amsterdam, the Netherlands; 3 Department of Medical Biology, Amsterdam UMC, Location AMC, University of Amsterdam, 1105 AZ Amsterdam, the Netherlands; 4 Department of Microelectronics, Delft University of Technology, 2628 CD Delft, the Netherlands

**Keywords:** action potential, membrane potential, cellular electrophysiology, feedback loop, real-time computing, optogenetics, closed-loop control, system integration, heart muscle cells

## Abstract

To unlock new research possibilities by acquiring control of action potential (AP) morphologies in excitable cells, we developed an opto-electronic feedback loop-based system integrating cellular electrophysiology, real-time computing, and optogenetic approaches and applied it to monolayers of heart muscle cells. This allowed accurate restoration and preservation of cardiac AP morphologies in the presence of electrical perturbations of different origin in an unsupervised, self-regulatory manner, without any prior knowledge of the disturbance. Moreover, arbitrary AP waveforms could be enforced onto these cells. Collectively, these results set the stage for the refinement and application of opto-electronic control systems to enable in-depth investigation into the regulatory role of membrane potential in health and disease.

## Highlights

• APqr integrates cellular electrophysiology, real-time computing, and optogenetics
• APqr preserves cardiac AP morphologies in the presence of electrical perturbations
• APqr allows the enforcement of arbitrary AP waveforms
• APqr sets the stage for tissue-scale membrane potential control

## Motivation

In excitable cells, the membrane potential undergoes transient, periodic changes called action potentials (APs). Organ-level function, such as heart contraction, depends on distinct local AP morphologies, whereas AP abnormalities can give rise to disorders, such as cardiac arrhythmias. APs are highly dynamic, multifactorial processes and have stochastic characteristics. To gain a deeper understanding into the regulatory roles of APs in health and in the development and/or progression of disease, experimental tools that are able to control APs in a precise and similarly dynamic manner are needed. Yet, methods capable of such control are currently lacking.

## Graphical abstract

**Figure:**
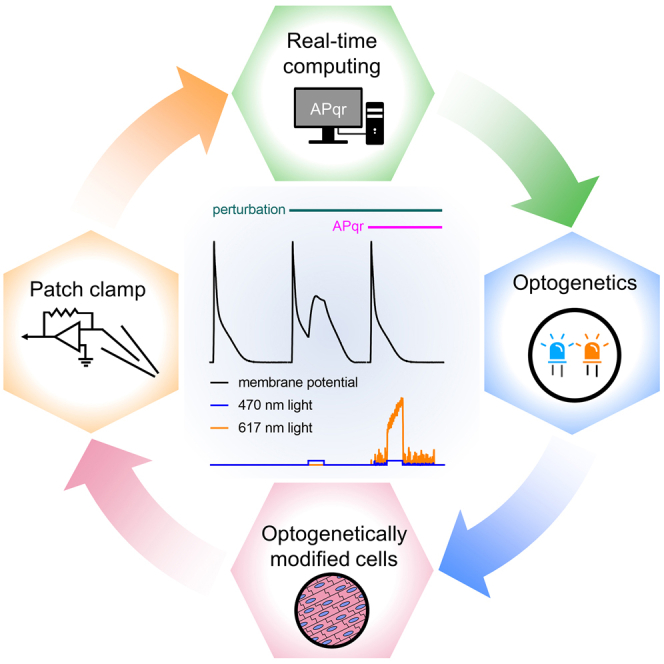

## Teaser

Ördög et al. establish APqr, a closed-loop experimental system for the dynamic control of the membrane potential in cardiomyocytes. Their approach sets the stage for the refinement and application of opto-electronic control systems to enable in-depth investigation into regulatory roles of the membrane potential in health and disease.

## Introduction

All living cells maintain a membrane potential (V_m_). V_m_ is determined by the distribution of ions between the extra- and intracellular space established by membrane-resident ion channels, exchangers, and pumps.^1^ In excitable cells, V_m_ undergoes fast and transient changes called action potentials (APs), which may propagate unidirectionally within the cell membrane and between electrically coupled cells. In the nervous system and the heart, organ-level functions depend on finely tuned and often distinctly different local AP morphologies.^2^^,^^3^ Furthermore, subtle abnormalities of local AP properties may lead to organ dysfunction.^4^ However, due to the limitations of methods available for V_m_ control and in particular for the control of AP morphologies, the exact roles of AP characteristics in health and disease and the potential benefits of AP abnormality correction remain incompletely understood.

V_m_ can be controlled by the so-called voltage clamp modality of the patch-clamp technique, where V_m_ is clamped to predetermined values, and the concurrent transmembrane currents are measured. Interestingly, V_m_ can also be clamped to a series of voltages that collectively give rise to the shape of an AP. These voltages can either be set prior to the experiment in the so-called AP clamp configuration or simulated real-time in dynamic AP clamp experiments.^5^^,^^6^^,^^7^^,^^8^^,^^9^ A common feature of all types of voltage clamp, however, is that the desired V_m_ value is imposed on the cell by direct current injection via the recording electrode. This limits the applicability of voltage clamp in the multicellular setting, especially in tissue with low net impedance, such as cardiac muscle tissue.

The transmembrane currents needed to control V_m_, however, may be generated independently of the tissue`s impedance by means of optogenetics. Optogenetics is a highly versatile and rapidly evolving research methodology that employs light-sensitive proteins to observe and/or interfere with cell function. For example, exploiting their ability to depolarize the cell membrane upon illumination, light-gated cation channels known as channelrhodopsins (ChRs)^10^^,^^11^ have been used in a wide range of applications, including cardiac pacing,^12^^,^^13^ resynchronization of heart segments,^14^ termination of cardiac arrhythmias,^15^^,^^16^^,^^17^ and for the optical manipulation of excitation waves both in monolayers of cardiomyocytes^18^^,^^19^ and in the intact heart.^20^ Furthermore, optogenetics has been used as a direct, non-invasive replacement of current injection for different purposes. Bidirectional V_m_ control was achieved by the selective activation of ChR2 and an outward-current-generating opsin (i.e., ArchT or eNpHR3.0) in order to clamp the activity of neurons to specific firing rates^21^ and to create the voltage steps necessary to activate ectopically expressed ion channels for drug profiling purposes.^22^ ArchT has also been used for the supplementation of induced pluripotent stem cell-derived cardiomyocytes (iPSC-CMs) with the I_K1_ current in an optical dynamic clamp arrangement, thereby improving AP characteristics of these cells.^23^ More recently, an all-optical voltage clamp platform was applied to control neural activity by the combined use of BIPOLES, a fusion between a cation- and an anion-selective ChR shunting V_m_ to specific voltages in a wavelength-dependent manner, and of a fluorescent V_m_ readout in a closed-loop arrangement.^24^

Previous studies already demonstrated the ability to optogenetically modulate AP waveforms. A computational study explored the feasibility of optogenetic prolongation of action potential durations (APDs) in a three-dimensional model of human atria with pathological APD shortening.^25^ In the experimental setting, Park et al. showed light-induced prolongation and shortening of APD in neonatal rat ventricular myocytes via activation of ChR2 and eNpHR3.0, respectively.^26^ Using the same cell type, Govorunova et al. achieved APD shortening in a light duration- and intensity-dependent manner by activation of the anion ChR GtACR1.^27^ Furthermore, Gruber et al. optogenetically corrected abnormal APDs in patient-derived iPSC-CMs in the context of long and short QT syndromes.^28^ In all former studies aiming at the control and/or correction of AP waveforms, the characteristics of the modulatory light pulses (e.g., timing, intensity, and duration) and of the endogenous APs needed to be determined before the intervention. While such deterministic approach is robust and effective, it needs to be tailored to every combination of cell type and pathology (e.g., short or long QT syndrome-causing mutations).

**Figure 1:**
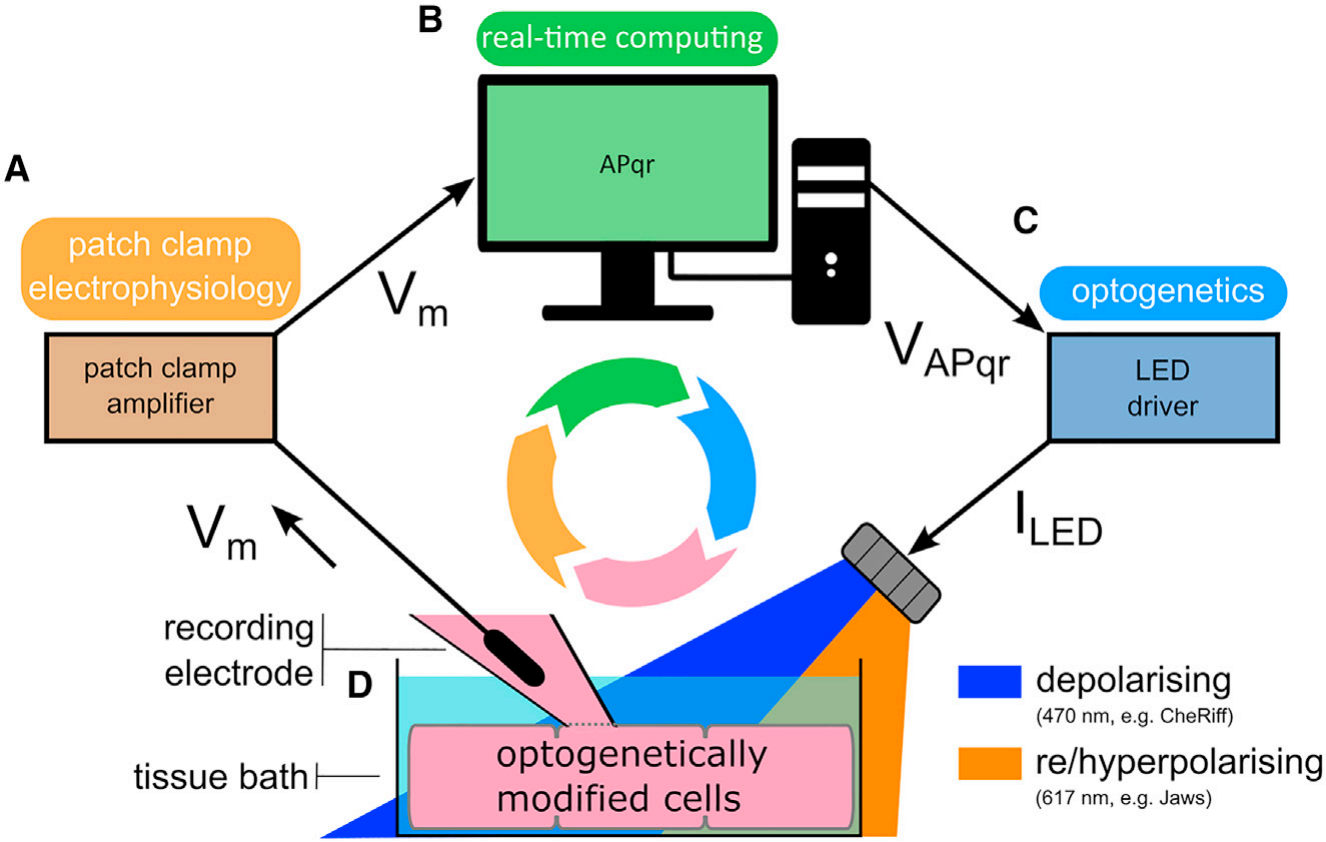
Schematic illustration of the main components of the experimental system used in this study (A) Real-time, accurate V_m_ measurement is obtained by patch-clamp electrophysiology in hiAMs in single-cell or monolayer formats. (B) V_m_ data fed to the custom closed-loop control algorithm (APqr) hosted on a real-time computing platform generating the dynamic output V_APqr_. (C and D) Optogenetic approaches are used to interface real-time computing and the biological component, consisting of LED light sources controlled by V_APqr_ (C) and optogenetic actuators expressed in hiAMs (D). See also Figure S1A.

Here, we now show that this customization is no longer necessary by employing closed-loop control principles, an inherently adaptive systems’ control scheme widely used in physiological regulatory mechanisms.^29^ Our system, herein referred to as APqr, uses real-time V_m_ data obtained by patch-clamp electrophysiology (Figure 1A) as the only input without any prior calibration. V_m_ data are fed to a custom feedback loop controller algorithm hosted on a real-time computing platform creating the dynamic output V_APqr_ (Figure 1B). Finally, optogenetics is used to interface real-time computing and the biological components (Figure 1C), as explained in detail in the STAR Methods and the following sections. The blue light-gated cation channel CheRiff^30^ and the red light-activatable inward chloride pump Jaws^31^ are used as effectors for direct targeting of V_m_ in conditionally immortalized human atrial myocytes (hiAMs, Figure 1D).^32^ By using APqr, AP morphologies perturbed either by light or by drug exposure can be restored to their normal states in a dynamic, self-regulatory manner in multicellular hiAM clusters without any prior information on the disturbance. In addition, arbitrarily chosen AP waveforms can be enforced onto cardiomyocytes with physiologically relevant accuracy and precision.

## Results

### AP shape restoration

Since abnormal AP waveforms can have detrimental consequences, their restoration to a normal state may be beneficial. To achieve this, APqr was designed to accomplish the following functionalities. When activated, APqr first logs a chosen number of APs, averages them across time, and stores the average AP as V_m_ setpoint values. Following the logging phase, APqr calculates the difference between the measured and setpoint V_m_ value as V_m_ error and generates an output (V_APqr_, Figures 1 and S1) seeking to minimize V_m_ error. Since V_APqr_ is proportional to V_m_ error and is adjusted after evaluating a set of conditions (e.g., overshoot or increasing V_m_ error), the control feedback loop is fully adaptive. These calculations are carried out at fixed time steps: once per millisecond in all our experiments. AP abnormalities were induced either by means of a preprogrammed blue light pulse activating CheRiff or by non-selective potassium channel blockade (4-aminopyridine [4AP], 200 μM). In experiments with 4AP, APqr was paused after the logging phase to allow 4AP effects to develop. When 4AP effects became evident, APqr was resumed and left running without any further input from the experimenter.

#### AP shape restoration by direct current injection in single cells

**Figure 2:**
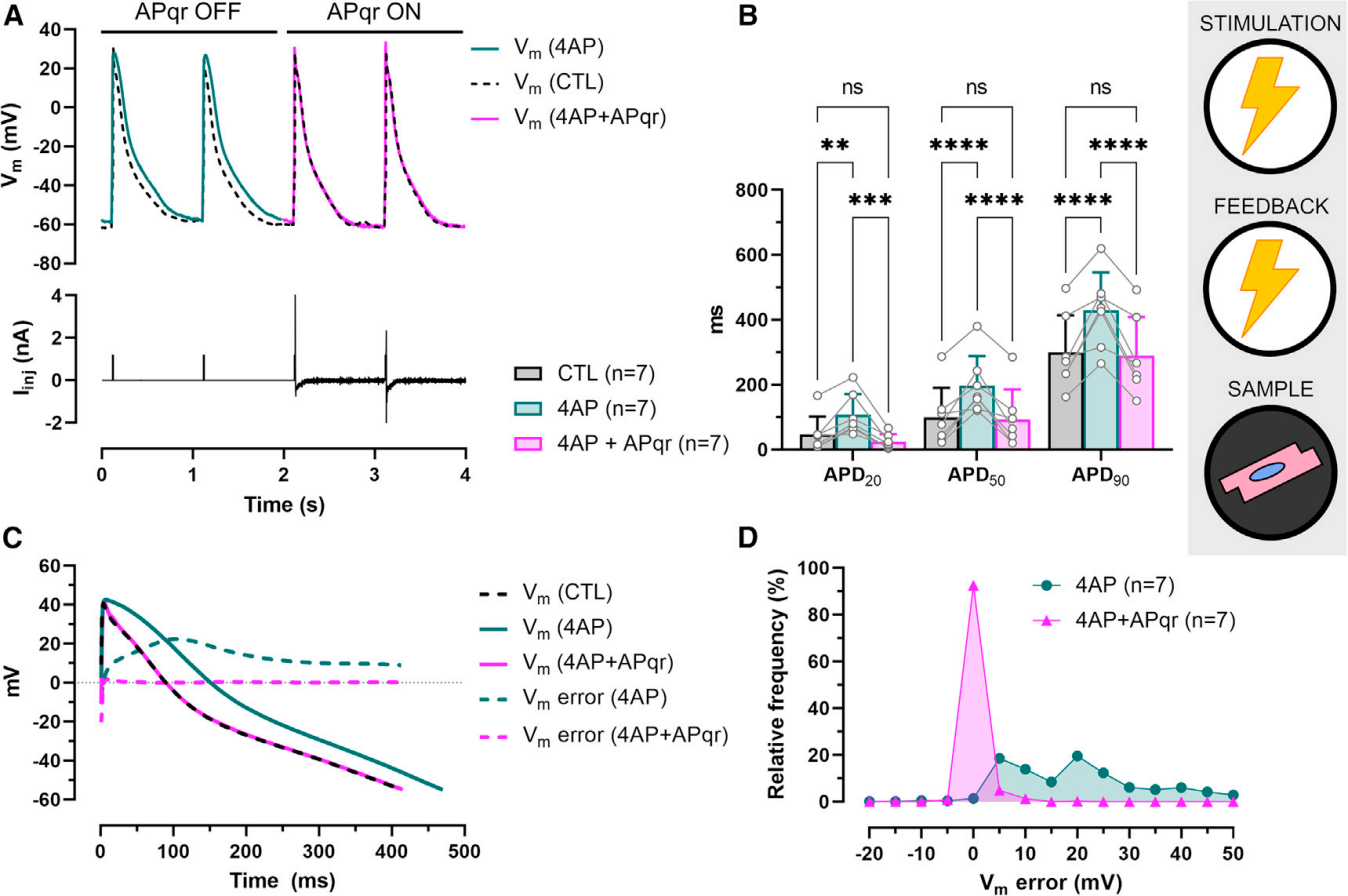
AP shape restoration by direct current injection in single cells (A) Representative recordings of electrically triggered APs (upper panel) under normal conditions (CTL), following 4AP administration (4AP), and during APqr intervention in the presence of 4AP (4AP + APqr), and of the injected current (lower panel) in a single hiAM. (B) Mean APD values ±SD measured at 20%, 50%, and 90% repolarization in n = 7 single hiAMs. ∗∗p < 0.005, ∗∗∗p < 0.001, ∗∗∗∗p < 0.0001, ns: not significant. (C) Representative recordings of APs shown between the moment of dV/dt_max_ and the moment of APD_90_, overlaid with V_m_ error calculated for the drug-affected (4AP) and restored (4AP + APqr) APs compared to CTL. (D) Frequency distribution of V_m_ error in n = 7 single hiAMs before (4AP) and during AP shape restoration (4AP + APqr) with a 5-mV bin width. See also Figure S2A.

APqr was first used to target V_m_ by direct current injection via the recording patch-clamp electrode in single hiAMs. In this arrangement, V_APqr_ served as control signal for the patch-clamp amplifier (V_com_) producing the injected current (I_inj_) proportional to V_APqr_ (Figure S1A). Electrical perturbance was created by 4AP administration. 4AP is expected to slow down the repolarization phase of the AP (return from AP peak to resting potential). In accordance with this, APs indeed showed a broadened morphology in the presence of 4AP when APqr was paused (APqr OFF, Figure 2A). However, after activation of APqr, normal AP morphologies were maintained (APqr ON, Figure 2A). The effects of 4AP and the APqr intervention were reflected in APD values calculated as the interval between the moment of maximum upstroke velocity (dV/dt_max_) and the moment at which various (20%, 50%, and 90%) repolarization levels were reached (Figure 2B). Administration of 4AP prolonged the APD as reflected by significantly increased average APD_20_, APD_50_, and APD_90_ values compared to control conditions (CTL, n = 7, p < 0.005). With APqr ON (4AP + APqr), however, average APDs were similar to CTL (p > 0.05, Figure 2B). For example, while APD_90_ values increased from 299.3 ± 115.3 ms in CTL to 429.9 ± 115.6 ms with 4AP, APqr reduced average APD_90_ to 289.8 ± 118.5 ms in the 4AP + APqr group, thereby reducing the 140.1-ms 4AP-induced APD_90_ difference to 9.5 ms on average. The efficacy of AP restoration could also be tested by calculating V_m_ error *post hoc* as the difference of V_m_ over time for the 4AP-affected (4AP) and restored (4AP + APqr) APs as compared to the reference V_m_ (i.e., the average CTL AP) during the time course of an AP (between the moment of dV/dt_max_ and the moment of APD_90_). V_m_ error was positive during most of the AP in the 4AP group, with a peak of 28.6 ± 17.7 mV at 62 ms (Figures 2C and S2A), a median value of 20 mV, and V_m_ error distributing between −2.5 and 2.5 mV only 1.4% of the time (Figure 2D). With APqr ON, 92.5% of the V_m_ error data points fell in the range of −2.5 to 2.5 mV, and the median was reduced to −0.02 mV (Figure 2D).

#### AP shape restoration by single-actuator optogenetics in single cells

**Figure 3:**
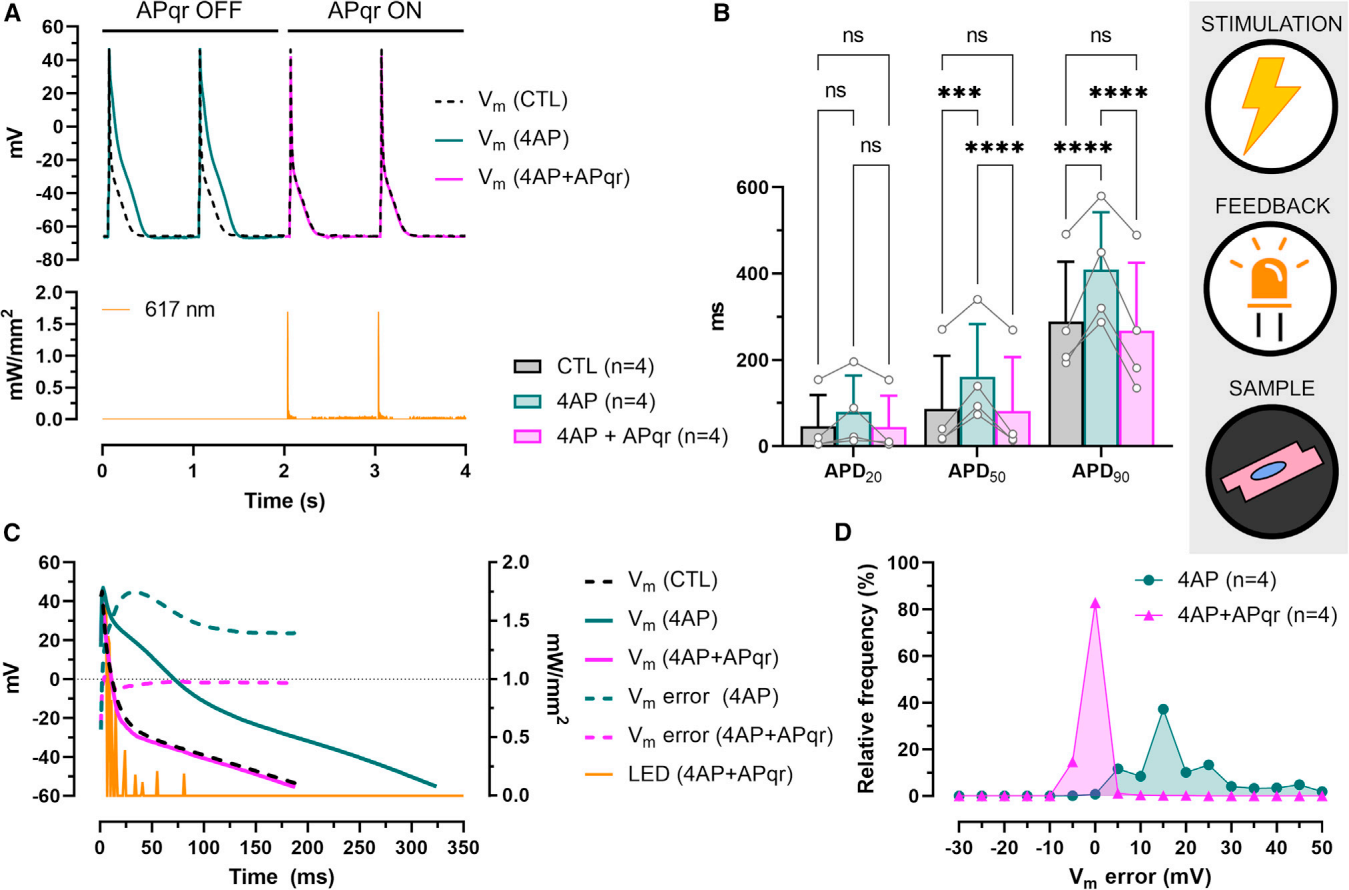
AP shape restoration by single-actuator optogenetics in single cells (A) Representative recordings of electrically triggered APs (upper panel) in a single hiAM expressing Jaws, under normal conditions (CTL), following 4AP administration (4AP), and during APqr intervention in the presence of 4AP (4AP + APqr), and of the output of the 617-nm LED (lower panel). (B) Mean APD values ±SD measured at 20%, 50%, and 90% repolarization in n = 4 single hiAMs. ∗∗∗p < 0.001, ∗∗∗∗p < 0.0001, ns: not significant. (C) Representative recordings of APs shown between the moment of dV/dt_max_ and the moment of APD_90_, overlaid with V_m_ error calculated for the drug-affected (4AP) and restored (4AP + APqr) APs compared to CTL. Also shown is the 617-nm light output during AP shape restoration. (D) Frequency distribution of V_m_ error in n = 4 single hiAMs before (4AP) and during AP shape restoration (4AP + APqr) with a 5-mV bin width. See also Figures S2B and S2E–S2G.

In order to overcome the limitations of the direct current injection approach, we next tested the feasibility of closed-loop-controlled AP restoration by using optogenetics for V_m_ control. To this end, V_APqr_ was used to modulate light output of a 617-nm light-emitting diode (LED) via V_LED_ (Figures 1C and S1A). In these experiments, single hiAMs were genetically modified by lentiviral gene delivery to express Jaws (Figure S1D). As a light-activatable inward chloride pump, Jaws uses photonic energy to create an inward flux of chloride ions, thereby moving V_m_ in the negative direction. Illumination of Jaws-hiAMs via the microscope objective resulted in a V_m_ shift of up to −25 mV from the resting potential in a largely voltage-independent but light intensity-dependent manner (Figures S2E–S2G). 4AP induced a marked widening of AP shapes, which was reversed by APqr (Figure 3A). In more detail, APD_50_ and APD_90_ values were significantly prolonged in response to 4AP treatment (p < 0.005, n = 4), while no statistically significant difference was found in APD values in 4AP-treated cells subjected to APqr (4AP + APqr) as compared to CTL (p > 0.05, n = 4, Figure 3B). Average APD_90_ was 289.5 ± 138.1 ms in control cells, 408.7 ± 133.4 ms in 4AP-treated cells without APqr, and 268.3 ± 157.2 ms in 4AP-treated cells with APqr. As illustrated by the representative recordings shown in Figure 3C, APqr drastically reduced the marked positive V_m_ error for 4AP-affected APs for most of the APD (Figures 3C and S2B). Notably, light output was highest during the early repolarization phase following the AP peak. Without APqr correction, 98.6% of V_m_ error values obtained from n = 4 cells were larger than 2.5 mV with a median of 15.7 mV. The APqr intervention, however, resulted in V_m_ error distributing around zero (0 ± 2.5) mV 83% of the time with a median value of −0.2 mV (Figure 3D).

#### AP shape restoration by dual-actuator optogenetics in multicellular preparations

To realize bidirectional V_m_ control, hiAMs were genetically modified to express both CheRiff and Jaws simultaneously by lentiviral gene transfer (Figures S1D and S1E). Once activated, the robust cation conductance created by CheRiff shunts V_m_ toward the reversal potential of the CheRiff current, thereby depolarizing the cell. CheRiff and Jaws could be selectively activated using light of 470 and 617 nm, resulting in depolarizing and re- or hyperpolarizing effects, respectively. In monolayers of CheRiff- and Jaws-expressing hiAMs, maximum intensity illumination (1.5 mW/mm^2^) by the 470-nm and 617-nm LEDs resulted in −11.2 mV and −82 mV plateau potentials on average, as observed at the end of a 1-s light pulse, respectively (V_plateau_, Figure S2H).

To implement dual-actuator closed-loop control, the APqr algorithm was modified to operate on two separate output channels. This control algorithm was dubbed APqrPID and developed based on the systems control principle known as proportional-integral-derivative (PID) controller.^33^ Similarly to APqr, APqrPID logs and stores V_m_ data consisting of an average control AP as a series of setpoint values. V_APqr_ is computed online during the correction phase and is used to modulate either of the two LEDs (Figures 1C and S1A).

#### AP restoration in the presence of a light-induced electrical disturbance in multicellular preparations

**Figure 4:**
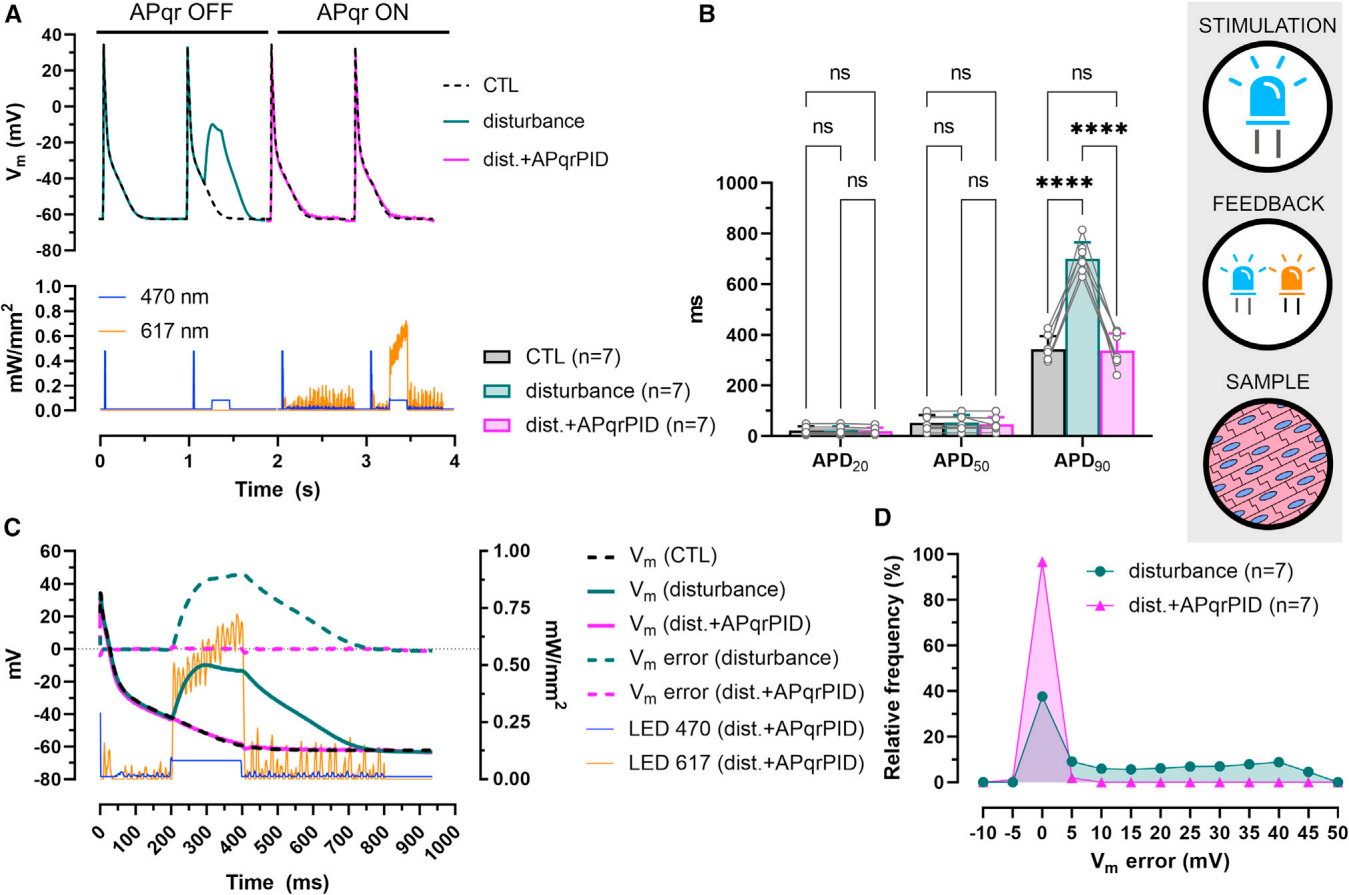
AP restoration in the presence of a light-induced electrical disturbance by dual-actuator optogenetics (A) Representative recordings of optogenetically triggered APs (upper panel) in a hiAM monolayer, under normal conditions (CTL) and in the presence of a light-induced disturbance without (disturbance) and with restoration (dist. + APqrPID). Also shown is the light output of the 470- and 617-nm LEDs during AP shape restoration (lower panel). (B) Mean APD values ±SD measured at 20%, 50%, and 90% repolarization in n = 7 hiAM monolayers. ∗∗∗∗p < 0.0001, ns: not significant. (C) Representative recordings of APs between the moment of dV/dt_max_ and the moment of APD_90_, overlaid with V_m_ error calculated for the disturbed (disturbance) and restored (dist. + APqrPID) APs compared to CTL. Also shown are the 470- and 617-nm light outputs during AP shape restoration. (D) Frequency distribution of V_m_ error in n = 7 monolayers before (disturbance) and during AP shape restoration (dist. + APqrPID) with a 5-mV bin width See also Figures S2C, S2H and S3A.

APqrPID was first used to neutralize light-induced AP abnormalities in CheRiff- and Jaws-expressing hiAMs. In these experiments, every 5^th^ AP was perturbed by a 200-ms light pulse (470 nm, 0.1 mW/mm^2^) given 200 ms after the pacing stimulus (1 Hz, 10 ms, 470 nm, 0.5 mW/mm^2^) to create abnormal cation influx during hiAM repolarization. This allowed the assessment of AP restoration efficacy in the presence of a sudden (i.e., unexpected) electrical perturbance. The representative recordings in Figure 4A illustrate that the preprogrammed extra light pulse induced a marked deflection of V_m_ resulting in an abnormal AP shape. APqrPID prevented this deflection by the autonomous (i.e., not predetermined) activation of the 617-nm LED output and restored AP morphology to normal (also shown on separate axes in Figure S3A). This was reflected in APD_90_ values, which increased from 342.9 ± 51.7 ms in CTL to 700.8 ± 64.5 ms after AP disturbance (n = 7, p < 0.0001). With the APqrPID intervention, the average APD_90_ was 338.3 ± 68 ms in the presence of the disturbance, differing only by 4.6 ms on average compared to CTL (Figure 4B). Without correction by APqrPID, V_m_ error (i.e., difference between measured and setpoint V_m_) started to increase at the onset of the perturbing light pulse and reached a maximum of 43.3 mV on average (Figures 4C and S2C), distributing around zero (0 ± 2.5) mV in only 37.6% of the time with a median of 10 mV (Figure 4D). With APqrPID correction applied, V_m_ error fell in the range 0 ± 2.5 mV 96.7% of the time with a median of 0 mV.

#### AP restoration in the presence of a drug-induced electrical disturbance in multicellular preparations

**Figure 5:**
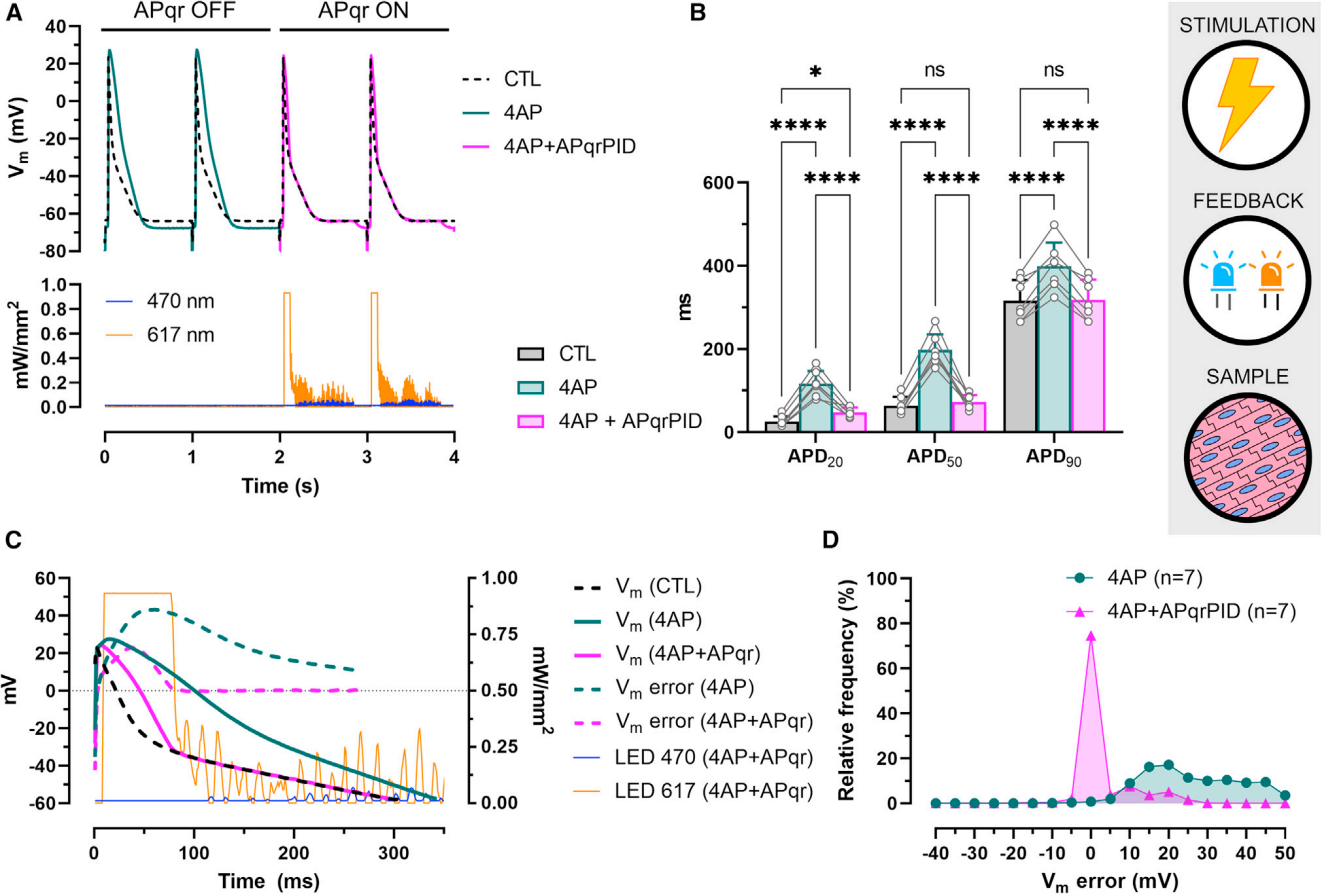
AP restoration in the presence of a drug-induced electrical disturbance by dual-actuator optogenetics (A) Representative recordings of electrically triggered APs (upper panel) in a hiAM monolayer, under normal conditions (CTL), following 4AP administration (4AP), and during AP shape restoration in the presence of 4AP (4AP + APqrPID). Also shown is the light output of the 470- and 617-nm LEDs during AP shape restoration (lower panel). (B) Mean APD values ±SD measured at 20%, 50%, and 90% repolarization in n = 7 hiAM monolayers. ∗p < 0.05, ∗∗∗∗p < 0.0001, ns: not significant. (C) Representative recordings of APs between the moment of dV/dt_max_ and the moment of APD_90_, overlaid with V_m_ error calculated for the 4AP-exposed (4AP) and vs. CTL (4AP) and 4AP vs. CTL (4AP + APqrPID) APs. Also shown are the 470- and 617-nm light outputs during AP shape restoration. (D) Frequency distribution of V_m_ error in n = 7 monolayers before (4AP) and during AP shape restoration (4AP + APqrPID) with a 5-mV bin width. See also Figures S2D, S2H and S3B.

Next, the efficacy of AP restoration by APqrPID was tested in the setting of drug-induced APD prolongation. To this end, 4AP was applied to hiAM monolayers expressing CheRiff and Jaws. Similarly to single hiAMs, 4AP induced marked APD prolongation, while the dual-actuator APqrPID intervention restored AP shapes to normal (Figure 5A, also shown on separate axes in Figure S3B). Average APD was increased by 91.2 ± 19, 134.6 ± 19.4, and 82.4 ± 27.8 ms with 4AP compared to CTL at 20%, 50%, and 90% repolarization levels, respectively (Figure 5B). APqrPID reduced APD difference significantly to 22.4 ± 11.8 ms (APD_20_), 9.1 ± 16.8 ms (APD_50_), and to as low as 1.9 ± 3.1 ms (APD_90_) on average. As the representative recordings in Figure 5C illustrate, the APD prolongation caused by 4AP administration resulted in a positive V_m_ error up to 50 mV. This error instructed APqrPID to generate predominantly red light to activate Jaws, which in turn generated the outward current favoring repolarization, counteracting the effects of 4AP. Interestingly, while the maximal V_m_ error was reduced to 30.4 mV by APqrPID, V_m_ was not reversed to normal during the early repolarization phase. During this time, red light output was maximal, indicating that total Jaws conductance was insufficient. As soon as less than maximal red light output was necessary (from 78 ms onward, Figures 5C and S2D), a better efficacy of V_m_ correction could be achieved. V_m_ error indicated a V_m_ deviation larger than 2.5 mV 98.5% of the time with a median at 24.2 mV with 4AP compared to CTL (Figure 5D). With the application of APqrPID, V_m_ deviation was larger than 2.5 mV in only 21.9% of the cases and fell within −2.5 to 2.5 mV 74.7% of the time. The median value of residual V_m_ error during APqrPID intervention was 0.23 mV. Taken together, these results provide experimental evidence for the feasibility of accurate, closed-loop-controlled restoration of abnormal AP morphologies using two antagonizing optogenetic actuators in a multicellular preparation.

### Light-driven enforcement of arbitrary AP morphologies

A method for the control of AP waveforms as presented in this study not only allows normalization of disturbed AP shapes, but it may also provide means to enforce any desired AP morphology onto cardiomyocytes in an autonomous, self-regulatory manner. To investigate this possibility, the APqrPID closed-loop control algorithm was modified in two aspects. Firstly, instead of recording reference APs under control conditions, the new controller dubbed APqrLE loads data defining reference APs (also referred to as model APs) from a file. Secondly, APqrLE produces intermittent light output (470 nm, 10 ms at 1 Hz in our experiments) in order to activate CheRiff thereby triggering APs.

#### Imposing models of drug-induced AP perturbations

**Figure 6:**
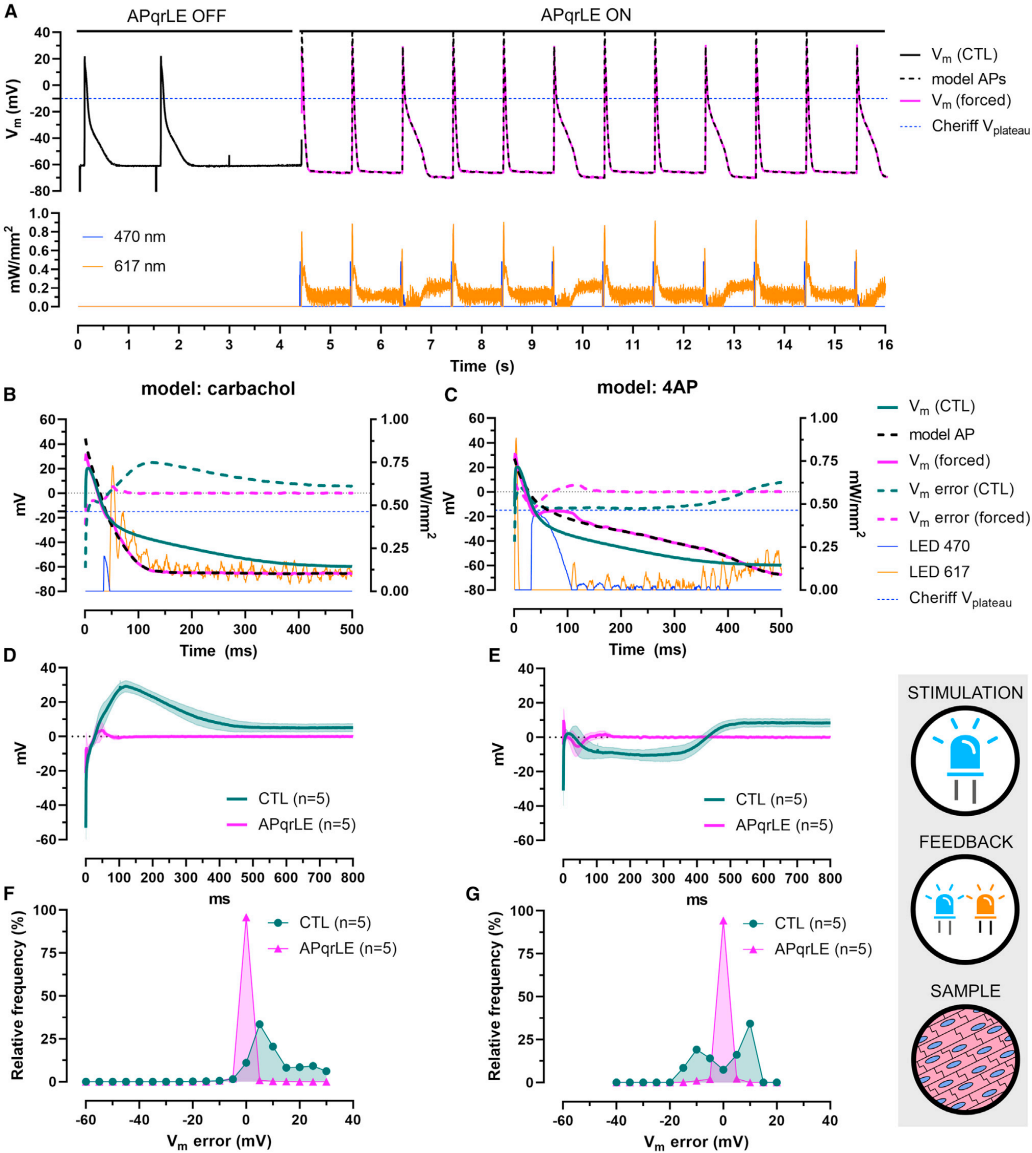
Imposing models of drug-induced AP perturbations in hiAM monolayers (A) Representative recordings of electrically triggered endogenous APs (CTL) and V_m_ during enforcement of models of carbachol- and 4AP-induced AP morphologies in a hiAM monolayer expressing CheRiff and Jaws (upper panel) and display of the 470- and 617-nm light output (lower panel). (B and C) Representative recordings of APs between the moment of dV/dt_max_ and the moment of APD_90_, overlaid with the corresponding AP model and V_m_ error calculated for the CTL (CTL) and enforced (APqrLE) APs compared to the corresponding model APs, for the carbachols and 4AP models, respectively. Also shown are the 470- and 617-nm light outputs during AP shape enforcement. Labels shown in (C) apply to (B) as well. (D and E) V_m_ error representing the difference between model vs. endogenous APs (CTL) and model vs. enforced APs (APqrLE) over time for the carbachol and 4AP models, respectively, in n = 5 hiAM monolayers. Data are shown as mean (continuous line) ±SD (shaded area). (F and G) Frequency distribution of V_m_ error in n = 5 monolayers before (CTL) and during AP shape enforcement (APqrLE) with a 5-mV bin width for the carbachol and 4AP models, respectively. See also Figures S3C, S3D, and S4.

Prior to the experiments with APqrLE, APs were recorded in hiAM monolayers exposed to carbachol, an acetylcholine receptor agonist (i.e., a drug shortening APD) or to 4AP and were used as model APs during AP waveform enforcement in n = 5 monolayers of hiAMs expressing CheRiff and Jaws (Figure 6). The model APs were enforced in the carbachol-carbachol-4AP order.

As the representative recordings in Figure 6A illustrate, hiAM monolayers were first paced electrically with APqrLE switched off to register endogenous APs. Then the electrical stimulation was terminated, and APqrLE was activated. With APqrLE running, APs were triggered by blue light output every second. Following the pacing stimuli, a varying mixture of blue and red light output was generated, and coinciding with this, the real-time measured V_m_ was following the shape of the model APs (Figures S3C and S3D). It is important to note that while the inward chloride pump Jaws hyperpolarizes V_m_ in an essentially V_m_-independent manner, a depolarizing effect from the light-gated ion channel CheRiff can only be expected when V_m_ is more negative than the reversal potential of the CheRiff current. This apparent reversal potential for CheRiff was characterized as V_plateau_ for each monolayer (Figure S2H) and was set as a parameter for APqrLE in each experiment. Blue light output was only permitted when V_m_ was more negative than CheRiff’s V_plateau_ (Figure 6A).

While average APD measured at 30% and 50% repolarization levels were statistically not different between model and control APs, average APD_90_ was significantly shorter in the carbachol model (95.2 ± 0.4 ms) and prolonged in the 4AP model (422.2 ± 0.4 ms) compared to CTL (314.6 ± 53.1 ms, p < 0.05, Figures S4A and S4B). With APqrLE on, average, APD_90_ was shortened to 89.6 ± 3.9 ms and prolonged to 418.4 ± 3.6 ms during the enforcement of the carbachol and the 4AP model, respectively (Figures S4A and S4B). Hence, APqrLE reduced the APD_90_ differences between control and model APs from 219.4 to 107.6 ms to 5.6 and 3.8 ms for the carbachol and 4AP model, respectively. Analysis of maximal V_m_ (V_peak_) and dV/dt_max_ showed that the enforcement of model APs was the least effective during the AP upstroke (Figures S4C and S4D). Since the average time to reach dV/dt_max_ was ∼5 ms longer for forced compared to model APs (Figure S4E) and because this time point is used to calculate APD values, it may explain the slight APD_90_ differences that remain observable during AP enforcement. To account for this, the times at which the various repolarization levels occurred (i.e., the times at which V_m_ took the value corresponding to 30%, 50%, or 90% repolarization) were determined and compared between reference and forced APs (Figures S4G and S4H). This comparison showed the absence of statistical differences in time of APD_30_, APD_50_, and APD_90_ between the model APs and their enforced counterparts, with time of 90% repolarization differing as little as 1.6 and 0.4 ms for the carbachol and 4AP models, respectively (Figures S4G and S4H). Average V_rest_ values were significantly more positive in CTL compared to the carbachol (5 mV difference) and 4AP (8.7 mV difference) models. During enforcement, V_rest_ values of the corresponding models were imposed with as little as 0.005 and 0.01 mV difference on average (Figure S4F).

As the representative recordings show, predominantly red light was necessary to reproduce the carbachol model and blue light for the 4AP model (Figures 6B and 6C, also shown on separate axes in Figures S3C and S3D). In both cases, the inescapable binding of the blue light output to the V_m_ range negative of CheRiff’s V_plateau_ limited the efficacy of V_m_ control during the early phase of the AP. In the carbachol model, enforcement reduced the average V_m_ error to less than 5.5 and 2.5 mV at 16 and 22 ms after reaching dV/dt_max_, respectively (Figure 6D). For the 4AP model, average V_m_ error levels below 5.5 and 2.5 mV were reached within 5 and 62 ms, respectively (Figure 6E). V_m_ error calculated for control APs compared to the carbachol and 4AP reference APs had median values of 7.8 and 2.7 mV, distributing within the range of −2.5 to 2.5 mV in 11% and 7.5% of the time, respectively. During the enforcement of the carbachol and 4AP models, V_m_ error fell in the range of 0 ± 2.5 mV in 95.8% and 94.4% of the time, and the median V_m_ error was reduced to −0.02 and 0.04 mV, respectively (Figures 6F and 6G).

#### Imposing simplified AP models

Next, the operational range of AP morphology control as carried out in our study was assessed by enforcing simplified AP waveforms by APqrLE in hiAM monolayers expressing CheRiff and Jaws (n = 5). Each of the AP models consisted of two linear phases, an upstroke and a repolarization phase. The upstroke of each model arose from −68 mV at a rate of 20 mV/ms to the V_peak_ at 12 mV, after which the model V_m_ started to decrease at constant rates of 1.3, 0.9, 0.5, or 0.1 mV/ms. The resulting triangular AP models were named as t13, t09, t05, and t01, respectively, and they were enforced in a consecutive manner following each other in the t13, t09, t05, t01, t05, t09, and t13 order.

**Figure 7:**
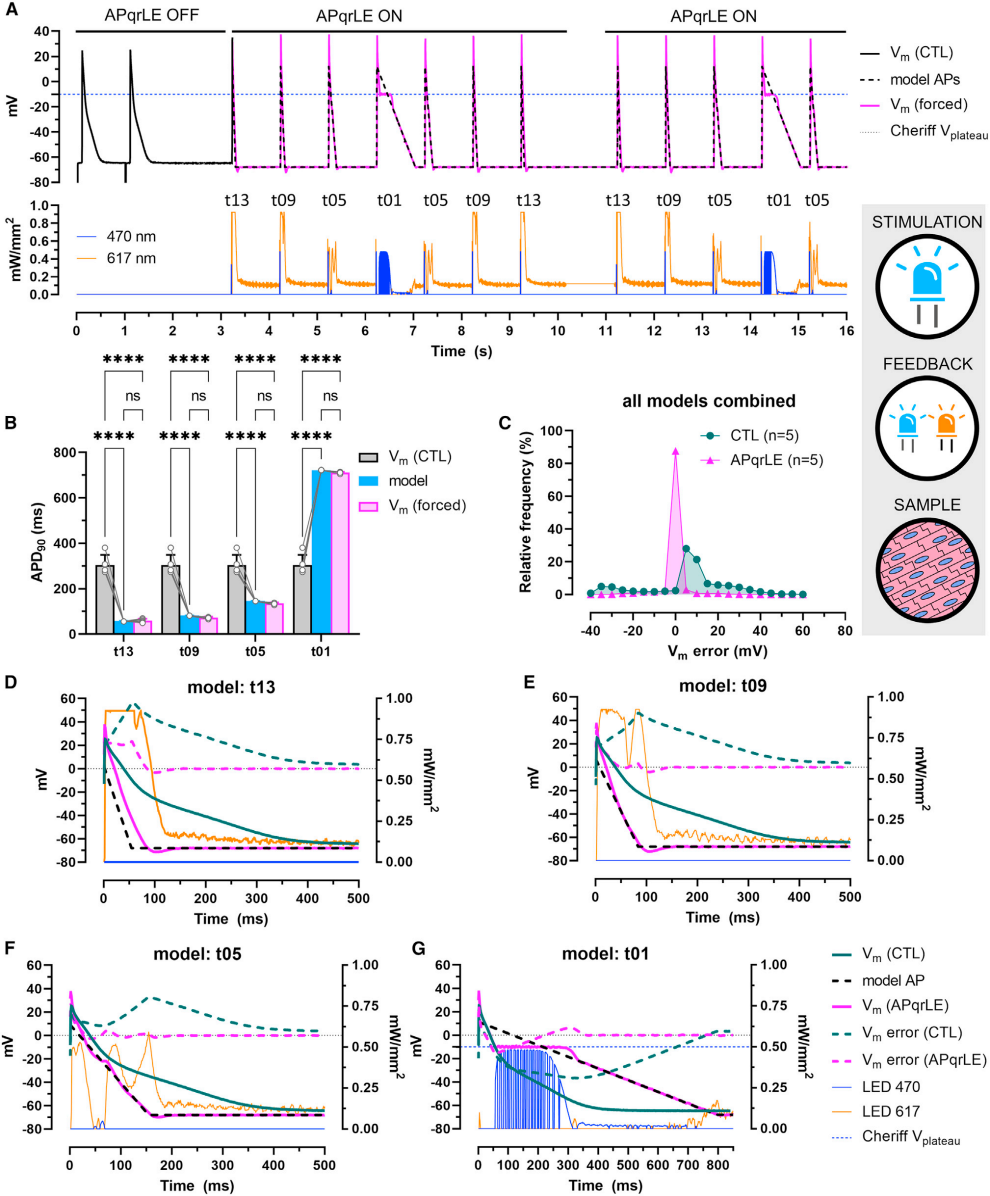
Imposing simplified AP models in hiAM monolayers (A) Representative recordings of electrically triggered endogenous APs (CTL) and V_m_ during enforcement of AP models t13, t09, t05, and t01 in a hiAM monolayer expressing CheRiff and Jaws (upper panel) and display of the 470- and 617-nm light output (lower panel). (B) Mean APD values ±SD measured at 20%, 50%, and 90% repolarization in n = 5 hiAM monolayers. ∗∗∗∗p < 0.0001, ns: not significant. (C) Frequency distribution of V_m_ error for all AP models combined in n = 5 monolayers before (CTL) and during AP shape enforcement (APqrLE) with 5-mV bin width. (D–G) Representative recordings of APs between the moment of dV/dt_max_ and the moment of APD_90_, overlaid with the corresponding AP model and V_m_ error calculated as the difference between CTL and model APs (CTL) and enforced and model APs (APqrLE) for the t13, t09, t05, and t01 models, respectively. Also shown are the 470- and 617-nm light outputs during AP shape enforcement. Labels shown in (G) apply to (D)–(F) as well. See also Figures S5–S7.

As before, hiAM monolayers were first paced electrically to register endogenous (control) APs, after which APqrLE was activated, and the light-driven model AP enforcement started (Figures 7A and S5). Due to different repolarization rates, average APD_90_ values of model APs differed substantially from that of the endogenous APs (Figures 7B and S6A–S6D). The difference between APD_90_ of the model APs versus CTL was 245.8, 221.8, 158, and 418.4 ms on average, which was reduced during V_m_ enforcement by APqrLE to 1.8, 8.8, 10.2, and 11 ms for models t13, t09, t05, and t01, respectively (Figure 7B). Notably, such outcome necessitated forced APD_90_ shortening for models t13, t09, and t05 and prolongation for model t01. The residual APD_90_ difference could be attributed to the fact that the upstroke phase of forced APs followed the model APs the least, as illustrated by V_peak_, dV/dt_max_, and time to reach dV/dt_max_ (Figures S6E–S6G). Indeed, the time of 90% repolarization deviated by less than 1 ms between model and forced APs for model t01 (0.6 ms) and t05 (−0.2 ms), and the difference was only 1.6 ms for model t09 on average, while it was enforced the least accurately in the case of model t13 (−13.4 ms difference, Figure S6H). Apparently, the maximal ion flux produced by Jaws did not always suffice to fully impose model t13 as well as the early phase of model t09, as indicated by the red light output reaching its maximum during the enforcement of these AP models (Figures 7D and 7E, also shown in Figure S5 on separate axes). Resting potentials (V_rest_) were more positive in control APs compared to model APs (6.9 mV difference, n = 5, p < 0.05), while V_rest_ during enforcement differed by less than 0.7 mV on average (Figure S6I).

V_m_ error was reduced to the range of −2.5 to 2.5 mV within the first 50 ms for models t09 and t05 (Figures 7E**,** 7F**,** S7B, and S7C) and within the first 80 ms for model t13 (Figures 7D and S7A) during the generation of forced APs. V_m_ error was reduced to fall within this range during the enforcement of model AP t01 as well, albeit only after 280 ms on average (Figures 7G and S7D). This was due to the fact that CheRiff could only produce a depolarizing current below its V_plateau_. V_m_ error calculated for control versus model APs of all four models combined had a median value of 7.5 mV with 28% of data points distributing around zero (0 ± 2.5) mV (Figure 7C). Despite the aforementioned limitations, the APqrLE intervention reduced the median V_m_ error to −0.005 mV, with 87.6% of data points falling within the range of 0 ± 2.5 mV (Figure 7C). The relatively high occurrence of residual V_m_ error is due to the ion channel nature of CheRiff, which affected the enforcement of the t01 model the most (Figure S7H). For all other models, the frequency of V_m_ error between −2.5 and 2.5 mV was higher than 92% (Figures S7E–S7G).

Taken together, these data illustrate the feasibility of dynamic enforcement of arbitrary AP waveforms in multicellular cardiomyocyte preparations by a closed-loop-driven dual-actuator optogenetic approach.

## Discussion

In this study, we realized accurate restoration and preservation of cardiac AP waveforms in the presence of electrical perturbations of different origin in an unsupervised, self-regulatory manner, without any prior knowledge of the disturbance. We achieved this by the design and realization of an experimental system consisting of elements from patch-clamp electrophysiology, hard real-time computing, and optogenetics integrated in a closed-loop arrangement. Feedback loop control was accomplished by three different custom algorithms, APqr, APqrPID, and APqrLE. APqr was designed to target V_m_ by means of direct current injection or activation of a single optogenetic actuator by light. The PID closed-loop control principle was implemented in APqrPID designed to operate on two optogenetic actuators with antagonizing effects. APqrLE was based on PID control principles as well and designed to enforce arbitrary AP waveforms. We showed effective correction of AP morphology changes, particularly correction of APD prolongation both by current injection and illumination in single cardiomyocytes using APqr and in monolayers of cardiomyocytes using dual-actuator optogenetics by APqrPID. Effective V_m_ control in cardiomyocyte monolayers was further evidenced by the enforcement of arbitrary AP shapes, including AP morphologies with drug-induced abnormalities and simplified, triangle-like AP models by the application of APqrLE.

Feedback loop-driven system control has several beneficial features. Most importantly, it enables delivery of interventions on demand (i.e., only when needed), which can be especially useful in the management of conditions that manifest in an episodic manner, such as vasovagal syncope, intractable neuropathic pain, or epilepsy.^34^^,^^35^ On the other hand, the inherent self-regulatory nature of closed-loop systems’ control eliminates the need of extensive prior calibration and allows the use of actuators with non-linear input-output relationships, such as ChRs.^11^ In the experimental setting, feedback loops implemented in “closed-loop optogenetics” have opened new avenues for the interrogation of neuronal activity in behaving animals and have already seen several applications.^36^^,^^37^ In general, such approaches aim to modulate the activity of single neurons or neuronal circuits (in essence their firing rate) by means of optogenetic stimulation in response to physiological, electrical, or optical readouts. Recent developments in the field have been focusing on the realization of all-optical closed-loop platforms, which combine optical readout and photostimulation.^24^^,^^38^ These systems have the unique capacity to interrogate neural circuits in living animals in a fully non-invasive way. However, the low signal-to-noise ratio of the optical V_m_ readout only allows relatively low closed-loop update rates (i.e., the rate at which V_m_ input is sampled and light output is generated), preventing the control of fast processes with physiologically relevant accuracy. Here, we reasoned that by improving the temporal resolution of closed-loop optogenetics, it would be possible to fully control excitability, including not only the frequency of activated states (i.e., the firing rate of neurons or the heart rate) but also the shape of individual cardiac APs. We sought to achieve this by ensuring sufficiently fast feedback loop update rates. For this reason, we opted for patch-clamp electrophysiology, capable of acquiring V_m_ readout at high sampling rates. This strategy made an update rate of 1 kHz possible, which was key to achieve millisecond and millivolt accuracy V_m_ control.

### Limitations of the study

While demonstrating the ability to control V_m_ in cardiomyocytes with physiologically relevant accuracy and precision by dual-channel closed-loop optogenetics in the multicellular setting, we also identified key areas where further development is needed in order to exploit the beneficial features of the technology presented here in full.

Due to the electrical coupling between cardiomyocytes, an AP recorded by the patch-clamp technique from a specific location within a hiAM monolayer represents a so-called compound AP, the summation of APs generated by cells in and surrounding that location, which is expected to be uniform in all areas of the monolayer. However, local electrical or optogenetic pacing results in activation waves spreading from the site of pacing through the monolayer. Consequently, V_m_ control by APqr, as carried out in this study, was optimal at the site of recording but not in the whole monolayer. This limitation could be overcome, on the short term, by using homogeneous light sources delivering the same light intensity throughout the entire monolayer. Optogenetic pacing by a homogeneous light source would eliminate differences in local activation times. Because of the synchronous activation, the homogeneously applied corrective light output from APqr would result in optimal V_m_ control for the entire monolayer, despite the dependence on local V_m_ readout. Alternatively, recent developments in nanotechnology, such as 3D nanoelectrode arrays, could be exploited in order to obtain local, intracellular V_m_ recordings.^39^ In combination with patterned photostimulation^40^ and the APqr technology, this would allow high-resolution V_m_ control in both space and time.

The performance of PID controllers is largely determined by three mutually dependent gain parameters K_P_, K_D_, and K_I_ (Equation 10). Setting optimal values for the gain parameters (i.e., controller tuning) is typically carried out by established procedures, such as the so-called Ziegler-Nichols method. We found that using gain parameters determined by this method produced suboptimal outcomes, such as ineffective V_m_ control or oscillations, likely due to the non-linear dependence of V_m_ on effector output and the fast kinetics of V_m_ changes during the AP. Therefore, in our experiments, controller tuning was finalized by a mainly heuristic approach, which typically resulted in different gain parameters in each experiment (K_P_: 40–120, K_I_: 1–5, K_D_: 5–200). The optimal values of gain parameters and their dependence on experimental conditions could be determined in dedicated studies, which would potentially result in further improvements in controller performance. Alternatively, auto-tuning principles could be implemented in the closed-loop controller, which would allow controller tuning in an unsupervised way, further strengthening the applicability of our approach.^33^

In addition, the selection of optogenetic actuators could be tailored to each specific application. First, the permeability of the opsin of choice to different ionic species should be considered with respect to the potential effects of changes to the ionic milieu occurring in response to opsin activation. For example, the inward chloride pump Jaws could be replaced by outward proton pumps in cases where cytosolic chloride concentrations are critical.^21^ Also, the relative cation conductance of ChR variants can be considered.^41^ Furthermore, the operational range of the experimental system presented here could be extended, primarily by the use of optogenetic actuators with functional properties matching a particular application. For example, the V_m_ range in which depolarizing effects are available could be broadened by replacing CheRiff by an optogenetic actuator that facilitates active inward cation or outward anion transport in a V_m_-independent fashion (i.e., an ion pump). Promising alternatives are the light-activatable inward proton pumps belonging to the families of xenorhodopsins and schizorhodopsins.^42^^,^^43^ However, the action spectrum of SzR3 centered at 525 nm overlaps with known optogenetic actuators having hyperpolarizing effects, which prevents application of SzR3 in the dual-actuator arrangement. The recent rapid development of microbial rhodopsin research, including both the discovery of natural variants and deeper understanding of structure-function relationships for rational color tuning, holds the promise of overcoming this limitation in the near future.^44^ Furthermore, Jaws could be directly replaced by the most recently described potassium ChR KCR1.^45^ KCR1 can be activated by red light (action spectrum peak: 540 nm), allowing application in combination with blue light-activatable actuators. Importantly, displaying a reversal potential at −85 mV, KCR1 is expected to generate large outward current amplitudes driven by the great driving force for potassium in the positive V_m_ range. These characteristics hint that sufficient hyperpolarizing effect could be achieved by using KCR1 during the early phase of repolarization, when Jaws function appears to be the least optimal.

Once these limitations have been dealt with effectively, we foresee imminent applications of opto-electronic closed-loop-controlled systems for V_m_ control in various research areas, These could range, for example, from the regulatory roles of V_m_ in cellular differentiation and homeostasis to its impact on disease development and progression. As to the latter subject, a hallmark of many heart diseases including atrial fibrillation and heart failure is electrical remodeling.^46^^,^^47^ The resulting changes in the electrophysiological properties of the heart are often associated with deleterious consequences. In atrial fibrillation, for example, rapid electrical activation increases Ca^2+^ exposure of atrial myocytes, which triggers compensatory mechanisms leading to refractory period shortening as a manifestation of electrical remodeling, which in turn increases atrial fibrillation propensity.^48^ In heart failure, electrical remodeling includes the loss of the “notch” in the “spike-notch-dome”-shaped APs typically displayed by healthy ventricular myocytes. Interestingly, clamping V_m_ of healthy cells to a failing AP shape has robust influence on Ca^2+^ handling in isolated single cells in the acute setting.^49^ While such association of electrical remodeling to disease conditions is well documented in many cases, the cause and effect relationships and the detailed molecular mechanisms remain elusive. Using APqr, cardiomyocytes cultured in multicellular format could, for example, be exposed to AP morphologies characteristic of pathological conditions, which would allow dissection of cause and effect, while creating possibilities to study the underlying mechanisms in a controlled and detailed manner.

Collectively, this study sets the stage for the refinement and application of opto-electronic control systems to enable in-depth investigation into the regulatory roles of V_m_ in health and disease.

## STAR★Methods

### Key resources table

**Table:** 

REAGENT or RESOURCE	SOURCE	IDENTIFIER
**Bacterial and virus strains**		

LV.HsUBC.Jaws ∼ eGFP.IRES.PurR.hHBVPRE	this paper	N/A
LV.GgTnnt2.CheRiff ∼ eGFP.WHVoPRE	this paper	N/A

**Chemicals, peptides, and recombinant proteins**		

NaCl	Merck	Cat# 106404
KCl	Merck	Cat# 104936
CaCl_2_	Merck	Cat# 102382
MgCl_2_	Merck	Cat# 105833
HEPES	VWR	Cat# 0511
NaOH	Merck	Cat# 106498
KOH	Merck	Cat# 5021
Amphotericin B	Sigma-Aldrich	Cat# A2411
Potassium D-gluconate	Merck	Cat# G4500
4-Aminopiridine	Sigma-Aldrich	Cat# 275875
Carbachol (Miostat, 0.1 mg/ml)	Alcon	N/A

**Experimental models: Cell lines**		

hiAM cell line	Harlaar et al.^32^	N/A
Jaws-hiAM cell line	this paper	N/A

**Recombinant DNA**		

Jaws-KGC-GFP-ER2	Addgene	Addgene_65012
CheRiff-eGFP	Addgene	Addgene_51693
pLV.HsUBC.Jaws ∼ eGFP.IRES.PurR.hHBVPRE	this paper	N/A
pLV.GgTnnt2.CheRiff ∼ eGFP.WHVoPRE	this paper	N/A

**Software and algorithms**		

RTXI	Patel et al.^50^	http://rtxi.org
APqr-software	this paper	https://doi.org/10.5281/zenodo.10116142
Python version 3.3	Python Software Foundation	https://www.python.org

### Resource availability

#### Lead contact

Further information and requests for resources and reagents should be directed to the lead contact, Daniël A. Pijnappels (d.a.pijnappels@lumc.nl)

#### Materials availability

Materials can be obtained from the lead contact after appropriate scientific review and a completed material transfer agreement.

#### Data and code availability

• Patch-clamp recordings reported in this paper will be shared by the lead contact upon request.
• All original code has been deposited at Zenodo: https://doi.org/10.5281/zenodo.10116142.
• Any additional information required to reanalyze the data reported in this paper is available from the lead contact upon request.

### Experimental model and study participant details

#### hiAM cell culture

Proliferation and differentiation of hiAMs were carried out as previously described.^32^ Briefly, hiAMs were proliferated in the presence of doxycycline (DOX, 100 ng/mL, Sigma-Aldrich). For differentiation, hiAMs were seeded on bovine fibronectin (Sigma-Aldrich)-coated glass coverslips in 24-well culture plates at a density 3.75×10^5^ cells per well and cultured in medium without DOX. Experiments on monolayers were performed between day 12 and 14 of differentiation on coverslip-adhered hiAM monolayers transferred directly to the patch clamp bath. For single cell experiments, hiAM monolayers were dissociated on day 12 of differentiation by papain treatment. Dissociated hiAMs were re-seeded at a density of 2×10^4^ cells per well of 24-well culture plates and allowed to adhere to freshly coated coverslips overnight.

The hiAM proliferation medium consisted of Advanced DMEM/F-12 (Thermo Fisher Scientific, 12634), 2 mM GlutaMAX (Thermo Fisher Scientific), 2% FBS (Biowest), 100 units/mL of penicillin and 100 μg/mL of streptomycin (Thermo Fisher Scientific) and DOX (100 ng/mL, Sigma-Aldrich). After seeding for differentiation, the hiAM differentiation medium was used consisting of Advanced DMEM/F-12 (Thermo Fisher Scientific, 12634), 2 mM GlutaMAX (Thermo Fisher Scientific) and 2% FBS (Biowest). Starting from day 4, the hiAM differentiation medium was supplemented with triiodo-L-thyronine (20 ng/mL, Sigma-Aldrich), dexamethasone (400 ng/mL, Centrafarm), LF3 (8 μM, Selleck Chemicals) and phenylephrine (10 μM, Sigma-Aldrich).

For experiments on single cells, differentiated hiAM monolayers were dissociated on day 12 of differentiation by incubation with a solution containing 5 U/mL papain (Worthington Biochemical) and 1 mM L-cysteine (Sigma-Aldrich) in phosphate-buffered saline (PBS) for 5–10 min at 37°C, after which an equal volume of stop solution was added, consisting of 1 mg/mL soybean trypsin inhibitor (Sigma-Aldrich) and 40 μg/mL DNase I (Sigma-Aldrich) in PBS. Cells were pelleted by gentle centrifugation (150×*g*, 5 min), resuspended in hiAM differentiation medium and reseeded at a density of 2×10^4^ cells per well on coverslips freshly coated with bovine fibronectin (Sigma-Aldrich). The cells were allowed to adhere to the coverslips overnight and well-separated single hiAMs were subjected to patch clamp analysis the next day.

### Method details

#### Generation and production of lentiviral vectors

The expression plasmids encoding enhanced green fluorescent protein (eGFP)-tagged versions of CheRiff and Jaws were obtained from Addgene (Addgene plasmid numbers 51693 and 65012, respectively). CheRiff- and Jaws-coding sequences were subcloned to yield the lentiviral shuttle constructs pLV.GgTnnt2.CheRiff ∼ eGFP.WHVoPRE and pLV.HsUBC.Jaws ∼ eGFP.IRES.PurR.hHBVPRE, respectively (Figure S1), using standard laboratory techniques. Lentiviral vectors were produced using the second-generation packaging plasmids psPAX2 and pLP/VSVG as described previously.^32^

#### (Opto)genetic modification of hiAMs

A polyclonal hiAM cell line stably expressing Jaws was created by transducing 10^6^ proliferating hiAM clone 2.38 cells in one well of a 6-well culture plate with the LV.HsUBC.Jaws ∼ eGFP.IRES.PurR.hHBVPRE lentiviral vector at a transduction rate of ∼50% and subsequent selection by puromycin (2 μg/mL, Thermo Fisher Scienetific). Jaws-hiAMs were proliferated while maintaining selection pressure. For the simultaneous ectopic expression of CheRiff and Jaws, 3.75×10^5^ proliferating Jaws-hiAMs were incubated with the LV.GgTnnt2.CheRiff ∼ eGFP.WHVoPRE lentiviral vector in an *ad hoc* manner in the presence of DEAE-dextran (5 μg/mL, Carl Roth) and 100 ng/mL DOX for 24 h.

hiAMs express KCNJ5, the gene encoding an essential pore-forming subunit of I_KAch_ ion channels, at a very low level.^32^ In order to establish an AP model that represents the effects of carbachol on hiAM APs (i.e., shortened APD), a polyclonal hiAM line stably expressing KCNJ5 was established by using the LV.HsPGK1.HsKCNJ5.IRES.PurR.hHBVPRE lentiviral vector as described for Jaws-hiAMs. Jaws-, CheRiff-Jaws- and KCNJ5-hiAMs were differentiated as described above.

#### Patch clamp electrophysiology

Coverslips with single hiAMs or hiAM monolayers were transferred to a patch clamp bath situated on the sample stage of an inverted microscope (Zeiss Axiovert 35). The bath was continuously superfused with the external solution containing (in mM): 140 NaCl, 5.4 KCl, 1.8 CaCl_2_, 1.0 MgCl_2_, 5.5 glucose and 5.0 HEPES-NaOH (pH 7.4). Custom-fabricated borosilicate glass pipette electrodes were back-filled with (in mM): 125 K-gluconate, 20 KCl, 5.0 NaCl, 0.22 amphotericin B (Sigma-Aldrich) and 10 HEPES-KOH (pH 7.2) and had a tip resistance in the range of 2–4 MΩ. The calculated liquid junction potential (−15.5 mV) was left uncorrected. Bath temperature was kept constant at 25°C by means of an ITO glass bath bottom (Cell MicroControls) and an inline solution heater (Multichannel Systems). Membrane potential recordings were obtained in the perforated whole cell configuration with series resistances <50 MΩ sampled at 10 kHz. In single hiAMs, APs were triggered by brief (2–10 ms) current pulses with suprathreshold amplitudes at 1 Hz. Monolayers were paced externally at a frequency of 1 Hz via custom-made bipolar electrodes placed close to the edge of the monolayer or by diffuse blue light (470 nm) illumination (0.5 mW/cm^2^, 10-ms duration).

Light-induced whole cell Jaws currents were recorded in the ruptured patch configuration. Membrane potential was clamped at −60 mV and the series resistance was compensated electronically to >80%. Currents were sampled at 10 kHz after low-pass analog filtering using a cutoff value of 4 kHz. Capacitive currents were recorded in response to 10-mV hyperpolarizing voltage clamp steps from −40 mV. Cell size was estimated by calculating cell capacitance as the decay time constant of the capacitive transient over series resistance. Current amplitudes were normalized by the cell capacitance for each cell.

For the characterization of light responses in single cells and in monolayers, the cells were kept in the dark for 3 min to allow CheRiff and Jaws to relax to their fully dark-adapted states, after which the cells were illuminated sequentially by 1-s light pulses of increasing intensity with in-between dark periods of 30 s.

#### Instrumentation

Schematics of the experimental system is given in Figure S1A. V_m_ recordings were obtained by standard patch clamp electrophysiology using the Multiclamp 700B amplifier controlled by the pClamp software package (version 10.7.0.3) via the Digidata 1440A D/A interface (all from Molecular Devices). V_m_ was also sampled by a real-time computer hosting the Real-Time eXperiment Interface (RTXI)^50^ via the PCI-6221 D/A interface (National Instruments). Real-time computation was carried out by custom scripts written for the RTXI platform hosted on a Linux computer (Ubuntu 16.04 LTS, 32GB RAM, Intel Core i7-4790 CPU). The static analog output generated by pClamp and the dynamic output from the RTXI were used as command signal for the patch clamp amplifier or for the modulation of LED power sources (LEDD1B, Thorlabs) separately or in combination added together by a custom voltage summing amplifier.

LEDs with emission centered at 470 (M470L3-C4, Thorlabs) and 617 nm (M617L3, Thorlabs) mounted on collimation lenses (COP1-A, Thorlabs) served as light source. Single cells were illuminated via a 40× magnification objective, whereas monolayers were illuminated directly after positioning the LEDs above the patch clamp bath.

#### Closed-loop control

Closed-loop control was realized with custom scripts written in C++ language to be installed as plugin for the RTXI platform.^50^ All scripts are publicly available via https://doi.org/10.5281/zenodo.10116142.

For V_m_ control by direct current injection (Figure 2) and by single-actuator optogenetics (Figure 3), the APqr closed-loop control algorithm was used, the functionalities of which are described below (see also Figure S1B). At the beginning of each experiment, a number of consecutive APs are recorded under normal conditions and stored as V_m_ setpoint values in the array *AP*_*ideal*_, according to Equation 1:

(Equation 1) $$AP_{ideal}\left[i\right]=\frac{1}{lognum}\cdot \sum_{n=0}^{lognum}V_{m}^{n}\left[i\right]$$

where *lognum* is the total number of normal APs logged chosen by the experimenter and *i* denotes discrete time. During the correction phase, the difference between the online measured V_m_ and the setpoint value (*AP*_*ideal*_[*i*]) is calculated as V_m_ error (e) according to Equation 2:

(Equation 2) $$e=V_{m}-AP_{ideal}\left[i\right]$$

An output is then calculated according to (Equation 3), (Equation 4):

(Equation 3) $$K_{P}=C_{m}\cdot \frac{1}{R_{m}}$$

(Equation 4) $$I_{out}=K_{P}\cdot e$$

where *C*_*m*_ is cell capacitance and *R*_*m*_ is membrane resistance. *I*_*out*_ is used as command signal for the patch clamp amplifier or as modulatory signal for the LED driver. *R*_*m*_ is updated during each loop iteration depending on the conditions specified in Equation 5:

(Equation 5) $$R_{m}=\left\{ \begin{array}{c} R_{m\_corr\_up}\cdot R_{m}\;if\;V_{m}\;overshoots\;AP_{ideal}\left[i\right]\\ \frac{R_{m}}{R_{m\_corr\_down}}if\;V_{m}\;moves\;away\;from\;AP_{ideal}\left[i\right] \end{array}\right.$$

where *R*_*m_corr_up*_ and *R*_*m_corr_down*_ are constants chosen by the experimenter.

For AP morphology restoration by dual-actuator optogenetics (Figures 4 and 5), the closed-loop control algorithm APqrPID was used (Figure S1C). APqrPID takes into account the past (the ‘I’ or integral term) and the future (the ‘D’ or derivative term), in addition to the momentary value of V_m_ error (‘P’ or proportion term). Similar to APqr, APqrPID records a chosen number of APs obtained under normal conditions and stores an average AP in *AP*_*ideal*_ as setpoint values according to Equation 1. During correction, V_m_ error (*e*) is calculated as specified in Equation 2. The I term, which speeds up or slows down the rate of change of the output levels based on the history of the V_m_ error is computed in every iteration as follows:

(Equation 8) $$e_{sum}=e_{sum}+e$$

The D term observes and predicts the trajectory of V_m_ by linear regression according to Equation 9:

(Equation 9) $$e_{derivative}=\frac{l\cdot \sum_{j=0}^{l}e\left[-j\right]\cdot j\cdot \Delta t-\sum_{j=0}^{l}e\left[-j\right]\cdot \sum_{j=0}^{l}j\cdot \Delta t}{l\cdot \sum_{j=0}^{l}{\left(j\cdot \Delta t\right)}^{2}-{\left(\sum_{j=0}^{l}j\cdot \Delta t\right)}^{2}}$$

where *l* denotes the amount of adjacent data points taken into account for linear regression (selectable, set to 10 by default). And finally, the output is computed according to Equation 10.

(Equation 10) $$PID=K_{P}\cdot e+K_{I}\cdot e_{sum}+K_{D}\cdot e_{derivative}$$

where *K*_*P*_, *K*_*I*_ and *K*_*D*_ are gain parameters. When PID takes a negative sign, its absolute value is used for the blue light source (depolarizing effect) and a PID with positive sign is used for the red light source (hyperpolarizing effect) as LED driver modulatory signal.

APqrLE was implemented for the purpose of AP waveform enforcement and is identical to APqrPID, except that in APqrLE, V_m_ setpoint values (i.e., the model APs) are stored on the computer and used for the calculation of V_m_ error. In addition, blue light output (0.5 mW/cm^2^, 10-ms duration) is generated at 1 Hz to initiate APs by means of optogenetic pacing. When an AP upstroke is detected, correction is applied as specified for APqrPID.

### Quantification and statistical analysis

All measured variables, including AP properties and V_m_ error were determined by analyzing raw data using custom-written Python scripts uniformly applied to all experiments, thereby eliminating observer bias. Data are represented as mean ± standard deviation (SD), unless otherwise specified. Normal distribution of data was verified by the Shapiro-Wilk test, after which group means were statistically compared by two-way analysis of variance assuming sphericity, followed by Tukey`s *post-hoc* test for multiple comparisons by using GraphPad Prism 9 (GraphPad Software, LLC).

## Supplemental information

Document S1. Figures S1–S7

Document S2. Article plus supplemental information
