# Supplementary material for: Opto-electronic feedback control of membrane potential for real-time control of action potentials
Source: Cell Rep Methods. 2023 Dec 11;3(12):100671. doi: 10.1016/j.crmeth.2023.100671 (PMC10753386; doi:10.1016/j.crmeth.2023.100671)
Supplement: Document S1. Figures S1–S7 [file mmc1.pdf]

Cell Reports Methods, Volume *3*

Supplemental information

Opto-electronic feedback control of membrane potential for real-time control of action potentials

Balázs Ördög, Tim De Coster, Sven O. Dekker, Cindy I. Bart, Juan Zhang, Gerard J.J. Boink, Wilhelmina H. Bax, Shanliang Deng, Bram L. den Ouden, Antoine A.F. de Vries, and Daniël A. Pijnappels

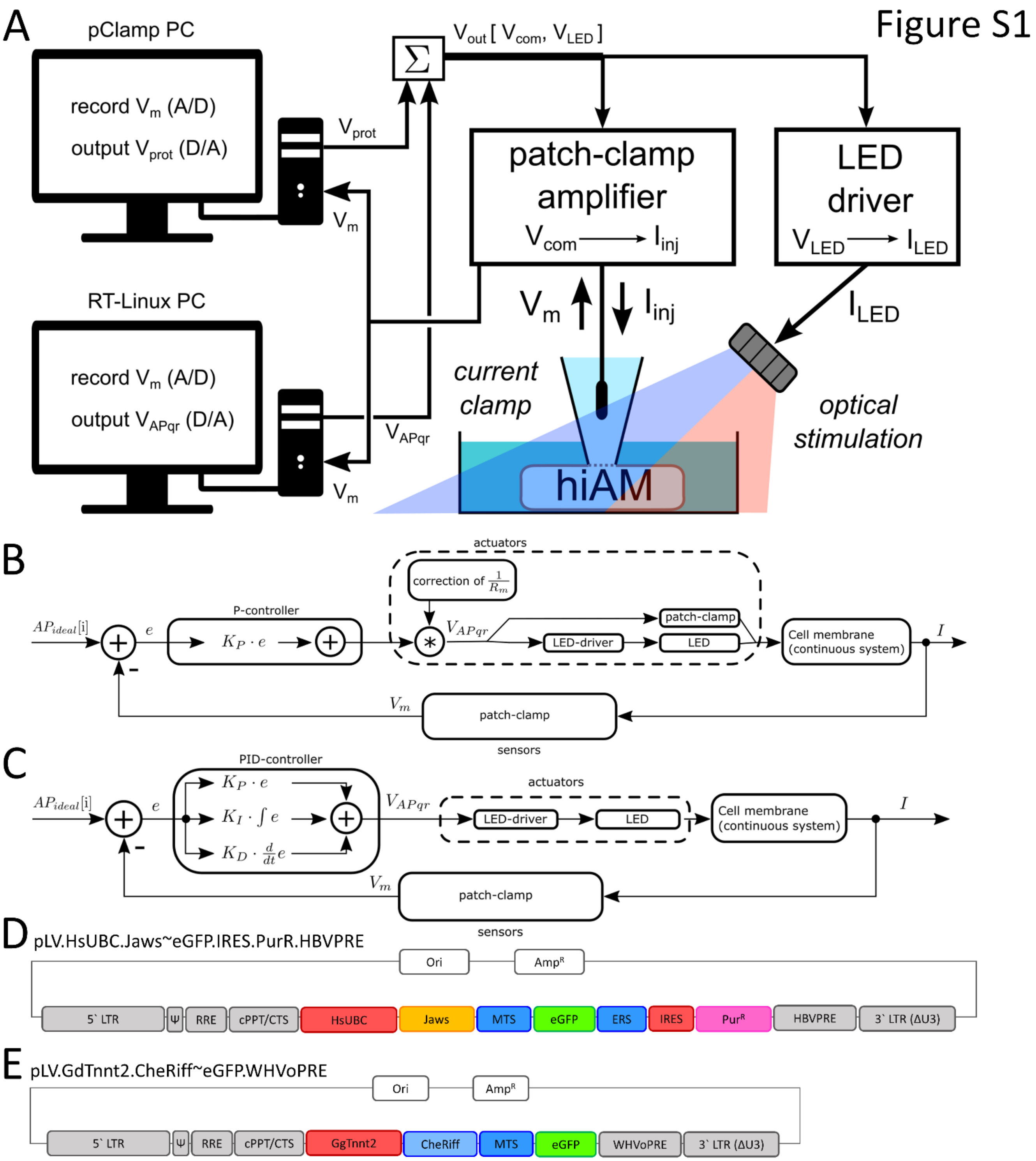

Figure S1 Schematics of the experimental setup, closed-loop controller algorithm and lentiviral vectors. Related to Figure 1 and STAR Methods. **(A)** Schematics of the experimental system and design. Real-time V_m data is obtained by the whole-cell mode of the patch clamp technique in single hiAMs or hiAM monolayers and is fed to two separate computers via analog-digital interfaces. A computer running the pClamp software package (pClamp PC) is used to record V_m and to create the preprogrammed analog output V_{prot}. The real-time computer (RT-Linux PC) also samples V_m and produces the dynamic analog output signal V_{APqr}. In our system, V_m can be targeted by direct current injection or by means of optogenetics. To generate electrical output, V_{prot} and V_{APqr} are summed by a custom-built summing amplifier (Σ) to yield V_{com} that serves as command input for the patch clamp amplifier to create I_{inj}. For optical output, V_{prot} and V_{APqr} may be summed or used separately in V_{LED} for the modulation of LED drivers generating I_{LED}. (**B**) Closed-loop controller used in single cell experiments. The output `I` was used either as command signal for the patch clamp amplifier to generate direct current injection or as control signal for the 617-nm LED driver. (**C**) Closed-loop controller used in dual-actuator optogenetic V_m control for AP restoration and for the enforcement of AP morphologies. **(D and E)** Schematic maps of lentiviral vector shuttle constructs used in the study for the over-expression of Jaws (D) and CheRiff (E). Indicated are viral vector elements (gray), including the human immunodeficiency virus type 1 5` long terminal repeat (LTR) containing the Rous sarcoma virus LTR promoter (5` LTR), the

U3 region-deleted 3` LTR (3` LTR [ΔU3]), packaging signal (Ψ), Rev-response element (RRE), central termination site and polypurine tract (cPPT/CTS) and the woodchuck hepatitis virus post-transcriptional regulatory element (WHVoPRE) or the human hepatitis B virus posttranscriptional regulatory element (HBVPRE), bacterial replication origin (Ori) and *Escherichia coli* β-lactamase gene (Amp^R). Jaws expression was driven by the human ubiquitin C promoter (HsUBC) and coupled with the expression of puromycin-N-acetyltransferase (PurR) via an internal ribosomal entry site (IRES). Both CheRiff and Jaws were C-terminally fused to the enhanced green fluorescent protein (eGFP). Jaws was equipped with the plasma membrane trafficking signal (MTS) and ER exit signal (ERS) of Kir2.1 [S1], whereas CheRiff was only equipped with the MTS of Kir2.1 [S2]. The expression of the CheRiff cassette was driven by the chicken troponin T promoter (GgTnnt2).

Figure S2

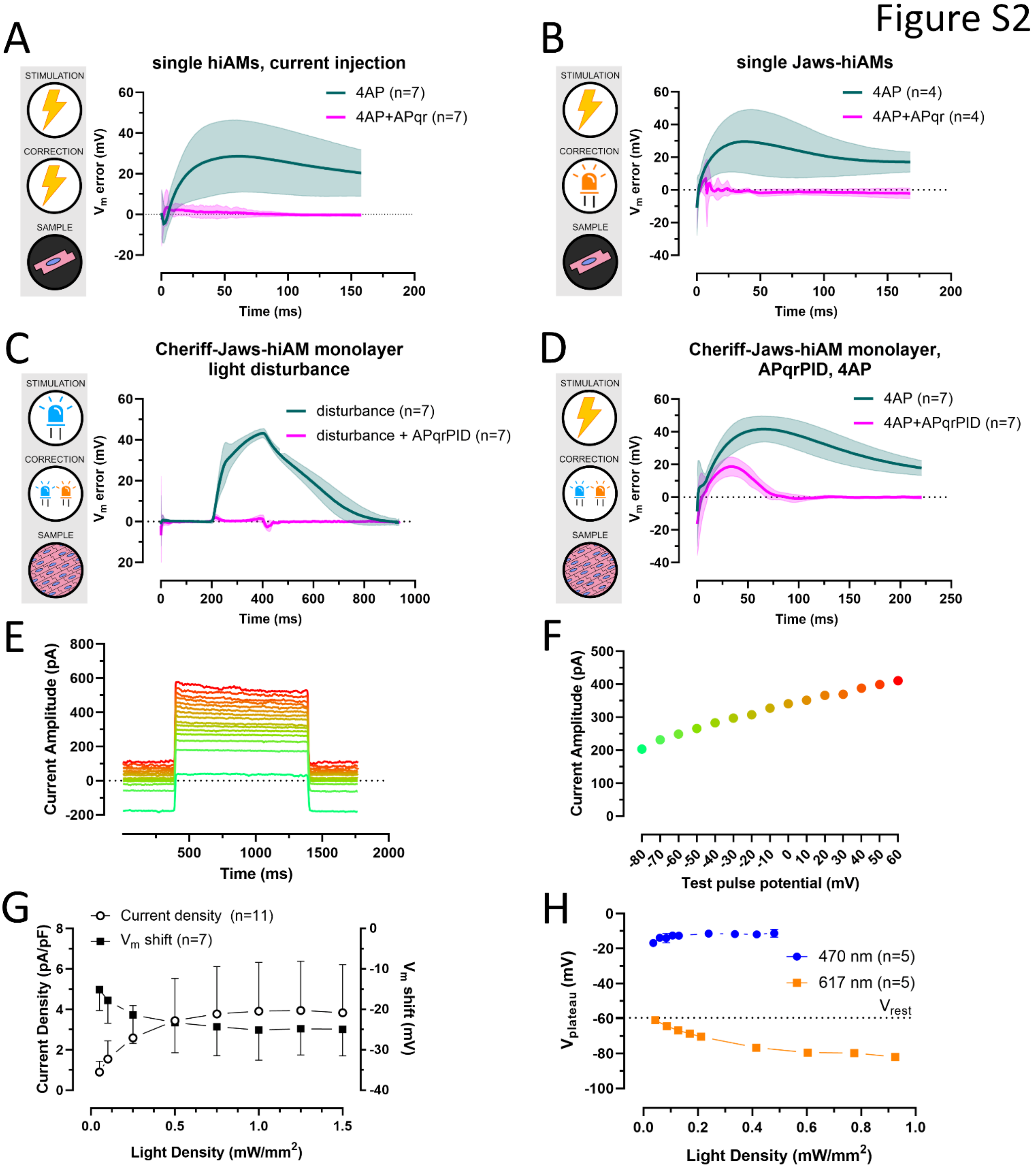

Figure S2 Extended data on V_m error during AP shape restoration and characterization of light response in optogenetically modified hiAM cells and monolayers. Related to Figures 2–7. (**A**) Average V_m error following 4AP administration (4AP) and during direct current injection-mediated AP restoration in the presence of 4AP (4AP+APqr) in n=7 single hiAMs. (**B**) Average V_m error following 4AP administration (4AP) and during light-mediated AP restoration in the presence of 4AP (4AP+APqr) in n=4 single hiAMs expressing Jaws. (**C**) Average V_m error in the presence of an optogenetically induced AP abnormality (disturbance) and during light-mediated AP restoration (dist.+APqrPID) in n=7 hiAM monolayers expressing CheRiff and Jaws. (**D**) Average V_m error following 4AP administration (4AP) and during light-mediated AP restoration (4AP+APqrPID) in n=7 hiAM monolayers expressing Cheriff and Jaws. In panels A to D, data are shown as mean ± SD, represented by continuous lines and shaded areas, respectively. A–D related to Figures 2–5. (**E**) Representative recording of Jaws current elicited by 565 nm light (1 s, 1.5 mW/mm^2) in a single hiAM from test potentials between -80 and 60 mV in 10 mV increments. (**F**) Current-voltage relationships obtained from the recording shown in panel E. (**G**) Whole cell current density (open circles, left axis) and V_m shift from V_{rest} (filled squares, right axis) observed at the end of 617-nm light pulses of 1 s and various intensities from 0.05 to 1.5 mW/mm^2 in single hiAMs expressing Jaws. E–G related to Figure 3. (**H**) $V_{plateau}$, representing V_m observed at the end of 470- and 617-nm light pulses of 1 s and various intensities from 0.04 to 0.48 mW/mm^2 and from 0.04 to 0.93 mW/mm^2, respectively, in hiAM monolayers expressing CheRiff and Jaws. Figure H related to Figures 4–7.

Figure S3

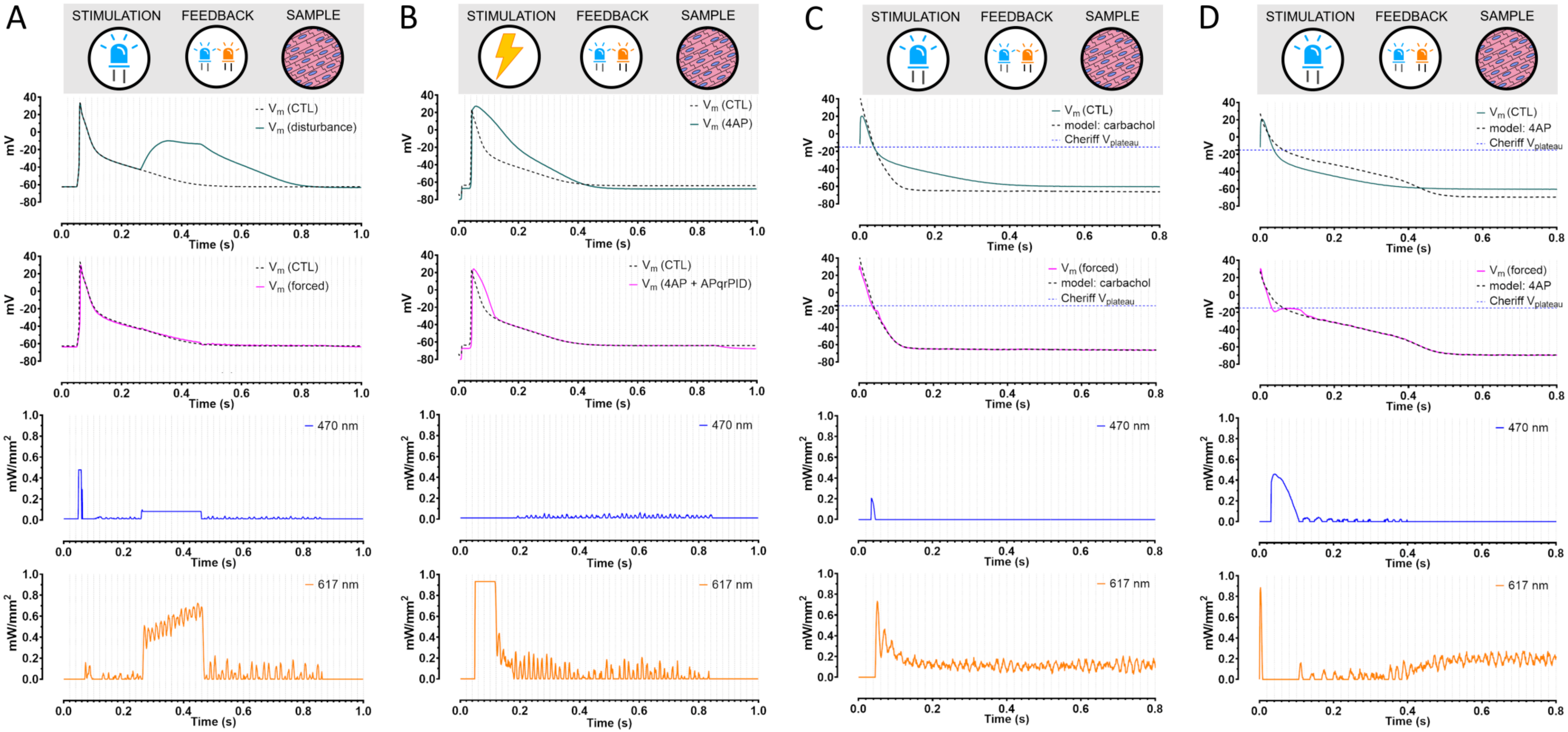

Figure S3 V_m and light output during AP shape restoration and enforcement experiments. Related to Figures 4, 5 and 6. (**A**) Representative recordings from the dual-actuator optogenetic AP restoration experiment in the context of a light-induced electrical disturbance (also shown in Figure 4C). (**B**) Representative recordings from the dual-actuator optogenetic AP restoration experiment in the context of a drug-induced electrical disturbance (also shown in Figure 5C). (**C**) Representative recordings from the dual-actuator optogenetic AP morphology enforcement of the ‘carbachol’ model APs (also shown in Figure 6B). (**D**) Representative recordings from the dual-actuator optogenetic AP morphology enforcement of the ‘4AP’ model APs (also shown in Figure 6C).

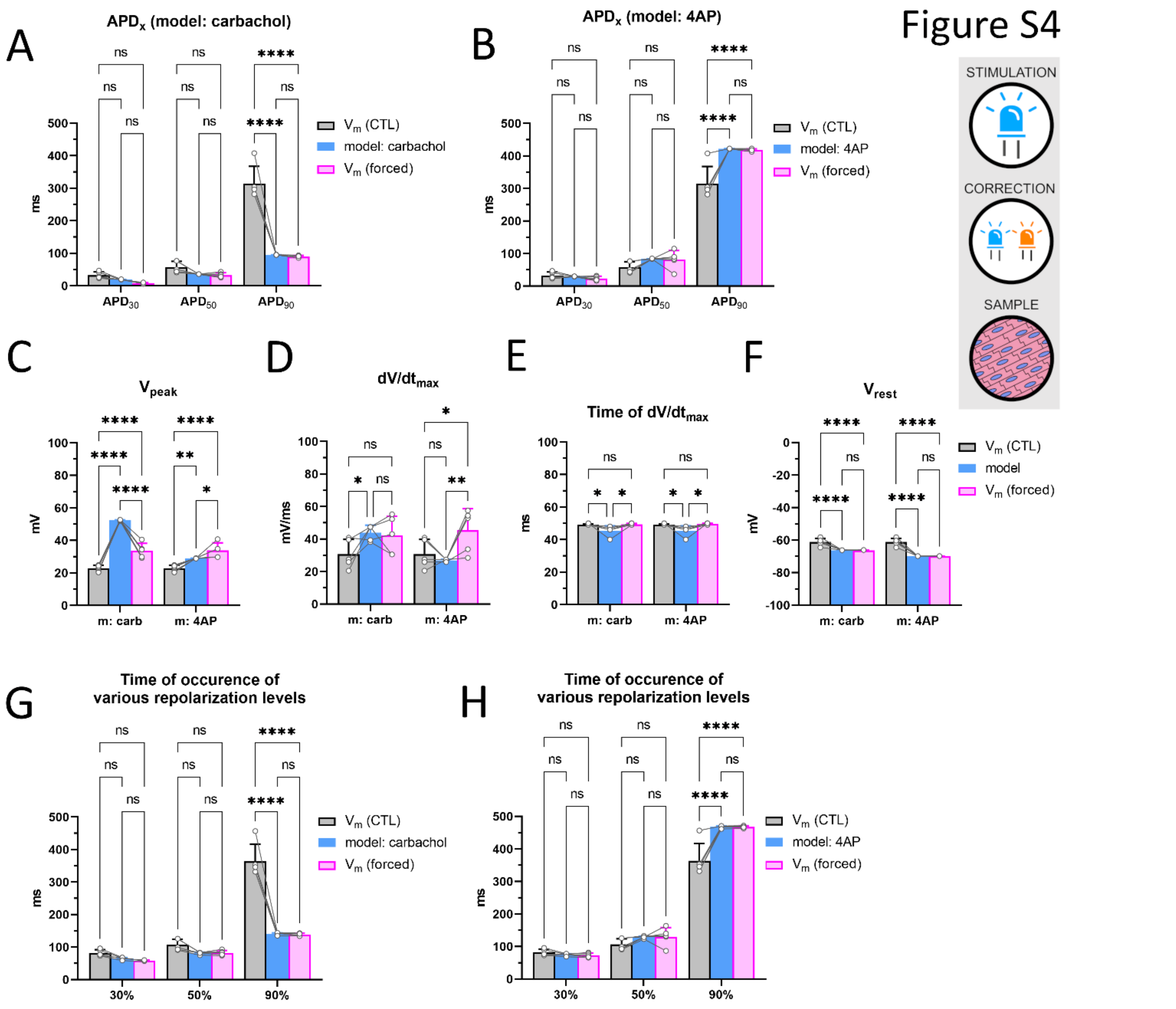

Figure S4 Extended data on AP parameters during enforcement of drug-induced AP abnormality models. Related to Figure 6. (**A and B**) Mean APD values ± SD measured at 20, 50 and 90% repolarization. (**C**) V_{peak} representing the most positive V_m value during an AP. (**D**) dV/dt_{max} representing maximum upstroke velocity. (**E**) Time to reach dV/dt_{max} from an arbitrarily chosen time point preceding the AP. (**F**) Resting potential. (**G** and **H**) The moment when 30%, 50% or 90% repolarization is reached during enforcement of the carbachol and the 4AP models. Data are shown as mean ± SD. *: $P < 0.05$, **: $P < 0.01$, ****: $P < 0.0001$, ns: not significant.

Figure S5

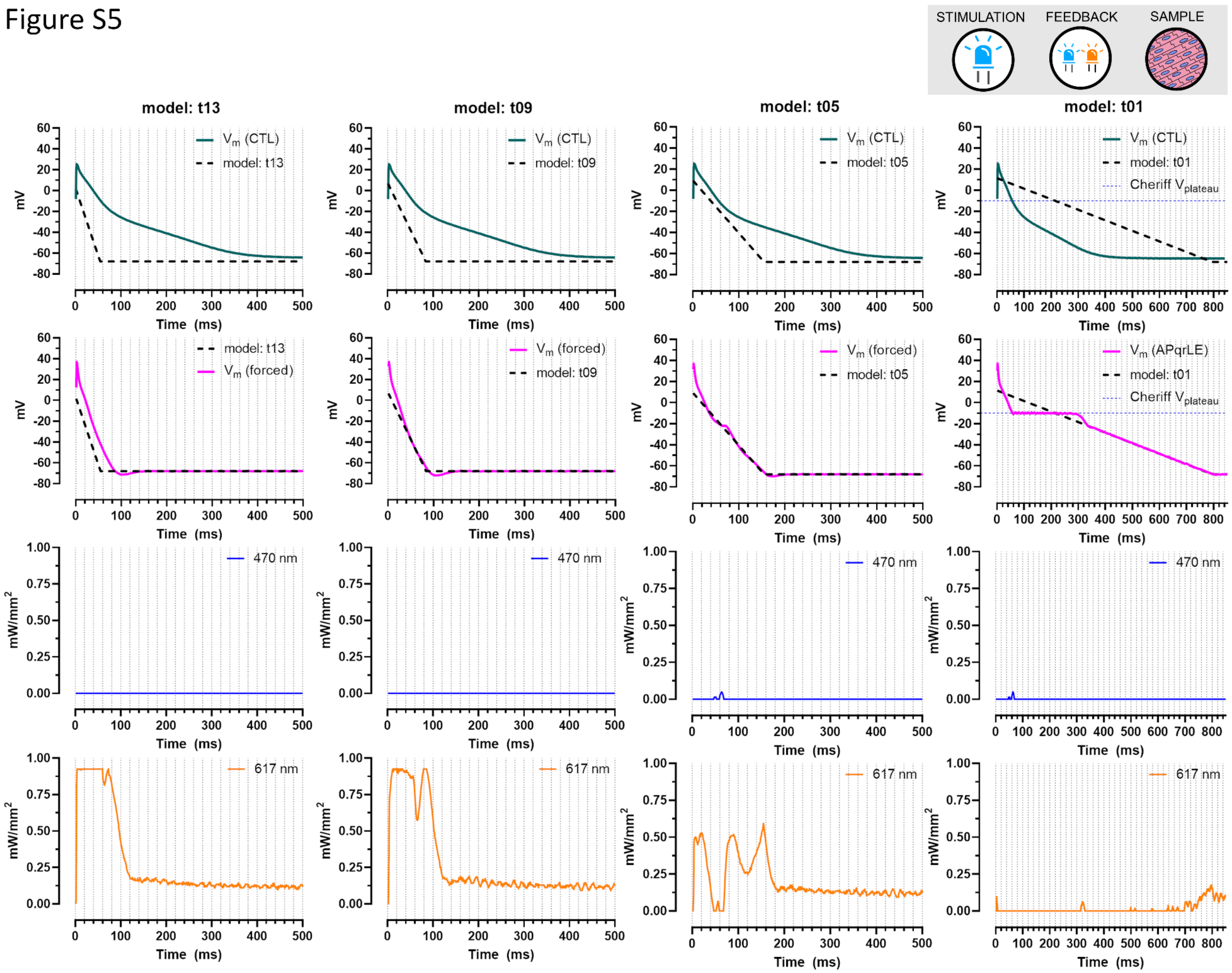

Figure S5 Representative recordings from the dual-actuator optogenetic AP morphology enforcement of the ‘t13’, ‘t09’, ‘t05’ and ‘t01’ model APs. Related to Figure 7.

Figure S6

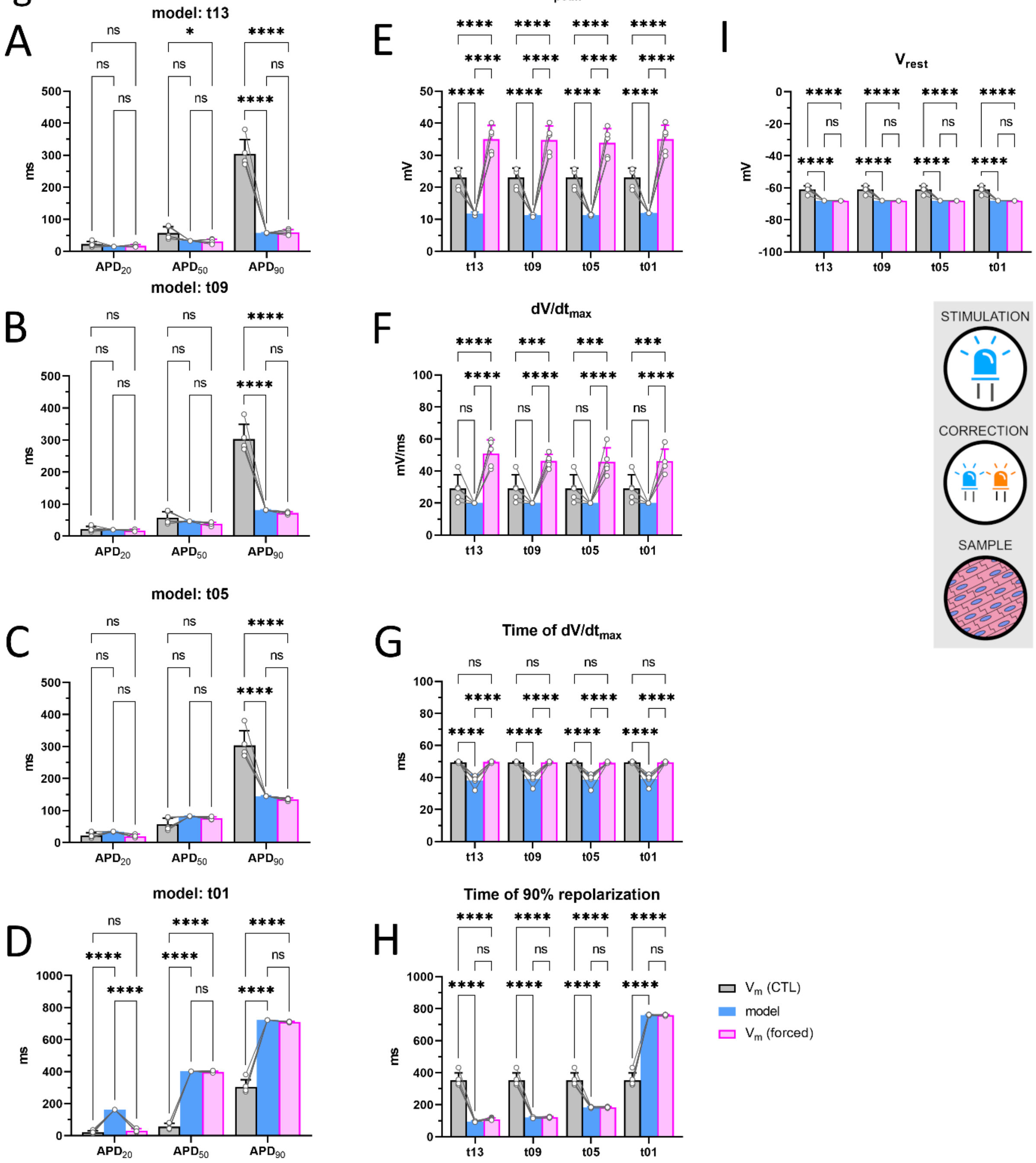

Figure S6 Extended data on enforcement of simplified AP models. The endogenous APs (CTL) were compared to model (model) and enforced (forced) APs. Related to Figure 7. (**A**–**D**) APD values measured at 20, 50 and 90% repolarization for the models t13, t09, t05 and t01, respectively. (**E**) V_{peak} representing the most positive V_m value during an AP. (**F**) dV/dt_{max} representing maximum upstroke velocity. (**G**) Time to reach dV/dt_{max} from an arbitrarily chosen time point preceding the AP. (**H**) The moment when 90% repolarization is reached. (**I**) Resting potential. Data are shown as mean ± SD. *: P < 0.05, ***: P < 0.001, ****: P < 0.0001, ns: not significant. Column labels for each panel are as shown in panel **H**.

Figure S7

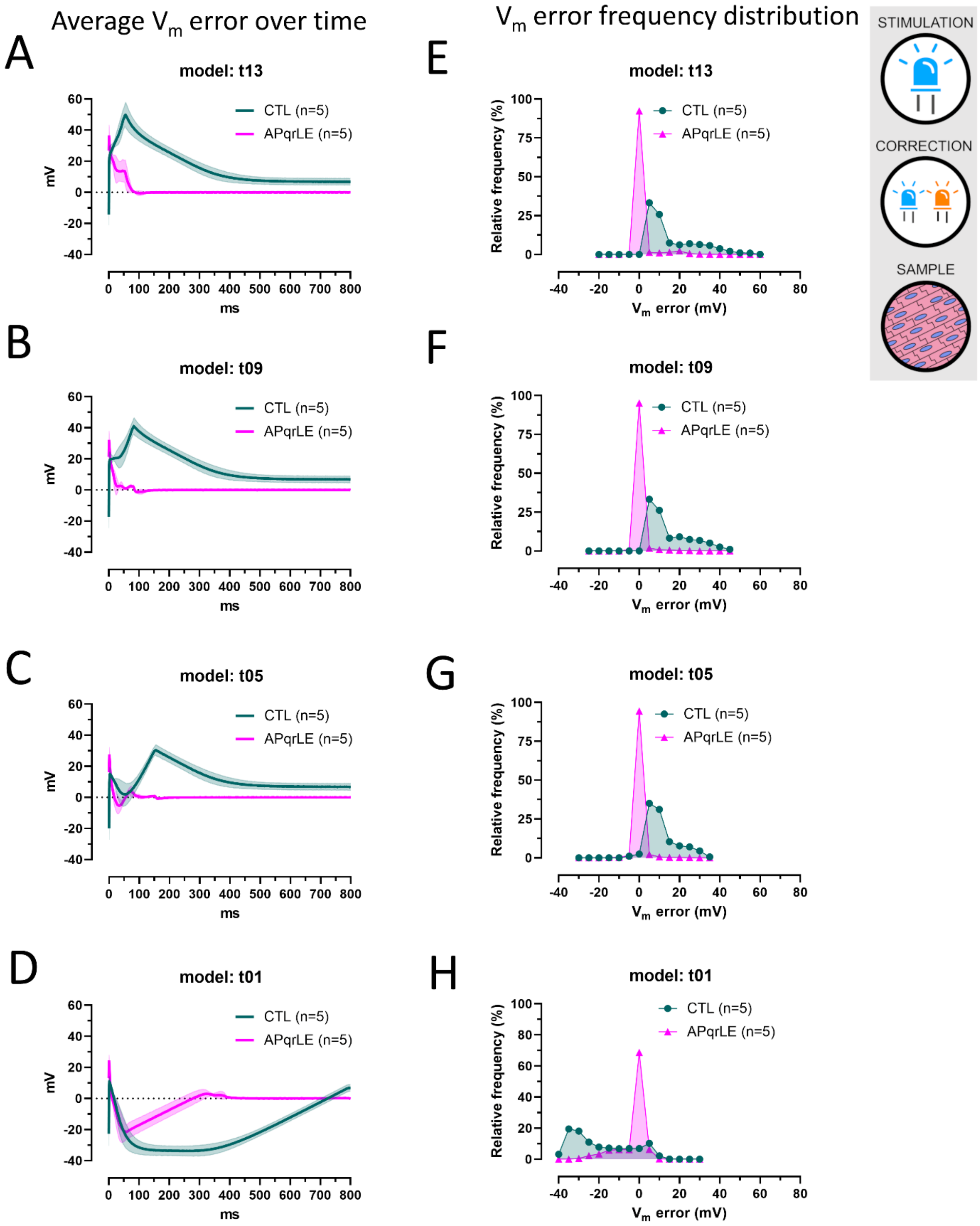

Figure S7 Extended data on enforcement of simplified AP models. Related to Figure 7. (**A**-**D**) V_m error representing the difference over time between model vs endogenous APs (CTL) and model vs enforced APs (APqrLE) for AP models t13, t09, t05 and t01, respectively. Data are shown as mean (continuous lines) ± SD (shaded areas). (**E**-**H**) Frequency distribution of V_m error for n=5 monolayers with a 5-mV bin width for AP models t13, t09, t05 and t01, respectively.

References

S1. Chuong, A.S., Miri, M.L., Busskamp, V., Matthews, G.A., Acker, L.C., Sorensen, A.T., Young, A., Klapoetke, N.C., Henninger, M.A., Kodandaramaiah, S.B., et al. (2014). Noninvasive optical inhibition with a red-shifted microbial rhodopsin. Nat Neurosci 17, 1123-1129. 10.1038/nn.3752.

S2. Hochbaum, D.R., Zhao, Y., Farhi, S.L., Klapoetke, N., Werley, C.A., Kapoor, V., Zou, P., Kralj, J.M., Maclaurin, D., Smedemark-Margulies, N., et al. (2014). All-optical electrophysiology in mammalian neurons using engineered microbial rhodopsins. Nat Methods 11, 825-833. 10.1038/nmeth.3000.
