# Supplementary material for: Opto-electronic feedback control of membrane potential for real-time control of action potentials
Source: Cell Rep Methods. 2023 Dec 11;3(12):100671. doi: 10.1016/j.crmeth.2023.100671 (PMC10753386; doi:10.1016/j.crmeth.2023.100671)
Supplement: Document S2. Article plus supplemental information [file mmc2.pdf]

Cell Reports Methods

Article

Opto-electronic feedback control of membrane potential for real-time control of action potentials

Graphical abstract

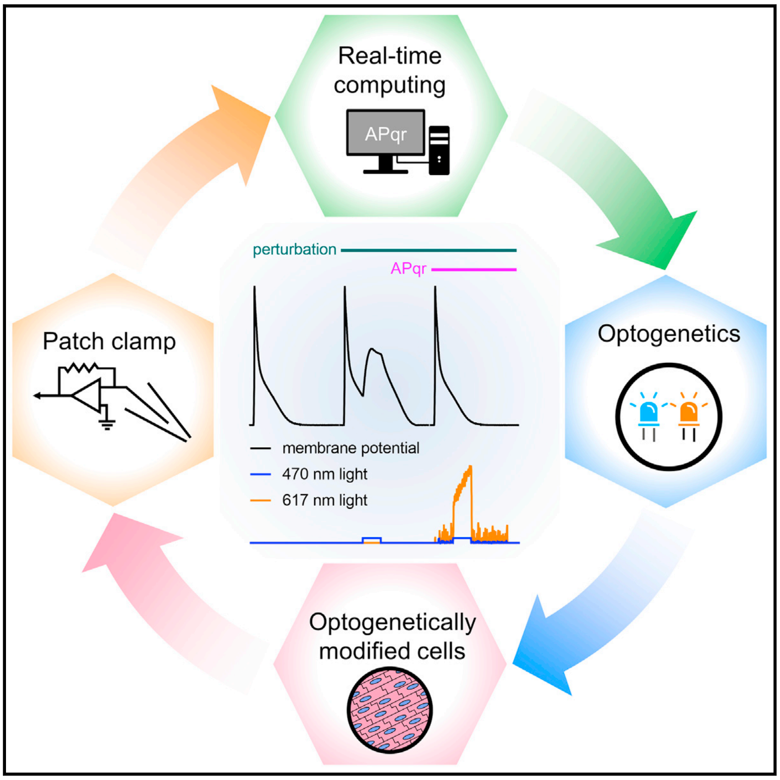

Highlights

- APqr integrates cellular electrophysiology, real-time computing, and optogenetics
- APqr preserves cardiac AP morphologies in the presence of electrical perturbations
- APqr allows the enforcement of arbitrary AP waveforms
- APqr sets the stage for tissue-scale membrane potential control

Authors

Balázs Ördög, Tim De Coster, Sven O. Dekker, ..., Bram L. den Ouden, Antoine A.F. de Vries, Daniël A. Pijnappels

Correspondence

b.ordog@lumc.nl (B.Ö.), d.a.pijnappels@lumc.nl (D.A.P.)

In brief

Ördög et al. establish APqr, a closed-loop experimental system for the dynamic control of the membrane potential in cardiomyocytes. Their approach sets the stage for the refinement and application of opto-electronic control systems to enable in-depth investigation into regulatory roles of the membrane potential in health and disease.

Ördög et al., 2023, Cell Reports Methods *3*, 100671
December 18, 2023 © 2023 The Authors.
https://doi.org/10.1016/j.crmeth.2023.100671

CellPress

Cell Reports Methods

CellPress
OPEN ACCESS

Article

Opto-electronic feedback control of membrane potential for real-time control of action potentials

Balázs Ördög,[1,5,*] Tim De Coster,[1,5] Sven O. Dekker,[1] Cindy I. Bart,[1] Juan Zhang,[1] Gerard J.J. Boink,[2,3] Wilhelmina H. Bax,[1] Shanliang Deng,[1,4] Bram L. den Ouden,[1,4] Antoine A.F. de Vries,[1,5] and Daniël A. Pijnappels[1,5,6,*]

[1]Laboratory of Experimental Cardiology, Department of Cardiology, Heart Lung Center Leiden, Leiden University Medical Center, 2333 ZA Leiden, the Netherlands

[2]Amsterdam Cardiovascular Sciences, Department of Cardiology, Amsterdam UMC, Location AMC, University of Amsterdam, 1105 AZ Amsterdam, the Netherlands

[3]Department of Medical Biology, Amsterdam UMC, Location AMC, University of Amsterdam, 1105 AZ Amsterdam, the Netherlands

[4]Department of Microelectronics, Delft University of Technology, 2628 CD Delft, the Netherlands

[5]These authors contributed equally

[6]Lead contact

*Correspondence: b.ordog@lumc.nl (B.Ö.), d.a.pijnappels@lumc.nl (D.A.P.)

https://doi.org/10.1016/j.crmeth.2023.100671

MOTIVATION In excitable cells, the membrane potential undergoes transient, periodic changes called action potentials (APs). Organ-level function, such as heart contraction, depends on distinct local AP morphologies, whereas AP abnormalities can give rise to disorders, such as cardiac arrhythmias. APs are highly dynamic, multifactorial processes and have stochastic characteristics. To gain a deeper understanding into the regulatory roles of APs in health and in the development and/or progression of disease, experimental tools that are able to control APs in a precise and similarly dynamic manner are needed. Yet, methods capable of such control are currently lacking.

SUMMARY

To unlock new research possibilities by acquiring control of action potential (AP) morphologies in excitable cells, we developed an opto-electronic feedback loop-based system integrating cellular electrophysiology, real-time computing, and optogenetic approaches and applied it to monolayers of heart muscle cells. This allowed accurate restoration and preservation of cardiac AP morphologies in the presence of electrical perturbations of different origin in an unsupervised, self-regulatory manner, without any prior knowledge of the disturbance. Moreover, arbitrary AP waveforms could be enforced onto these cells. Collectively, these results set the stage for the refinement and application of opto-electronic control systems to enable in-depth investigation into the regulatory role of membrane potential in health and disease.

INTRODUCTION

All living cells maintain a membrane potential (V_m). V_m is determined by the distribution of ions between the extra- and intracellular space established by membrane-resident ion channels, exchangers, and pumps.[1] In excitable cells, V_m undergoes fast and transient changes called action potentials (APs), which may propagate unidirectionally within the cell membrane and between electrically coupled cells. In the nervous system and the heart, organ-level functions depend on finely tuned and often distinctly different local AP morphologies.[2,3] Furthermore, subtle abnormalities of local AP properties may lead to organ dysfunction.[4] However, due to the limitations of methods available for V_m control and in particular for the control of AP morphologies, the exact roles of AP characteristics in health and disease and the potential benefits of AP abnormality correction remain incompletely understood.

V_m can be controlled by the so-called voltage clamp modality of the patch-clamp technique, where V_m is clamped to predetermined values, and the concurrent transmembrane currents are measured. Interestingly, V_m can also be clamped to a series of voltages that collectively give rise to the shape of an AP. These voltages can either be set prior to the experiment in the so-called AP clamp configuration or simulated real-time in dynamic AP clamp experiments.[5–9] A common feature of all types of voltage clamp, however, is that the desired V_m value is imposed on the cell by

This is an open access article under the CC BY license (http://creativecommons.org/licenses/by/4.0/).

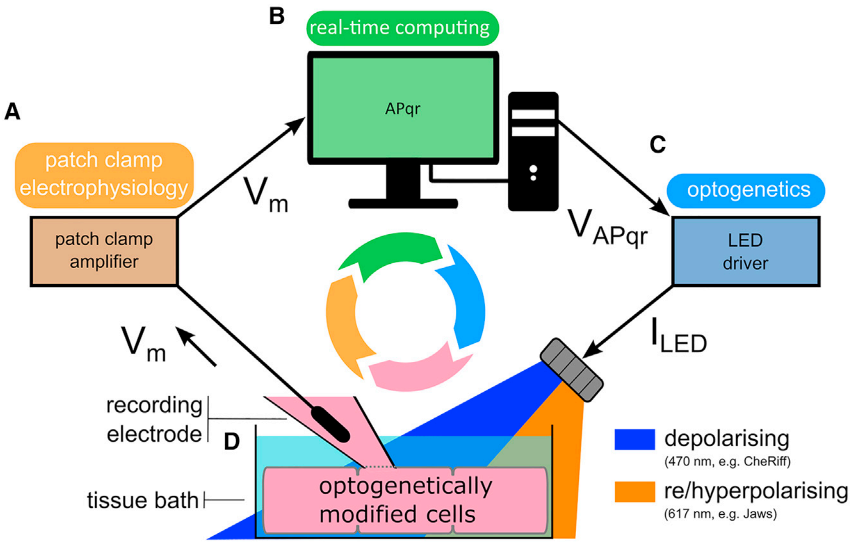

Figure 1. Schematic illustration of the main components of the experimental system used in this study

(A) Real-time, accurate V_m measurement is obtained by patch-clamp electrophysiology in hiAMs in single-cell or monolayer formats.

(B) V_m data fed to the custom closed-loop control algorithm (APqr) hosted on a real-time computing platform generating the dynamic output V_{APqr}.

(C and D) Optogenetic approaches are used to interface real-time computing and the biological component, consisting of LED light sources controlled by V_{APqr} (C) and optogenetic actuators expressed in hiAMs (D). See also Figure S1A.

direct current injection via the recording electrode. This limits the applicability of voltage clamp in the multicellular setting, especially in tissue with low net impedance, such as cardiac muscle tissue.

The transmembrane currents needed to control V_m, however, may be generated independently of the tissue‘s impedance by means of optogenetics. Optogenetics is a highly versatile and rapidly evolving research methodology that employs light-sensitive proteins to observe and/or interfere with cell function. For example, exploiting their ability to depolarize the cell membrane upon illumination, light-gated cation channels known as channelrhodopsins (ChRs)[10,11] have been used in a wide range of applications, including cardiac pacing,[12,13] resynchronization of heart segments,[14] termination of cardiac arrhythmias,[15–17] and for the optical manipulation of excitation waves both in monolayers of cardiomyocytes[18,19] and in the intact heart.[20] Furthermore, optogenetics has been used as a direct, non-invasive replacement of current injection for different purposes. Bidirectional V_m control was achieved by the selective activation of ChR2 and an outward-current-generating opsin (i.e., ArchT or eNpHR3.0) in order to clamp the activity of neurons to specific firing rates[21] and to create the voltage steps necessary to activate ectopically expressed ion channels for drug profiling purposes.[22] ArchT has also been used for the supplementation of induced pluripotent stem cell-derived cardiomyocytes (iPSC-CMs) with the I_{K1} current in an optical dynamic clamp arrangement, thereby improving AP characteristics of these cells.[23] More recently, an all-optical voltage clamp platform was applied to control neural activity by the combined use of BIPOLES, a fusion between a cation- and an anion-selective ChR shunting V_m to specific voltages in a wavelength-dependent manner, and of a fluorescent V_m readout in a closed-loop arrangement.[24]

Previous studies already demonstrated the ability to optogenetically modulate AP waveforms. A computational study explored the feasibility of optogenetic prolongation of action potential durations (APDs) in a three-dimensional model of human atria with pathological APD shortening.[25] In the experimental setting, Park et al. showed light-induced prolongation and shortening of APD in neonatal rat ventricular myocytes via activation of ChR2 and eNpHR3.0, respectively.[26] Using the same cell type, Govorunova et al. achieved APD shortening in a light duration- and intensity-dependent manner by activation of the anion ChR GtACR1.[27] Furthermore, Gruber et al. optogenetically corrected abnormal APDs in patient-derived iPSC-CMs in the context of long and short QT syndromes.[28] In all former studies aiming at the control and/or correction of AP waveforms, the characteristics of the modulatory light pulses (e.g., timing, intensity, and duration) and of the endogenous APs needed to be determined before the intervention. While such deterministic approach is robust and effective, it needs to be tailored to every combination of cell type and pathology (e.g., short or long QT syndrome-causing mutations).

Here, we now show that this customization is no longer necessary by employing closed-loop control principles, an inherently adaptive systems' control scheme widely used in physiological regulatory mechanisms.[29] Our system, herein referred to as APqr, uses real-time V_m data obtained by patch-clamp electrophysiology (Figure 1A) as the only input without any prior calibration. V_m data are fed to a custom feedback loop controller algorithm hosted on a real-time computing platform creating the dynamic output V_{APqr} (Figure 1B). Finally, optogenetics is used to interface real-time computing and the biological components (Figure 1C), as explained in detail in the STAR Methods and the following sections. The blue light-gated cation channel CheRiff[30] and the red light-activatable inward chloride pump Jaws[31] are used as effectors for direct targeting of V_m in conditionally immortalized human atrial myocytes (hiAMs, Figure 1D).[32] By using APqr, AP morphologies perturbed either by light or by drug exposure can be restored to their normal states in a dynamic, self-regulatory manner in multicellular hiAM clusters without any prior information on the disturbance. In addition, arbitrarily chosen AP waveforms can be enforced onto cardiomyocytes with physiologically relevant accuracy and precision.

RESULTS

AP shape restoration

Since abnormal AP waveforms can have detrimental consequences, their restoration to a normal state may be beneficial.

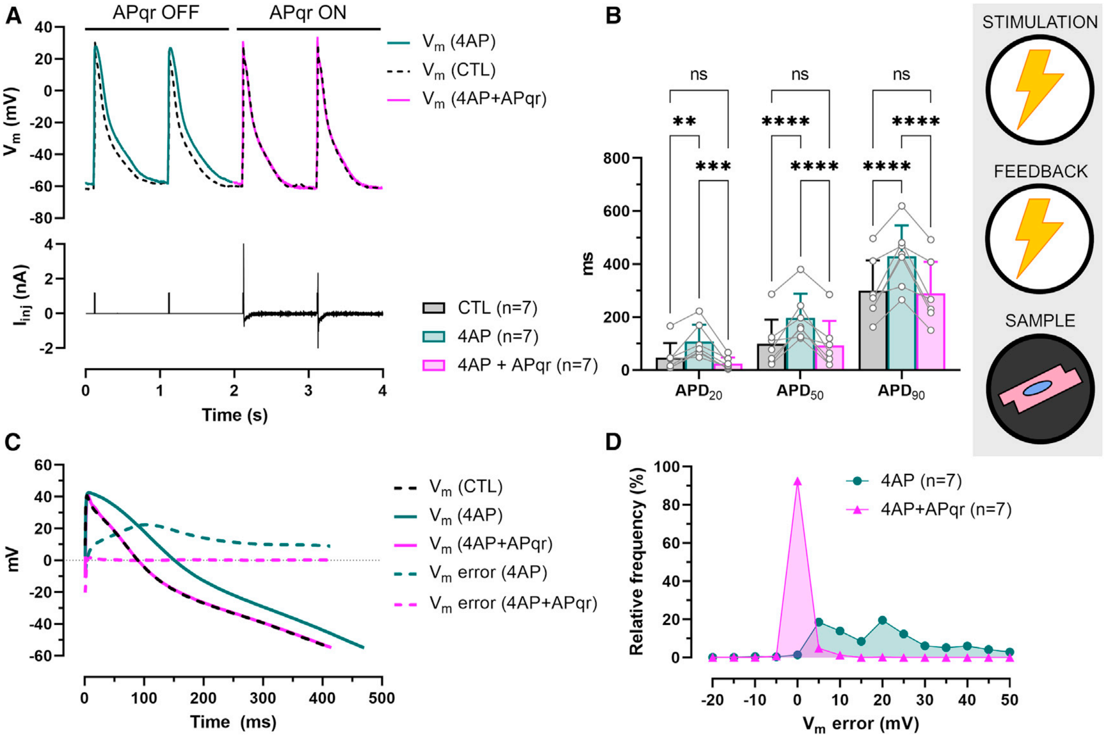

Figure 2. AP shape restoration by direct current injection in single cells

(A) Representative recordings of electrically triggered APs (upper panel) under normal conditions (CTL), following 4AP administration (4AP), and during APqr intervention in the presence of 4AP (4AP + APqr), and of the injected current (lower panel) in a single hiAM.

(B) Mean APD values ±SD measured at 20%, 50%, and 90% repolarization in n = 7 single hiAMs. **$p < 0.005$, ***$p < 0.001$, ****$p < 0.0001$, ns: not significant.

(C) Representative recordings of APs shown between the moment of dV/dt_{max} and the moment of APD_{90}, overlaid with V_m error calculated for the drug-affected (4AP) and restored (4AP + APqr) APs compared to CTL.

(D) Frequency distribution of V_m error in n = 7 single hiAMs before (4AP) and during AP shape restoration (4AP + APqr) with a 5-mV bin width. See also Figure S2A.

To achieve this, APqr was designed to accomplish the following functionalities. When activated, APqr first logs a chosen number of APs, averages them across time, and stores the average AP as V_m setpoint values. Following the logging phase, APqr calculates the difference between the measured and setpoint V_m value as V_m error and generates an output (V_{APqr}, Figures 1 and S1) seeking to minimize V_m error. Since V_{APqr} is proportional to V_m error and is adjusted after evaluating a set of conditions (e.g., overshoot or increasing V_m error), the control feedback loop is fully adaptive. These calculations are carried out at fixed time steps: once per millisecond in all our experiments. AP abnormalities were induced either by means of a preprogrammed blue light pulse activating CheRiff or by non-selective potassium channel blockade (4-aminopyridine [4AP], 200 μM). In experiments with 4AP, APqr was paused after the logging phase to allow 4AP effects to develop. When 4AP effects became evident, APqr was resumed and left running without any further input from the experimenter.

AP shape restoration by direct current injection in single cells

APqr was first used to target V_m by direct current injection via the recording patch-clamp electrode in single hiAMs. In this arrangement, V_{APqr} served as control signal for the patch-clamp amplifier (V_{com}) producing the injected current (I_{inj}) proportional to V_{APqr} (Figure S1A). Electrical perturbance was created by 4AP administration. 4AP is expected to slow down the repolarization phase of the AP (return from AP peak to resting potential). In accordance with this, APs indeed showed a broadened morphology in the presence of 4AP when APqr was paused (APqr OFF, Figure 2A). However, after activation of APqr, normal AP morphologies were maintained (APqr ON, Figure 2A). The effects of 4AP and the APqr intervention were reflected in APD values calculated as the interval between the moment of maximum upstroke velocity (dV/dt_{max}) and the moment at which various (20%, 50%, and 90%) repolarization levels were reached (Figure 2B). Administration of 4AP prolonged the APD as reflected by significantly increased average APD_{20}, APD_{50}, and APD_{90} values compared to control conditions (CTL, n = 7, $p < 0.005$). With APqr ON (4AP + APqr), however, average APDs were similar to CTL ($p > 0.05$, Figure 2B). For example, while APD_{90} values increased from 299.3 ± 115.3 ms in CTL to 429.9 ± 115.6 ms with 4AP, APqr reduced average APD_{90} to 289.8 ± 118.5 ms in the 4AP + APqr group, thereby reducing the 140.1-ms 4AP-induced APD_{90} difference to 9.5 ms on average. The efficacy of AP restoration could also be tested by calculating V_m error *post hoc* as the difference of V_m over time for the 4AP-affected (4AP) and restored (4AP + APqr) APs as compared to the reference V_m (i.e., the average CTL AP) during

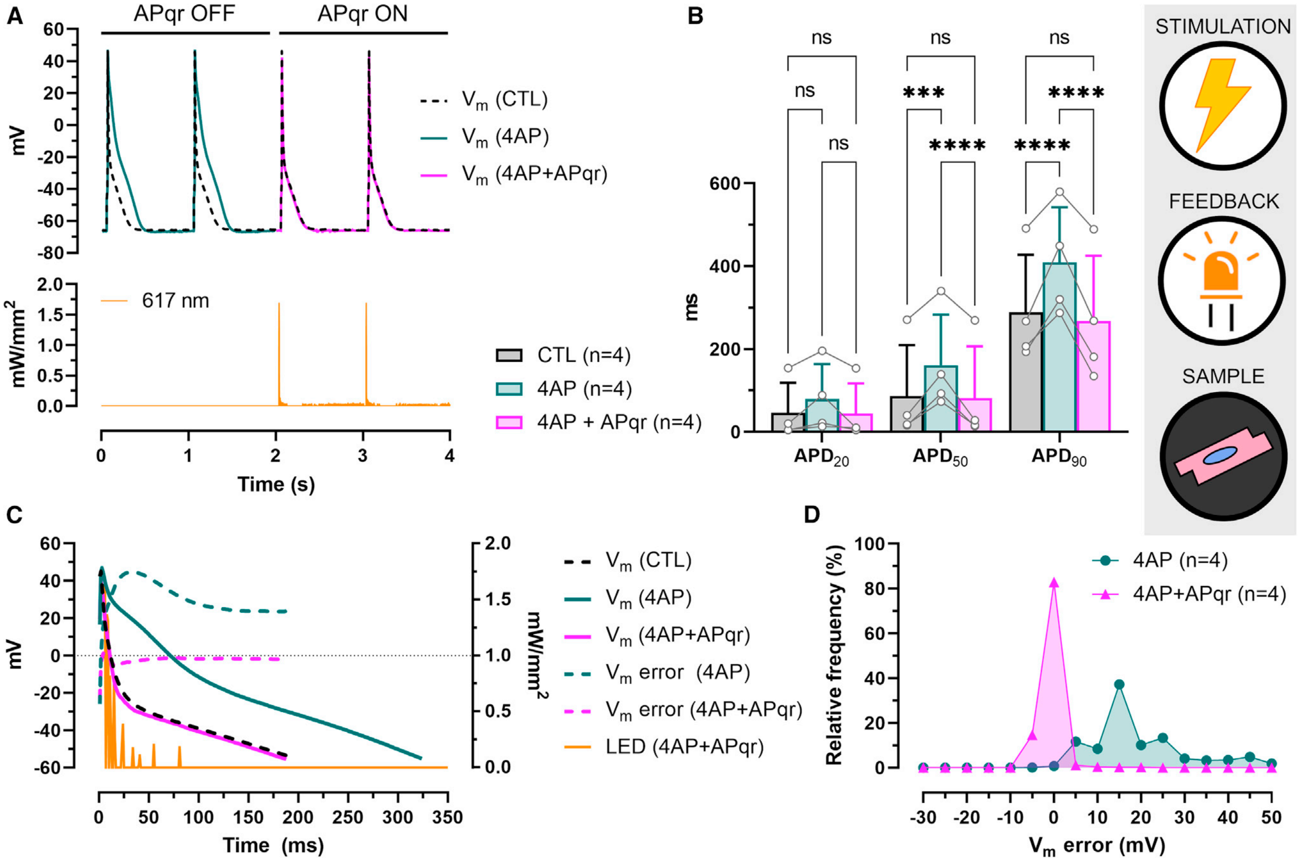

Figure 3. AP shape restoration by single-actuator optogenetics in single cells

(A) Representative recordings of electrically triggered APs (upper panel) in a single hiAM expressing Jaws, under normal conditions (CTL), following 4AP administration (4AP), and during APqr intervention in the presence of 4AP (4AP + APqr), and of the output of the 617-nm LED (lower panel).

(B) Mean APD values ±SD measured at 20%, 50%, and 90% repolarization in n = 4 single hiAMs. ***$p < 0.001$, ****$p < 0.0001$, ns: not significant.

(C) Representative recordings of APs shown between the moment of dV/dt_{max} and the moment of APD_{90}, overlaid with V_m error calculated for the drug-affected (4AP) and restored (4AP + APqr) APs compared to CTL. Also shown is the 617-nm light output during AP shape restoration.

(D) Frequency distribution of V_m error in n = 4 single hiAMs before (4AP) and during AP shape restoration (4AP + APqr) with a 5-mV bin width. See also Figures S2B and S2E–S2G.

the time course of an AP (between the moment of dV/dt_{max} and the moment of APD_{90}). V_m error was positive during most of the AP in the 4AP group, with a peak of 28.6 ± 17.7 mV at 62 ms (Figures 2C and S2A), a median value of 20 mV, and V_m error distributing between −2.5 and 2.5 mV only 1.4% of the time (Figure 2D). With APqr ON, 92.5% of the V_m error data points fell in the range of −2.5 to 2.5 mV, and the median was reduced to −0.02 mV (Figure 2D).

AP shape restoration by single-actuator optogenetics in single cells

In order to overcome the limitations of the direct current injection approach, we next tested the feasibility of closed-loop-controlled AP restoration by using optogenetics for V_m control. To this end, V_{APqr} was used to modulate light output of a 617-nm light-emitting diode (LED) via V_{LED} (Figures 1C and S1A). In these experiments, single hiAMs were genetically modified by lentiviral gene delivery to express Jaws (Figure S1D). As a light-activatable inward chloride pump, Jaws uses photonic energy to create an inward flux of chloride ions, thereby moving V_m in the negative direction. Illumination of Jaws-hiAMs via the microscope objective resulted in a V_m shift of up to −25 mV from the resting potential in a largely voltage-independent but light intensity-dependent manner (Figures S2E–S2G). 4AP induced a marked widening of AP shapes, which was reversed by APqr (Figure 3A). In more detail, APD_{50} and APD_{90} values were significantly prolonged in response to 4AP treatment ($p < 0.005$, n = 4), while no statistically significant difference was found in APD values in 4AP-treated cells subjected to APqr (4AP + APqr) as compared to CTL ($p > 0.05$, n = 4, Figure 3B). Average APD_{90} was 289.5 ± 138.1 ms in control cells, 408.7 ± 133.4 ms in 4AP-treated cells without APqr, and 268.3 ± 157.2 ms in 4AP-treated cells with APqr. As illustrated by the representative recordings shown in Figure 3C, APqr drastically reduced the marked positive V_m error for 4AP-affected APs for most of the APD (Figures 3C and S2B). Notably, light output was highest during the early repolarization phase following the AP peak. Without APqr correction, 98.6% of V_m error values obtained from n = 4 cells were larger than 2.5 mV with a median of 15.7 mV. The

APqr intervention, however, resulted in V_m error distributing around zero (0 ± 2.5) mV 83% of the time with a median value of −0.2 mV (Figure 3D).

AP shape restoration by dual-actuator optogenetics in multicellular preparations

To realize bidirectional V_m control, hiAMs were genetically modified to express both CheRiff and Jaws simultaneously by lentiviral gene transfer (Figures S1D and S1E). Once activated, the robust cation conductance created by CheRiff shunts V_m toward the reversal potential of the CheRiff current, thereby depolarizing the cell. CheRiff and Jaws could be selectively activated using light of 470 and 617 nm, resulting in depolarizing and re- or hyperpolarizing effects, respectively. In monolayers of CheRiff- and Jaws-expressing hiAMs, maximum intensity illumination (1.5 mW/mm^2) by the 470-nm and 617-nm LEDs resulted in −11.2 mV and −82 mV plateau potentials on average, as observed at the end of a 1-s light pulse, respectively ($V_{plateau}$, Figure S2H).

To implement dual-actuator closed-loop control, the APqr algorithm was modified to operate on two separate output channels. This control algorithm was dubbed APqrPID and developed based on the systems control principle known as proportional-integral-derivative (PID) controller.[33] Similarly to APqr, APqrPID logs and stores V_m data consisting of an average control AP as a series of setpoint values. V_{APqr} is computed online during the correction phase and is used to modulate either of the two LEDs (Figures 1C and S1A).

AP restoration in the presence of a light-induced electrical disturbance in multicellular preparations

APqrPID was first used to neutralize light-induced AP abnormalities in CheRiff- and Jaws-expressing hiAMs. In these experiments, every 5th AP was perturbed by a 200-ms light pulse (470 nm, 0.1 mW/mm^2) given 200 ms after the pacing stimulus (1 Hz, 10 ms, 470 nm, 0.5 mW/mm^2) to create abnormal cation influx during hiAM repolarization. This allowed the assessment of AP restoration efficacy in the presence of a sudden (i.e., unexpected) electrical perturbance. The representative recordings in Figure 4A illustrate that the preprogrammed extra light pulse induced a marked deflection of V_m resulting in an abnormal AP shape. APqrPID prevented this deflection by the autonomous (i.e., not predetermined) activation of the 617-nm LED output and restored AP morphology to normal (also shown on separate axes in Figure S3A). This was reflected in APD_{90} values, which increased from 342.9 ± 51.7 ms in CTL to 700.8 ± 64.5 ms after AP disturbance ($n = 7$, $p < 0.0001$). With the APqrPID intervention, the average APD_{90} was 338.3 ± 68 ms in the presence of the disturbance, differing only by 4.6 ms on average compared to CTL (Figure 4B). Without correction by APqrPID, V_m error (i.e., difference between measured and setpoint V_m) started to increase at the onset of the perturbing light pulse and reached a maximum of 43.3 mV on average (Figures 4C and S2C), distributing around zero (0 ± 2.5) mV in only 37.6% of the time with a median of 10 mV (Figure 4D). With APqrPID correction applied, V_m error fell in the range 0 ± 2.5 mV 96.7% of the time with a median of 0 mV.

AP restoration in the presence of a drug-induced electrical disturbance in multicellular preparations

Next, the efficacy of AP restoration by APqrPID was tested in the setting of drug-induced APD prolongation. To this end, 4AP was applied to hiAM monolayers expressing CheRiff and Jaws. Similarly to single hiAMs, 4AP induced marked APD prolongation, while the dual-actuator APqrPID intervention restored AP shapes to normal (Figure 5A, also shown on separate axes in Figure S3B). Average APD was increased by 91.2 ± 19, 134.6 ± 19.4, and 82.4 ± 27.8 ms with 4AP compared to CTL at 20%, 50%, and 90% repolarization levels, respectively (Figure 5B). APqrPID reduced APD difference significantly to 22.4 ± 11.8 ms (APD_{20}), 9.1 ± 16.8 ms (APD_{50}), and to as low as 1.9 ± 3.1 ms (APD_{90}) on average. As the representative recordings in Figure 5C illustrate, the APD prolongation caused by 4AP administration resulted in a positive V_m error up to 50 mV. This error instructed APqrPID to generate predominantly red light to activate Jaws, which in turn generated the outward current favoring repolarization, counteracting the effects of 4AP. Interestingly, while the maximal V_m error was reduced to 30.4 mV by APqrPID, V_m was not reversed to normal during the early repolarization phase. During this time, red light output was maximal, indicating that total Jaws conductance was insufficient. As soon as less than maximal red light output was necessary (from 78 ms onward, Figures 5C and S2D), a better efficacy of V_m correction could be achieved. V_m error indicated a V_m deviation larger than 2.5 mV 98.5% of the time with a median at 24.2 mV with 4AP compared to CTL (Figure 5D). With the application of APqrPID, V_m deviation was larger than 2.5 mV in only 21.9% of the cases and fell within −2.5 to 2.5 mV 74.7% of the time. The median value of residual V_m error during APqrPID intervention was 0.23 mV. Taken together, these results provide experimental evidence for the feasibility of accurate, closed-loop-controlled restoration of abnormal AP morphologies using two antagonizing optogenetic actuators in a multicellular preparation.

Light-driven enforcement of arbitrary AP morphologies

A method for the control of AP waveforms as presented in this study not only allows normalization of disturbed AP shapes, but it may also provide means to enforce any desired AP morphology onto cardiomyocytes in an autonomous, self-regulatory manner. To investigate this possibility, the APqrPID closed-loop control algorithm was modified in two aspects. Firstly, instead of recording reference APs under control conditions, the new controller dubbed APqrLE loads data defining reference APs (also referred to as model APs) from a file. Secondly, APqrLE produces intermittent light output (470 nm, 10 ms at 1 Hz in our experiments) in order to activate CheRiff thereby triggering APs.

Imposing models of drug-induced AP perturbations

Prior to the experiments with APqrLE, APs were recorded in hiAM monolayers exposed to carbachol, an acetylcholine receptor agonist (i.e., a drug shortening APD) or to 4AP and were used as model APs during AP waveform enforcement in n = 5 monolayers of hiAMs expressing CheRiff and Jaws (Figure 6). The model APs were enforced in the carbachol-carbachol-4AP order.

As the representative recordings in Figure 6A illustrate, hiAM monolayers were first paced electrically with APqrLE switched off to register endogenous APs. Then the electrical stimulation was terminated, and APqrLE was activated. With APqrLE

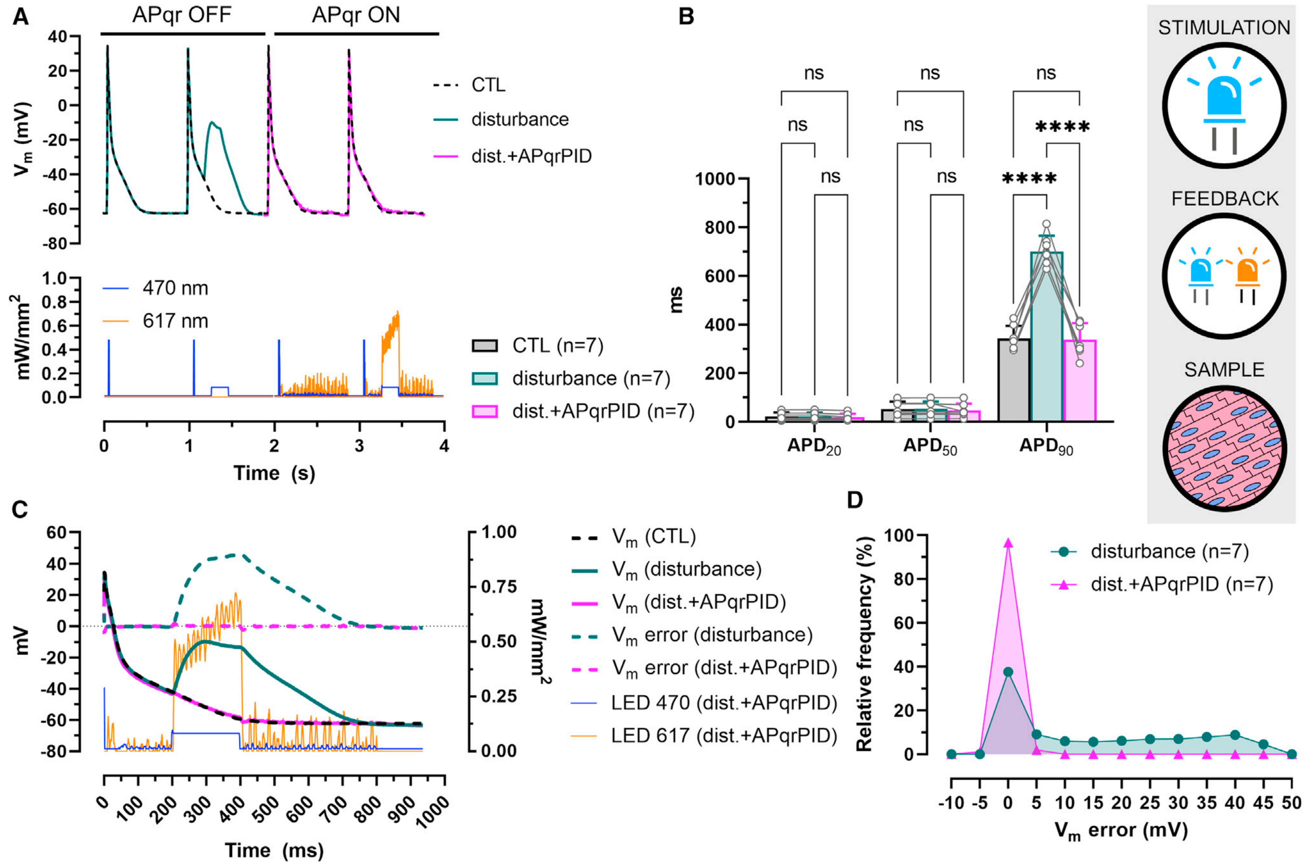

Figure 4. AP restoration in the presence of a light-induced electrical disturbance by dual-actuator optogenetics

(A) Representative recordings of optogenetically triggered APs (upper panel) in a hiAM monolayer, under normal conditions (CTL) and in the presence of a light-induced disturbance without (disturbance) and with restoration (dist. + APqrPID). Also shown is the light output of the 470- and 617-nm LEDs during AP shape restoration (lower panel).

(B) Mean APD values ±SD measured at 20%, 50%, and 90% repolarization in n = 7 hiAM monolayers. ****$p < 0.0001$, ns: not significant.

(C) Representative recordings of APs between the moment of dV/dt_{max} and the moment of APD_{90}, overlaid with V_m error calculated for the disturbed (disturbance) and restored (dist. + APqrPID) APs compared to CTL. Also shown are the 470- and 617-nm light outputs during AP shape restoration.

(D) Frequency distribution of V_m error in n = 7 monolayers before (disturbance) and during AP shape restoration (dist. + APqrPID) with a 5-mV bin width See also Figures S2C, S2H and S3A.

running, APs were triggered by blue light output every second. Following the pacing stimuli, a varying mixture of blue and red light output was generated, and coinciding with this, the real-time measured V_m was following the shape of the model APs (Figures S3C and S3D). It is important to note that while the inward chloride pump Jaws hyperpolarizes V_m in an essentially V_m-independent manner, a depolarizing effect from the light-gated ion channel CheRiff can only be expected when V_m is more negative than the reversal potential of the CheRiff current. This apparent reversal potential for CheRiff was characterized as $V_{plateau}$ for each monolayer (Figure S2H) and was set as a parameter for APqrLE in each experiment. Blue light output was only permitted when V_m was more negative than CheRiff's $V_{plateau}$ (Figure 6A).

While average APD measured at 30% and 50% repolarization levels were statistically not different between model and control APs, average APD_{90} was significantly shorter in the carbachol model (95.2 ± 0.4 ms) and prolonged in the 4AP model (422.2 ± 0.4 ms) compared to CTL (314.6 ± 53.1 ms, $p < 0.05$, Figures S4A and S4B). With APqrLE on, average, APD_{90} was shortened to 89.6 ± 3.9 ms and prolonged to 418.4 ± 3.6 ms during the enforcement of the carbachol and the 4AP model, respectively (Figures S4A and S4B). Hence, APqrLE reduced the APD_{90} differences between control and model APs from 219.4 to 107.6 ms to 5.6 and 3.8 ms for the carbachol and 4AP model, respectively. Analysis of maximal V_m (V_{peak}) and dV/dt_{max} showed that the enforcement of model APs was the least effective during the AP upstroke (Figures S4C and S4D). Since the average time to reach dV/dt_{max} was ~5 ms longer for forced compared to model APs (Figure S4E) and because this time point is used to calculate APD values, it may explain the slight APD_{90} differences that remain observable during AP enforcement. To account for this, the times at which the various repolarization levels occurred (i.e., the times at which V_m took the value corresponding to 30%, 50%, or 90% repolarization) were determined and compared between reference and forced APs (Figures S4G

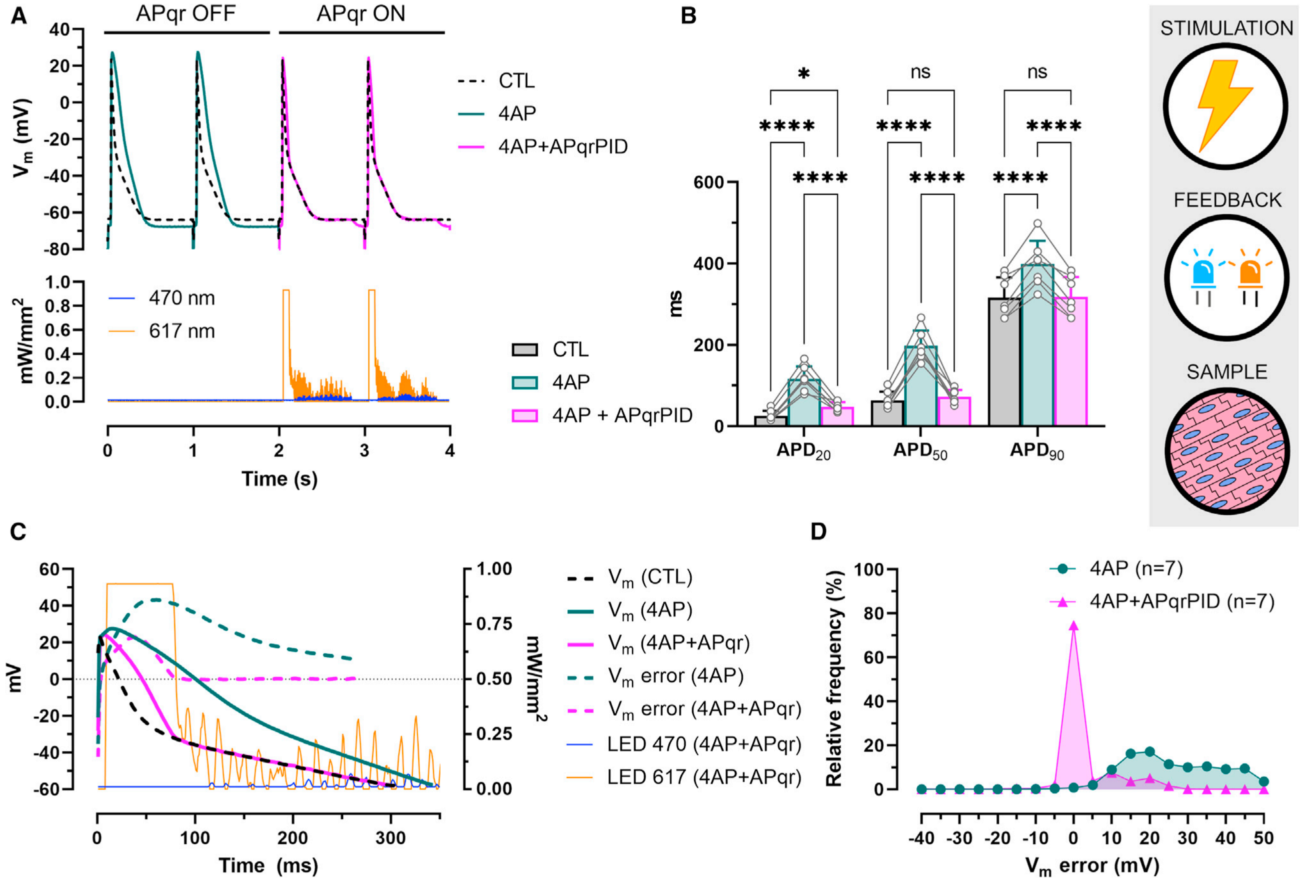

Figure 5. AP restoration in the presence of a drug-induced electrical disturbance by dual-actuator optogenetics

(A) Representative recordings of electrically triggered APs (upper panel) in a hiAM monolayer, under normal conditions (CTL), following 4AP administration (4AP), and during AP shape restoration in the presence of 4AP (4AP + APqrPID). Also shown is the light output of the 470- and 617-nm LEDs during AP shape restoration (lower panel).

(B) Mean APD values ±SD measured at 20%, 50%, and 90% repolarization in n = 7 hiAM monolayers. $^{*}p < 0.05$, $^{****}p < 0.0001$, ns: not significant.

(C) Representative recordings of APs between the moment of dV/dt_{max} and the moment of APD_{90}, overlaid with V_m error calculated for the 4AP-exposed (4AP) and vs. CTL (4AP) and 4AP vs. CTL (4AP + APqrPID) APs. Also shown are the 470- and 617-nm light outputs during AP shape restoration.

(D) Frequency distribution of V_m error in n = 7 monolayers before (4AP) and during AP shape restoration (4AP + APqrPID) with a 5-mV bin width. See also Figures S2D, S2H and S3B.

and S4H). This comparison showed the absence of statistical differences in time of APD_{30}, APD_{50}, and APD_{90} between the model APs and their enforced counterparts, with time of 90% repolarization differing as little as 1.6 and 0.4 ms for the carbachol and 4AP models, respectively (Figures S4G and S4H). Average V_{rest} values were significantly more positive in CTL compared to the carbachol (5 mV difference) and 4AP (8.7 mV difference) models. During enforcement, V_{rest} values of the corresponding models were imposed with as little as 0.005 and 0.01 mV difference on average (Figure S4F).

As the representative recordings show, predominantly red light was necessary to reproduce the carbachol model and blue light for the 4AP model (Figures 6B and 6C, also shown on separate axes in Figures S3C and S3D). In both cases, the inescapable binding of the blue light output to the V_m range negative of CheRiff's $V_{plateau}$ limited the efficacy of V_m control during the early phase of the AP. In the carbachol model, enforcement reduced the average V_m error to less than 5.5 and 2.5 mV at 16 and 22 ms after reaching dV/dt_{max}, respectively (Figure 6D). For the 4AP model, average V_m error levels below 5.5 and 2.5 mV were reached within 5 and 62 ms, respectively (Figure 6E). V_m error calculated for control APs compared to the carbachol and 4AP reference APs had median values of 7.8 and 2.7 mV, distributing within the range of −2.5 to 2.5 mV in 11% and 7.5% of the time, respectively. During the enforcement of the carbachol and 4AP models, V_m error fell in the range of 0 ± 2.5 mV in 95.8% and 94.4% of the time, and the median V_m error was reduced to −0.02 and 0.04 mV, respectively (Figures 6F and 6G).

Imposing simplified AP models

Next, the operational range of AP morphology control as carried out in our study was assessed by enforcing simplified AP waveforms by APqrLE in hiAM monolayers expressing CheRiff and Jaws (n = 5). Each of the AP models consisted of two linear phases, an upstroke and a repolarization phase. The upstroke of each model arose from −68 mV at a rate of 20 mV/ms to the

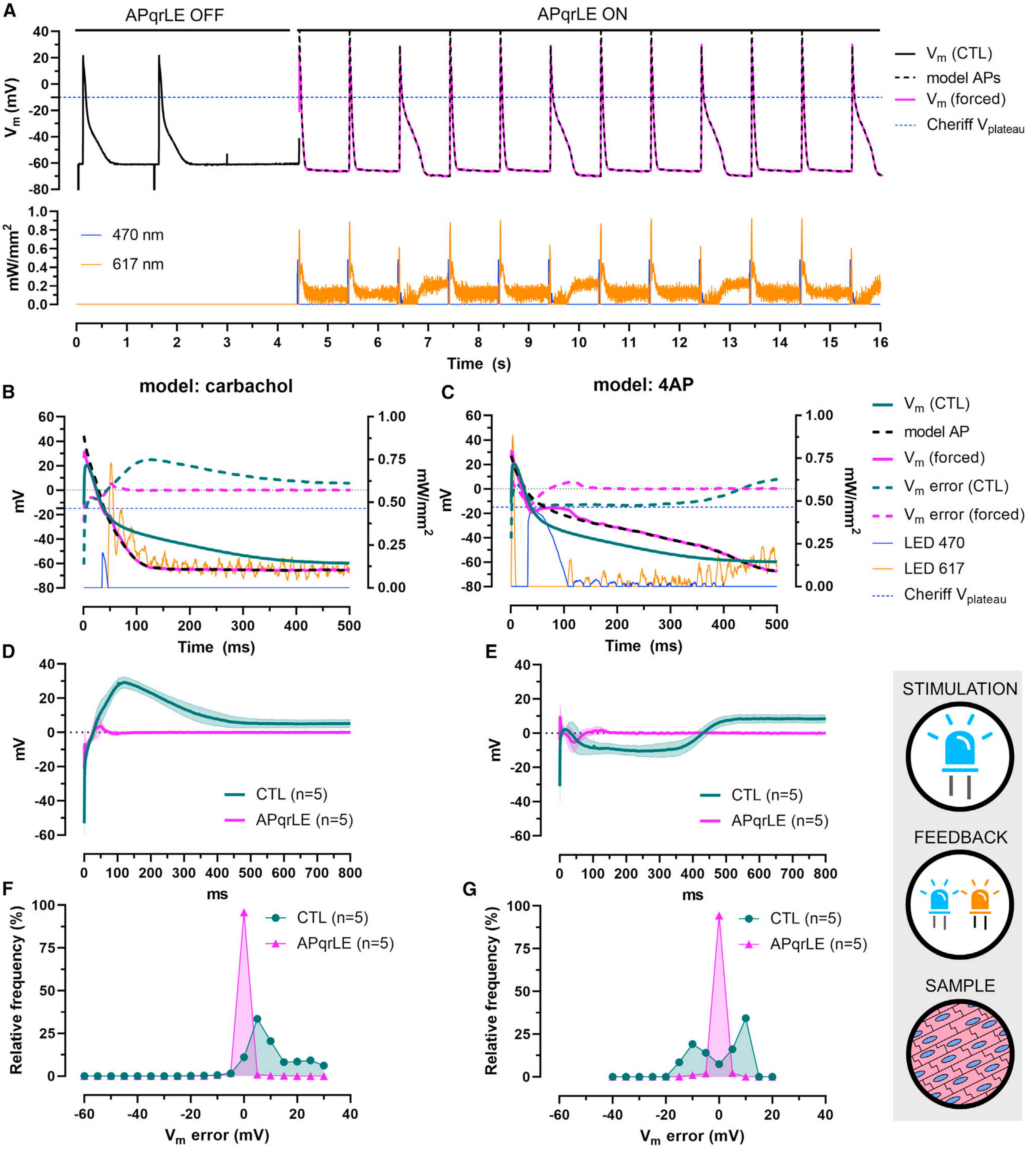

Figure 6. Imposing models of drug-induced AP perturbations in hiAM monolayers

(A) Representative recordings of electrically triggered endogenous APs (CTL) and V_m during enforcement of models of carbachol- and 4AP-induced AP morphologies in a hiAM monolayer expressing CheRiff and Jaws (upper panel) and display of the 470- and 617-nm light output (lower panel).

(B and C) Representative recordings of APs between the moment of dV/dt_{max} and the moment of APD_{90}, overlaid with the corresponding AP model and V_m error calculated for the CTL (CTL) and enforced (APqrLE) APs compared to the corresponding model APs, for the carbachols and 4AP models, respectively. Also shown are the 470- and 617-nm light outputs during AP shape enforcement. Labels shown in (C) apply to (B) as well.

(legend continued on next page)

V_{peak} at 12 mV, after which the model V_m started to decrease at constant rates of 1.3, 0.9, 0.5, or 0.1 mV/ms. The resulting triangular AP models were named as t13, t09, t05, and t01, respectively, and they were enforced in a consecutive manner following each other in the t13, t09, t05, t01, t05, t09, and t13 order.

As before, hiAM monolayers were first paced electrically to register endogenous (control) APs, after which APqrLE was activated, and the light-driven model AP enforcement started (Figures 7A and S5). Due to different repolarization rates, average APD_{90} values of model APs differed substantially from that of the endogenous APs (Figures 7B and S6A–S6D). The difference between APD_{90} of the model APs versus CTL was 245.8, 221.8, 158, and 418.4 ms on average, which was reduced during V_m enforcement by APqrLE to 1.8, 8.8, 10.2, and 11 ms for models t13, t09, t05, and t01, respectively (Figure 7B). Notably, such outcome necessitated forced APD_{90} shortening for models t13, t09, and t05 and prolongation for model t01. The residual APD_{90} difference could be attributed to the fact that the upstroke phase of forced APs followed the model APs the least, as illustrated by V_{peak}, dV/dt_{max}, and time to reach dV/dt_{max} (Figures S6E–S6G). Indeed, the time of 90% repolarization deviated by less than 1 ms between model and forced APs for model t01 (0.6 ms) and t05 (−0.2 ms), and the difference was only 1.6 ms for model t09 on average, while it was enforced the least accurately in the case of model t13 (−13.4 ms difference, Figure S6H). Apparently, the maximal ion flux produced by Jaws did not always suffice to fully impose model t13 as well as the early phase of model t09, as indicated by the red light output reaching its maximum during the enforcement of these AP models (Figures 7D and 7E, also shown in Figure S5 on separate axes). Resting potentials (V_{rest}) were more positive in control APs compared to model APs (6.9 mV difference, n = 5, $p < 0.05$), while V_{rest} during enforcement differed by less than 0.7 mV on average (Figure S6I).

V_m error was reduced to the range of −2.5 to 2.5 mV within the first 50 ms for models t09 and t05 (Figures 7E, 7F, S7B, and S7C) and within the first 80 ms for model t13 (Figures 7D and S7A) during the generation of forced APs. V_m error was reduced to fall within this range during the enforcement of model AP t01 as well, albeit only after 280 ms on average (Figures 7G and S7D). This was due to the fact that CheRiff could only produce a depolarizing current below its $V_{plateau}$. V_m error calculated for control versus model APs of all four models combined had a median value of 7.5 mV with 28% of data points distributing around zero (0 ± 2.5) mV (Figure 7C). Despite the aforementioned limitations, the APqrLE intervention reduced the median V_m error to −0.005 mV, with 87.6% of data points falling within the range of 0 ± 2.5 mV (Figure 7C). The relatively high occurrence of residual V_m error is due to the ion channel nature of CheRiff, which affected the enforcement of the t01 model the most (Figure S7H). For all other models, the frequency of V_m error between −2.5 and 2.5 mV was higher than 92% (Figures S7E–S7G).

Taken together, these data illustrate the feasibility of dynamic enforcement of arbitrary AP waveforms in multicellular cardiomyocyte preparations by a closed-loop-driven dual-actuator optogenetic approach.

DISCUSSION

In this study, we realized accurate restoration and preservation of cardiac AP waveforms in the presence of electrical perturbations of different origin in an unsupervised, self-regulatory manner, without any prior knowledge of the disturbance. We achieved this by the design and realization of an experimental system consisting of elements from patch-clamp electrophysiology, hard real-time computing, and optogenetics integrated in a closed-loop arrangement. Feedback loop control was accomplished by three different custom algorithms, APqr, APqrPID, and APqrLE. APqr was designed to target V_m by means of direct current injection or activation of a single optogenetic actuator by light. The PID closed-loop control principle was implemented in APqrPID designed to operate on two optogenetic actuators with antagonizing effects. APqrLE was based on PID control principles as well and designed to enforce arbitrary AP waveforms. We showed effective correction of AP morphology changes, particularly correction of APD prolongation both by current injection and illumination in single cardiomyocytes using APqr and in monolayers of cardiomyocytes using dual-actuator optogenetics by APqrPID. Effective V_m control in cardiomyocyte monolayers was further evidenced by the enforcement of arbitrary AP shapes, including AP morphologies with drug-induced abnormalities and simplified, triangle-like AP models by the application of APqrLE.

Feedback loop-driven system control has several beneficial features. Most importantly, it enables delivery of interventions on demand (i.e., only when needed), which can be especially useful in the management of conditions that manifest in an episodic manner, such as vasovagal syncope, intractable neuropathic pain, or epilepsy.[34,35] On the other hand, the inherent self-regulatory nature of closed-loop systems' control eliminates the need of extensive prior calibration and allows the use of actuators with non-linear input-output relationships, such as ChRs.[11] In the experimental setting, feedback loops implemented in "closed-loop optogenetics" have opened new avenues for the interrogation of neuronal activity in behaving animals and have already seen several applications.[36,37] In general, such approaches aim to modulate the activity of single neurons or neuronal circuits (in essence their firing rate) by means of optogenetic stimulation in response to physiological, electrical, or optical readouts. Recent developments in the field have been focusing on the realization of all-optical closed-loop platforms, which combine optical readout and photostimulation.[24,38] These systems have the unique capacity to interrogate neural circuits in living animals in a fully non-invasive way. However, the low signal-to-noise ratio of the optical V_m readout only allows relatively low closed-loop update rates (i.e., the rate at which V_m input is sampled and light output is generated), preventing the

(D and E) V_m error representing the difference between model vs. endogenous APs (CTL) and model vs. enforced APs (APqrLE) over time for the carbachol and 4AP models, respectively, in n = 5 hiAM monolayers. Data are shown as mean (continuous line) ±SD (shaded area).
(F and G) Frequency distribution of V_m error in n = 5 monolayers before (CTL) and during AP shape enforcement (APqrLE) with a 5-mV bin width for the carbachol and 4AP models, respectively. See also Figures S3C, S3D, and S4.

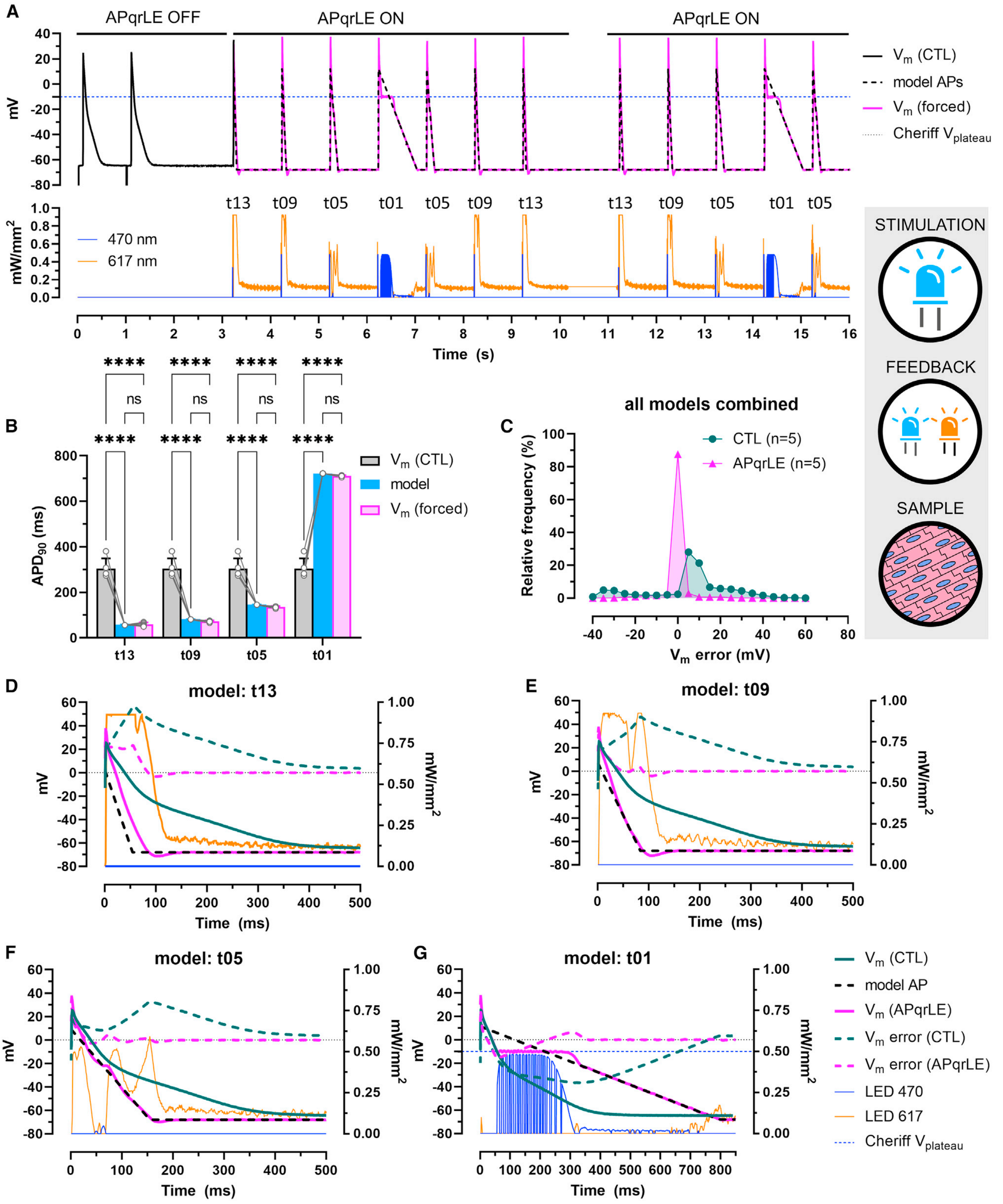

Figure 7. Imposing simplified AP models in hiAM monolayers

(A) Representative recordings of electrically triggered endogenous APs (CTL) and V_m during enforcement of AP models t13, t09, t05, and t01 in a hiAM monolayer expressing CheRiff and Jaws (upper panel) and display of the 470- and 617-nm light output (lower panel).

(legend continued on next page)

control of fast processes with physiologically relevant accuracy. Here, we reasoned that by improving the temporal resolution of closed-loop optogenetics, it would be possible to fully control excitability, including not only the frequency of activated states (i.e., the firing rate of neurons or the heart rate) but also the shape of individual cardiac APs. We sought to achieve this by ensuring sufficiently fast feedback loop update rates. For this reason, we opted for patch-clamp electrophysiology, capable of acquiring V_m readout at high sampling rates. This strategy made an update rate of 1 kHz possible, which was key to achieve millisecond and millivolt accuracy V_m control.

Limitations of the study

While demonstrating the ability to control V_m in cardiomyocytes with physiologically relevant accuracy and precision by dual-channel closed-loop optogenetics in the multicellular setting, we also identified key areas where further development is needed in order to exploit the beneficial features of the technology presented here in full.

Due to the electrical coupling between cardiomyocytes, an AP recorded by the patch-clamp technique from a specific location within a hiAM monolayer represents a so-called compound AP, the summation of APs generated by cells in and surrounding that location, which is expected to be uniform in all areas of the monolayer. However, local electrical or optogenetic pacing results in activation waves spreading from the site of pacing through the monolayer. Consequently, V_m control by APqr, as carried out in this study, was optimal at the site of recording but not in the whole monolayer. This limitation could be overcome, on the short term, by using homogeneous light sources delivering the same light intensity throughout the entire monolayer. Optogenetic pacing by a homogeneous light source would eliminate differences in local activation times. Because of the synchronous activation, the homogeneously applied corrective light output from APqr would result in optimal V_m control for the entire monolayer, despite the dependence on local V_m readout. Alternatively, recent developments in nanotechnology, such as 3D nanoelectrode arrays, could be exploited in order to obtain local, intracellular V_m recordings.[39] In combination with patterned photostimulation[40] and the APqr technology, this would allow high-resolution V_m control in both space and time.

The performance of PID controllers is largely determined by three mutually dependent gain parameters K_P, K_D, and K_I (Equation 10). Setting optimal values for the gain parameters (i.e., controller tuning) is typically carried out by established procedures, such as the so-called Ziegler-Nichols method. We found that using gain parameters determined by this method produced suboptimal outcomes, such as ineffective V_m control or oscillations, likely due to the non-linear dependence of V_m on effector output and the fast kinetics of V_m changes during the AP. Therefore, in our experiments, controller tuning was finalized by a mainly heuristic approach, which typically resulted in different gain parameters in each experiment (K_P: 40–120, K_I: 1–5, K_D: 5–200). The optimal values of gain parameters and their dependence on experimental conditions could be determined in dedicated studies, which would potentially result in further improvements in controller performance. Alternatively, auto-tuning principles could be implemented in the closed-loop controller, which would allow controller tuning in an unsupervised way, further strengthening the applicability of our approach.[33]

In addition, the selection of optogenetic actuators could be tailored to each specific application. First, the permeability of the opsin of choice to different ionic species should be considered with respect to the potential effects of changes to the ionic milieu occurring in response to opsin activation. For example, the inward chloride pump Jaws could be replaced by outward proton pumps in cases where cytosolic chloride concentrations are critical.[21] Also, the relative cation conductance of ChR variants can be considered.[41] Furthermore, the operational range of the experimental system presented here could be extended, primarily by the use of optogenetic actuators with functional properties matching a particular application. For example, the V_m range in which depolarizing effects are available could be broadened by replacing CheRiff by an optogenetic actuator that facilitates active inward cation or outward anion transport in a V_m-independent fashion (i.e., an ion pump). Promising alternatives are the light-activatable inward proton pumps belonging to the families of xenorhodopsins and schizorhodopsins.[42,43] However, the action spectrum of SzR3 centered at 525 nm overlaps with known optogenetic actuators having hyperpolarizing effects, which prevents application of SzR3 in the dual-actuator arrangement. The recent rapid development of microbial rhodopsin research, including both the discovery of natural variants and deeper understanding of structure-function relationships for rational color tuning, holds the promise of overcoming this limitation in the near future.[44] Furthermore, Jaws could be directly replaced by the most recently described potassium ChR KCR1.[45] KCR1 can be activated by red light (action spectrum peak: 540 nm), allowing application in combination with blue light-activatable actuators. Importantly, displaying a reversal potential at −85 mV, KCR1 is expected to generate large outward current amplitudes driven by the great driving force for potassium in the positive V_m range. These characteristics hint that sufficient hyperpolarizing effect could be achieved by using KCR1 during the early phase of repolarization, when Jaws function appears to be the least optimal.

Once these limitations have been dealt with effectively, we foresee imminent applications of opto-electronic closed-loop-controlled systems for V_m control in various research areas, These could range, for example, from the regulatory roles of V_m in cellular differentiation and homeostasis to its impact on

(B) Mean APD values ±SD measured at 20%, 50%, and 90% repolarization in n = 5 hiAM monolayers. ****$p < 0.0001$, ns: not significant.

(C) Frequency distribution of V_m error for all AP models combined in n = 5 monolayers before (CTL) and during AP shape enforcement (APqrLE) with 5-mV bin width.

(D–G) Representative recordings of APs between the moment of dV/dt_{max} and the moment of APD_{90}, overlaid with the corresponding AP model and V_m error calculated as the difference between CTL and model APs (CTL) and enforced and model APs (APqrLE) for the t13, t09, t05, and t01 models, respectively. Also shown are the 470- and 617-nm light outputs during AP shape enforcement. Labels shown in (G) apply to (D)–(F) as well. See also Figures S5–S7.

disease development and progression. As to the latter subject, a hallmark of many heart diseases including atrial fibrillation and heart failure is electrical remodeling.[46,47] The resulting changes in the electrophysiological properties of the heart are often associated with deleterious consequences. In atrial fibrillation, for example, rapid electrical activation increases Ca^{2+} exposure of atrial myocytes, which triggers compensatory mechanisms leading to refractory period shortening as a manifestation of electrical remodeling, which in turn increases atrial fibrillation propensity.[48] In heart failure, electrical remodeling includes the loss of the "notch" in the "spike-notch-dome"-shaped APs typically displayed by healthy ventricular myocytes. Interestingly, clamping V_m of healthy cells to a failing AP shape has robust influence on Ca^{2+} handling in isolated single cells in the acute setting.[49] While such association of electrical remodeling to disease conditions is well documented in many cases, the cause and effect relationships and the detailed molecular mechanisms remain elusive. Using APqr, cardiomyocytes cultured in multicellular format could, for example, be exposed to AP morphologies characteristic of pathological conditions, which would allow dissection of cause and effect, while creating possibilities to study the underlying mechanisms in a controlled and detailed manner.

Collectively, this study sets the stage for the refinement and application of opto-electronic control systems to enable in-depth investigation into the regulatory roles of V_m in health and disease.

STAR★METHODS

Detailed methods are provided in the online version of this paper and include the following:

- KEY RESOURCES TABLE
- RESOURCE AVAILABILITY
  - Lead contact
  - Materials availability
  - Data and code availability
- EXPERIMENTAL MODEL AND STUDY PARTICIPANT DETAILS
  - hiAM cell culture
- METHOD DETAILS
  - Generation and production of lentiviral vectors
  - (Opto)genetic modification of hiAMs
  - Patch clamp electrophysiology
  - Instrumentation
  - Closed-loop control
- QUANTIFICATION AND STATISTICAL ANALYSIS

SUPPLEMENTAL INFORMATION

Supplemental information can be found online at https://doi.org/10.1016/j.crmeth.2023.100671.

ACKNOWLEDGMENTS

We thank Teun P. de Boer, UMC Utrecht, for his helpful advice on real-time data acquisition and processing. This work was supported by the European Research Council (starting grant 716509 and consolidator grant 101044831 to D.A.P.).

AUTHOR CONTRIBUTIONS

Conceptualization, B.Ö., T.D.C., A.A.F.d.V., and D.A.P.; methodology, B.Ö., T.D.C., B.L.d.O., S.D., and A.A.F.d.V.; software, T.D.C. and B.Ö.; investigation, B.Ö., S.O.D., C.I.B., J.Z., and W.H.B.; visualization, B.Ö. and T.D.C.; writing – original draft, B.Ö., T.D.C., and D.A.P.; writing – review and editing, B.Ö., T.D.C., A.A.F.d.V., D.A.P., and G.J.J.B.

DECLARATION OF INTERESTS

The authors declare no competing interests.

Received: May 12, 2023
Revised: October 9, 2023
Accepted: November 19, 2023
Published: December 11, 2023

REFERENCES

1. Schofield, Z., Meloni, G.N., Tran, P., Zerfass, C., Sena, G., Hayashi, Y., Grant, M., Contera, S.A., Minteer, S.D., Kim, M., et al. (2020). Bioelectrical understanding and engineering of cell biology. J. R. Soc. Interface *17*, 20200013.
2. Bean, B.P. (2007). The action potential in mammalian central neurons. Nat. Rev. Neurosci. *8*, 451–465.
3. Nerbonne, J.M., and Kass, R.S. (2005). Molecular physiology of cardiac repolarization. Physiol. Rev. *85*, 1205–1253.
4. Krijger Juárez, C., Amin, A.S., Offerhaus, J.A., Bezzina, C.R., and Boukens, B.J. (2022). Cardiac Repolarization in Health and Disease. JACC: Clinical Electrophysiology *9*, 124–138.
5. Horváth, B., Szentandrássy, N., Dienes, C., Kovács, Z.M., Nánási, P.P., Chen-Izu, Y., Izu, L.T., and Banyasz, T. (2022). Exploring the Coordination of Cardiac Ion Channels With Action Potential Clamp Technique. Front. Physiol. *13*, 864002.
6. Wilders, R. (2006). Dynamic clamp: a powerful tool in cardiac electrophysiology. J. Physiol. *576*, 349–359.
7. Mitterdorfer, J., and Bean, B.P. (2002). Potassium currents during the action potential of hippocampal CA3 neurons. J. Neurosci. *22*, 10106–10115.
8. Berecki, G., Howell, K.B., Heighway, J., Olivier, N., Rodda, J., Overmars, I., Vlaskamp, D.R.M., Ware, T.L., Ardern-Holmes, S., Lesca, G., et al. (2022). Functional correlates of clinical phenotype and severity in recurrent SCN2A variants. Commun. Biol. *5*, 515.
9. Harris, D.M., Mills, G.D., Chen, X., Kubo, H., Berretta, R.M., Votaw, V.S., Santana, L.F., and Houser, S.R. (2005). Alterations in early action potential repolarization causes localized failure of sarcoplasmic reticulum Ca2+ release. Circ. Res. *96*, 543–550.
10. Schneider, F., Grimm, C., and Hegemann, P. (2015). Biophysics of Channelrhodopsin. Annu. Rev. Biophys. *44*, 167–186.
11. Ördög, B., Teplenin, A., De Coster, T., Bart, C.I., Dekker, S.O., Zhang, J., Ypey, D.L., de Vries, A.A.F., and Pijnappels, D.A. (2021). The Effects of Repetitive Use and Pathological Remodeling on Channelrhodopsin Function in Cardiomyocytes. Front. Physiol. *12*, 710020.
12. Arrenberg, A.B., Stainier, D.Y.R., Baier, H., and Huisken, J. (2010). Optogenetic control of cardiac function. Science *330*, 971–974.
13. Bruegmann, T., Malan, D., Hesse, M., Beiert, T., Fuegemann, C.J., Fleischmann, B.K., and Sasse, P. (2010). Optogenetic control of heart muscle in vitro and in vivo. Nat. Methods *7*, 897–900.
14. Nussinovitch, U., and Gepstein, L. (2015). Optogenetics for in vivo cardiac pacing and resynchronization therapies. Nat. Biotechnol. *33*, 750–754.
15. Nyns, E.C.A., Jin, T., Fontes, M.S., van den Heuvel, T., Portero, V., Ramsey, C., Bart, C.I., Zeppenfeld, K., Schalij, M.J., van Brakel, T.J., et al. (2022). Optical ventricular cardioversion by local optogenetic targeting and LED implantation in a cardiomyopathic rat model. Cardiovasc. Res. *118*, 2293–2303.

16. Nyns, E.C.A., Poelma, R.H., Volkers, L., Plomp, J.J., Bart, C.I., Kip, A.M., van Brakel, T.J., Zeppenfeld, K., Schalij, M.J., Zhang, G.Q., et al. (2019). An automated hybrid bioelectronic system for autogenous restoration of sinus rhythm in atrial fibrillation. Sci. Transl. Med. *11*, eaau6447.

17. Bingen, B.O., Engels, M.C., Schalij, M.J., Jangsangthong, W., Neshati, Z., Feola, I., Ypey, D.L., Askar, S.F.A., Panfilov, A.V., Pijnappels, D.A., and de Vries, A.A.F. (2014). Light-induced termination of spiral wave arrhythmias by optogenetic engineering of atrial cardiomyocytes. Cardiovasc. Res. *104*, 194–205.

18. Burton, R.A.B., Klimas, A., Ambrosi, C.M., Tomek, J., Corbett, A., Entcheva, E., and Bub, G. (2015). Optical control of excitation waves in cardiac tissue. Nat. Photonics *9*, 813–816.

19. Majumder, R., Feola, I., Teplenin, A.S., de Vries, A.A., Panfilov, A.V., and Pijnappels, D.A. (2018). Optogenetics enables real-time spatiotemporal control over spiral wave dynamics in an excitable cardiac system. Elife *7*, e41076.

20. Scardigli, M., Müllenbroich, C., Margoni, E., Cannazzaro, S., Crocini, C., Ferrantini, C., Coppini, R., Yan, P., Loew, L.M., Campione, M., et al. (2018). Real-time optical manipulation of cardiac conduction in intact hearts. J. Physiol. *596*, 3841–3858.

21. Newman, J.P., Fong, M.F., Millard, D.C., Whitmire, C.J., Stanley, G.B., and Potter, S.M. (2015). Optogenetic feedback control of neural activity. Elife *4*, e07192.

22. Streit, J., and Kleinlogel, S. (2018). Dynamic all-optical drug screening on cardiac voltage-gated ion channels. Sci. Rep. *8*, 1153.

23. Quach, B., Krogh-Madsen, T., Entcheva, E., and Christini, D.J. (2018). Light-Activated Dynamic Clamp Using iPSC-Derived Cardiomyocytes. Biophys. J. *115*, 2206–2217.

24. Bergs, A.C.F., Liewald, J.F., Rodriguez-Rozada, S., Liu, Q., Wirt, C., Bessel, A., Zeitzschel, N., Durmaz, H., Nozownik, A., Dill, H., et al. (2023). All-optical closed-loop voltage clamp for precise control of muscles and neurons in live animals. Nat. Commun. *14*, 1939.

25. Karathanos, T.V., Boyle, P.M., and Trayanova, N.A. (2014). Optogenetics-enabled dynamic modulation of action potential duration in atrial tissue: feasibility of a novel therapeutic approach. Europace *16* (*Suppl 4*), iv69–iv76.

26. Park, S.A., Lee, S.R., Tung, L., and Yue, D.T. (2014). Optical mapping of optogenetically shaped cardiac action potentials. Sci. Rep. *4*, 6125.

27. Govorunova, E.G., Cunha, S.R., Sineshchekov, O.A., and Spudich, J.L. (2016). Anion channelrhodopsins for inhibitory cardiac optogenetics. Sci. Rep. *6*, 33530.

28. Gruber, A., Edri, O., Huber, I., Arbel, G., Gepstein, A., Shiti, A., Shaheen, N., Chorna, S., Landesberg, M., and Gepstein, L. (2021). Optogenetic modulation of cardiac action potential properties may prevent arrhythmogenesis in short and long QT syndromes. JCI Insight *6*, e147470.

29. Billman, G.E. (2020). Homeostasis: The Underappreciated and Far Too Often Ignored Central Organizing Principle of Physiology. Front. Physiol. *11*, 200.

30. Hochbaum, D.R., Zhao, Y., Farhi, S.L., Klapoetke, N., Werley, C.A., Kapoor, V., Zou, P., Kralj, J.M., Maclaurin, D., Smedemark-Margulies, N., et al. (2014). All-optical electrophysiology in mammalian neurons using engineered microbial rhodopsins. Nat. Methods *11*, 825–833.

31. Chuong, A.S., Miri, M.L., Busskamp, V., Matthews, G.A.C., Acker, L.C., Sørensen, A.T., Young, A., Klapoetke, N.C., Henninger, M.A., Kodandaramaiah, S.B., et al. (2014). Noninvasive optical inhibition with a red-shifted microbial rhodopsin. Nat. Neurosci. *17*, 1123–1129.

32. Harlaar, N., Dekker, S.O., Zhang, J., Snabel, R.R., Veldkamp, M.W., Verkerk, A.O., Fabres, C.C., Schwach, V., Lerink, L.J.S., Rivaud, M.R., et al. (2022). Conditional immortalization of human atrial myocytes for the generation of in vitro models of atrial fibrillation. Nat. Biomed. Eng. *6*, 389–402.

33. Kiam Heong, A., Chong, G., and Yun, L. (2005). PID control system analysis, design, and technology. IEEE Trans. Control Syst. Technol. *13*, 559–576.

34. Krook-Magnuson, E., Gelinas, J.N., Soltesz, I., and Buzsáki, G. (2015). Neuroelectronics and Biooptics: Closed-Loop Technologies in Neurological Disorders. JAMA Neurol. *72*, 823–829.

35. Occhetta, E., Bortnik, M., Audoglio, R., and Vassanelli, C.; INVASY Study Investigators (2004). Closed loop stimulation in prevention of vasovagal syncope. Inotropy Controlled Pacing in Vasovagal Syncope (INVASY): a multicentre randomized, single blind, controlled study. Europace *6*, 538–547.

36. Grosenick, L., Marshel, J.H., and Deisseroth, K. (2015). Closed-loop and activity-guided optogenetic control. Neuron *86*, 106–139.

37. Siegle, J.H., and Wilson, M.A. (2014). Enhancement of encoding and retrieval functions through theta phase-specific manipulation of hippocampus. Elife *3*, e03061.

38. Zhang, Z., Russell, L.E., Packer, A.M., Gauld, O.M., and Häusser, M. (2018). Closed-loop all-optical interrogation of neural circuits in vivo. Nat. Methods *15*, 1037–1040.

39. Xu, D., Mo, J., Xie, X., and Hu, N. (2021). In-Cell Nanoelectronics: Opening the Door to Intracellular Electrophysiology. Nano-Micro Lett. *13*, 127.

40. Ronzitti, E., Ventalon, C., Canepari, M., Forget, B.C., Papagiakoumou, E., and Emiliani, V. (2017). Recent advances in patterned photostimulation for optogenetics. J. Opt. *19*, 113001.

41. Kleinlogel, S., Feldbauer, K., Dempski, R.E., Fotis, H., Wood, P.G., Bamann, C., and Bamberg, E. (2011). Ultra light-sensitive and fast neuronal activation with the Ca(2)+-permeable channelrhodopsin CatCh. Nat. Neurosci. *14*, 513–518.

42. Shevchenko, V., Mager, T., Kovalev, K., Polovinkin, V., Alekseev, A., Juettner, J., Chizhov, I., Bamann, C., Vavourakis, C., Ghai, R., et al. (2017). Inward H(+) pump xenorhodopsin: Mechanism and alternative optogenetic approach. Sci. Adv. *3*, e1603187.

43. Inoue, K., Tsunoda, S.P., Singh, M., Tomida, S., Hososhima, S., Konno, M., Nakamura, R., Watanabe, H., Bulzu, P.A., Banciu, H.L., et al. (2020). Schizorhodopsins: A family of rhodopsins from Asgard archaea that function as light-driven inward H(+) pumps. Sci. Adv. *6*, eaaz2441.

44. Karasuyama, M., Inoue, K., Nakamura, R., Kandori, H., and Takeuchi, I. (2018). Understanding Colour Tuning Rules and Predicting Absorption Wavelengths of Microbial Rhodopsins by Data-Driven Machine-Learning Approach. Sci. Rep. *8*, 15580.

45. Govorunova, E.G., Gou, Y., Sineshchekov, O.A., Li, H., Lu, X., Wang, Y., Brown, L.S., St-Pierre, F., Xue, M., and Spudich, J.L. (2022). Kalium channelrhodopsins are natural light-gated potassium channels that mediate optogenetic inhibition. Nat. Neurosci. *25*, 967–974.

46. Varró, A., Tomek, J., Nagy, N., Virág, L., Passini, E., Rodriguez, B., and Baczkó, I. (2021). Cardiac transmembrane ion channels and action potentials: cellular physiology and arrhythmogenic behavior. Physiol. Rev. *101*, 1083–1176.

47. Nattel, S., Maguy, A., Le Bouter, S., and Yeh, Y.H. (2007). Arrhythmogenic ion-channel remodeling in the heart: heart failure, myocardial infarction, and atrial fibrillation. Physiol. Rev. *87*, 425–456.

48. Nattel, S., Heijman, J., Zhou, L., and Dobrev, D. (2020). Molecular Basis of Atrial Fibrillation Pathophysiology and Therapy: A Translational Perspective. Circ. Res. *127*, 51–72.

49. Cooper, P.J., Soeller, C., and Cannell, M.B. (2010). Excitation-contraction coupling in human heart failure examined by action potential clamp in rat cardiac myocytes. J. Mol. Cell. Cardiol. *49*, 911–917.

50. Patel, Y.A., George, A., Dorval, A.D., White, J.A., Christini, D.J., and Butera, R.J. (2017). Hard real-time closed-loop electrophysiology with the Real-Time eXperiment Interface (RTXI). PLoS Comput. Biol. *13*, e1005430.

STAR★METHODS

KEY RESOURCES TABLE

REAGENT or RESOURCE	SOURCE	IDENTIFIER
**Bacterial and virus strains**		
LV.HsUBC.Jaws ~ eGFP.IRES.PurR.hHBVPRE	this paper	N/A
LV.GgTnnt2.CheRiff ~ eGFP.WHVoPRE	this paper	N/A
**Chemicals, peptides, and recombinant proteins**		
NaCl	Merck	Cat# 106404
KCl	Merck	Cat# 104936
$CaCl_2$	Merck	Cat# 102382
$MgCl_2$	Merck	Cat# 105833
HEPES	VWR	Cat# 0511
NaOH	Merck	Cat# 106498
KOH	Merck	Cat# 5021
Amphotericin B	Sigma-Aldrich	Cat# A2411
Potassium D-gluconate	Merck	Cat# G4500
4-Aminopiridine	Sigma-Aldrich	Cat# 275875
Carbachol (Miostat, 0.1 mg/ml)	Alcon	N/A
**Experimental models: Cell lines**		
hiAM cell line	Harlaar et al.[32]	N/A
Jaws-hiAM cell line	this paper	N/A
**Recombinant DNA**		
Jaws-KGC-GFP-ER2	Addgene	Addgene_65012
CheRiff-eGFP	Addgene	Addgene_51693
pLV.HsUBC.Jaws ~ eGFP.IRES.PurR.hHBVPRE	this paper	N/A
pLV.GgTnnt2.CheRiff ~ eGFP.WHVoPRE	this paper	N/A
**Software and algorithms**		
RTXI	Patel et al.[50]	http://rtxi.org
APqr-software	this paper	https://doi.org/10.5281/zenodo.10116142
Python version 3.3	Python Software Foundation	https://www.python.org

RESOURCE AVAILABILITY

Lead contact

Further information and requests for resources and reagents should be directed to the lead contact, Daniël A. Pijnappels (d.a.pijnappels@lumc.nl)

Materials availability

Materials can be obtained from the lead contact after appropriate scientific review and a completed material transfer agreement.

Data and code availability

- Patch-clamp recordings reported in this paper will be shared by the lead contact upon request.
- All original code has been deposited at Zenodo: https://doi.org/10.5281/zenodo.10116142.
- Any additional information required to reanalyze the data reported in this paper is available from the lead contact upon request.

EXPERIMENTAL MODEL AND STUDY PARTICIPANT DETAILS

hiAM cell culture

Proliferation and differentiation of hiAMs were carried out as previously described.[32] Briefly, hiAMs were proliferated in the presence of doxycycline (DOX, 100 ng/mL, Sigma-Aldrich). For differentiation, hiAMs were seeded on bovine fibronectin (Sigma-Aldrich)-coated

glass coverslips in 24-well culture plates at a density 3.75×10^5 cells per well and cultured in medium without DOX. Experiments on monolayers were performed between day 12 and 14 of differentiation on coverslip-adhered hiAM monolayers transferred directly to the patch clamp bath. For single cell experiments, hiAM monolayers were dissociated on day 12 of differentiation by papain treatment. Dissociated hiAMs were re-seeded at a density of 2×10^4 cells per well of 24-well culture plates and allowed to adhere to freshly coated coverslips overnight.

The hiAM proliferation medium consisted of Advanced DMEM/F-12 (Thermo Fisher Scientific, 12634), 2 mM GlutaMAX (Thermo Fisher Scientific), 2% FBS (Biowest), 100 units/mL of penicillin and 100 μg/mL of streptomycin (Thermo Fisher Scientific) and DOX (100 ng/mL, Sigma-Aldrich). After seeding for differentiation, the hiAM differentiation medium was used consisting of Advanced DMEM/F-12 (Thermo Fisher Scientific, 12634), 2 mM GlutaMAX (Thermo Fisher Scientific) and 2% FBS (Biowest). Starting from day 4, the hiAM differentiation medium was supplemented with triiodo-L-thyronine (20 ng/mL, Sigma-Aldrich), dexamethasone (400 ng/mL, Centrafarm), LF3 (8 μM, Selleck Chemicals) and phenylephrine (10 μM, Sigma-Aldrich).

For experiments on single cells, differentiated hiAM monolayers were dissociated on day 12 of differentiation by incubation with a solution containing 5 U/mL papain (Worthington Biochemical) and 1 mM L-cysteine (Sigma-Aldrich) in phosphate-buffered saline (PBS) for 5–10 min at 37°C, after which an equal volume of stop solution was added, consisting of 1 mg/mL soybean trypsin inhibitor (Sigma-Aldrich) and 40 μg/mL DNase I (Sigma-Aldrich) in PBS. Cells were pelleted by gentle centrifugation (150×*g*, 5 min), resuspended in hiAM differentiation medium and reseeded at a density of 2×10^4 cells per well on coverslips freshly coated with bovine fibronectin (Sigma-Aldrich). The cells were allowed to adhere to the coverslips overnight and well-separated single hiAMs were subjected to patch clamp analysis the next day.

METHOD DETAILS

Generation and production of lentiviral vectors

The expression plasmids encoding enhanced green fluorescent protein (eGFP)-tagged versions of CheRiff and Jaws were obtained from Addgene (Addgene plasmid numbers 51693 and 65012, respectively). CheRiff- and Jaws-coding sequences were subcloned to yield the lentiviral shuttle constructs pLV.GgTnnt2.CheRiff ~ eGFP.WHVoPRE and pLV.HsUBC.Jaws ~ eGFP.IRES.PurR.hHBVPRE, respectively (Figure S1), using standard laboratory techniques. Lentiviral vectors were produced using the second-generation packaging plasmids psPAX2 and pLP/VSVG as described previously.[32]

(Opto)genetic modification of hiAMs

A polyclonal hiAM cell line stably expressing Jaws was created by transducing 10^6 proliferating hiAM clone 2.38 cells in one well of a 6-well culture plate with the LV.HsUBC.Jaws ~ eGFP.IRES.PurR.hHBVPRE lentiviral vector at a transduction rate of ~50% and subsequent selection by puromycin (2 μg/mL, Thermo Fisher Scienetific). Jaws-hiAMs were proliferated while maintaining selection pressure. For the simultaneous ectopic expression of CheRiff and Jaws, 3.75×10^5 proliferating Jaws-hiAMs were incubated with the LV.GgTnnt2.CheRiff ~ eGFP.WHVoPRE lentiviral vector in an *ad hoc* manner in the presence of DEAE-dextran (5 μg/mL, Carl Roth) and 100 ng/mL DOX for 24 h.

hiAMs express KCNJ5, the gene encoding an essential pore-forming subunit of I_{KACh} ion channels, at a very low level.[32] In order to establish an AP model that represents the effects of carbachol on hiAM APs (i.e., shortened APD), a polyclonal hiAM line stably expressing KCNJ5 was established by using the LV.HsPGK1.HsKCNJ5.IRES.PurR.hHBVPRE lentiviral vector as described for Jaws-hiAMs. Jaws-, CheRiff-Jaws- and KCNJ5-hiAMs were differentiated as described above.

Patch clamp electrophysiology

Coverslips with single hiAMs or hiAM monolayers were transferred to a patch clamp bath situated on the sample stage of an inverted microscope (Zeiss Axiovert 35). The bath was continuously superfused with the external solution containing (in mM): 140 NaCl, 5.4 KCl, 1.8 $CaCl_2$, 1.0 $MgCl_2$, 5.5 glucose and 5.0 HEPES-NaOH (pH 7.4). Custom-fabricated borosilicate glass pipette electrodes were back-filled with (in mM): 125 K-gluconate, 20 KCl, 5.0 NaCl, 0.22 amphotericin B (Sigma-Aldrich) and 10 HEPES-KOH (pH 7.2) and had a tip resistance in the range of 2–4 MΩ. The calculated liquid junction potential (−15.5 mV) was left uncorrected. Bath temperature was kept constant at 25°C by means of an ITO glass bath bottom (Cell MicroControls) and an inline solution heater (Multichannel Systems). Membrane potential recordings were obtained in the perforated whole cell configuration with series resistances <50 MΩ sampled at 10 kHz. In single hiAMs, APs were triggered by brief (2–10 ms) current pulses with suprathreshold amplitudes at 1 Hz. Monolayers were paced externally at a frequency of 1 Hz via custom-made bipolar electrodes placed close to the edge of the monolayer or by diffuse blue light (470 nm) illumination (0.5 mW/cm^2, 10-ms duration).

Light-induced whole cell Jaws currents were recorded in the ruptured patch configuration. Membrane potential was clamped at −60 mV and the series resistance was compensated electronically to >80%. Currents were sampled at 10 kHz after low-pass analog filtering using a cutoff value of 4 kHz. Capacitive currents were recorded in response to 10-mV hyperpolarizing voltage clamp steps from −40 mV. Cell size was estimated by calculating cell capacitance as the decay time constant of the capacitive transient over series resistance. Current amplitudes were normalized by the cell capacitance for each cell.

For the characterization of light responses in single cells and in monolayers, the cells were kept in the dark for 3 min to allow CheRiff and Jaws to relax to their fully dark-adapted states, after which the cells were illuminated sequentially by 1-s light pulses of increasing intensity with in-between dark periods of 30 s.

Instrumentation

Schematics of the experimental system is given in Figure S1A. V_m recordings were obtained by standard patch clamp electrophysiology using the Multiclamp 700B amplifier controlled by the pClamp software package (version 10.7.0.3) via the Digidata 1440A D/A interface (all from Molecular Devices). V_m was also sampled by a real-time computer hosting the Real-Time eXperiment Interface (RTXI)[50] via the PCI-6221 D/A interface (National Instruments). Real-time computation was carried out by custom scripts written for the RTXI platform hosted on a Linux computer (Ubuntu 16.04 LTS, 32GB RAM, Intel Core i7-4790 CPU). The static analog output generated by pClamp and the dynamic output from the RTXI were used as command signal for the patch clamp amplifier or for the modulation of LED power sources (LEDD1B, Thorlabs) separately or in combination added together by a custom voltage summing amplifier.

LEDs with emission centered at 470 (M470L3-C4, Thorlabs) and 617 nm (M617L3, Thorlabs) mounted on collimation lenses (COP1-A, Thorlabs) served as light source. Single cells were illuminated via a 40× magnification objective, whereas monolayers were illuminated directly after positioning the LEDs above the patch clamp bath.

Closed-loop control

Closed-loop control was realized with custom scripts written in C++ language to be installed as plugin for the RTXI platform.[50] All scripts are publicly available via https://doi.org/10.5281/zenodo.10116142.

For V_m control by direct current injection (Figure 2) and by single-actuator optogenetics (Figure 3), the APqr closed-loop control algorithm was used, the functionalities of which are described below (see also Figure S1B). At the beginning of each experiment, a number of consecutive APs are recorded under normal conditions and stored as V_m setpoint values in the array AP_{ideal}, according to Equation 1:

$$AP_{ideal}[i] = \frac{1}{lognum} \cdot \sum_{n=0}^{lognum} V_m^n[i] \qquad \text{(Equation 1)}$$

where *lognum* is the total number of normal APs logged chosen by the experimenter and *i* denotes discrete time. During the correction phase, the difference between the online measured V_m and the setpoint value ($AP_{ideal}[i]$) is calculated as V_m error (e) according to Equation 2:

$$e = V_m - AP_{ideal}[i] \qquad \text{(Equation 2)}$$

An output is then calculated according to Equations 3 and 4:

$$K_P = C_m \cdot \frac{1}{R_m} \qquad \text{(Equation 3)}$$

$$I_{out} = K_P \cdot e \qquad \text{(Equation 4)}$$

where C_m is cell capacitance and R_m is membrane resistance. I_{out} is used as command signal for the patch clamp amplifier or as modulatory signal for the LED driver. R_m is updated during each loop iteration depending on the conditions specified in Equation 5:

$$R_m = \begin{cases} R_{m\_corr\_up} \cdot R_m \text{ if } V_m \text{ overshoots } AP_{ideal}[i] \\ \dfrac{R_m}{R_{m\_corr\_down}} \text{ if } V_m \text{ moves away from } AP_{ideal}[i] \end{cases} \qquad \text{(Equation 5)}$$

where $R_{m\_corr\_up}$ and $R_{m\_corr\_down}$ are constants chosen by the experimenter.

For AP morphology restoration by dual-actuator optogenetics (Figures 4 and 5), the closed-loop control algorithm APqrPID was used (Figure S1C). APqrPID takes into account the past (the 'I' or integral term) and the future (the 'D' or derivative term), in addition to the momentary value of V_m error ('P' or proportion term). Similar to APqr, APqrPID records a chosen number of APs obtained under normal conditions and stores an average AP in AP_{ideal} as setpoint values according to Equation 1. During correction, V_m error (*e*) is calculated as specified in Equation 2. The I term, which speeds up or slows down the rate of change of the output levels based on the history of the V_m error is computed in every iteration as follows:

$$e_{sum} = e_{sum} + e \qquad \text{(Equation 8)}$$

The D term observes and predicts the trajectory of V_m by linear regression according to Equation 9:

$$e_{derivative} = \frac{l \cdot \sum_{j=0}^{l} e[-j] \cdot j \cdot \Delta t - \sum_{j=0}^{l} e[-j] \cdot \sum_{j=0}^{l} j \cdot \Delta t}{l \cdot \sum_{j=0}^{l} (j \cdot \Delta t)^2 - \left(\sum_{j=0}^{l} j \cdot \Delta t \right)^2} \tag{Equation 9}$$

where l denotes the amount of adjacent data points taken into account for linear regression (selectable, set to 10 by default). And finally, the output is computed according to Equation 10.

$$PID = K_P \cdot e + K_I \cdot e_{sum} + K_D \cdot e_{derivative} \tag{Equation 10}$$

where K_P, K_I and K_D are gain parameters. When PID takes a negative sign, its absolute value is used for the blue light source (depolarizing effect) and a PID with positive sign is used for the red light source (hyperpolarizing effect) as LED driver modulatory signal.

APqrLE was implemented for the purpose of AP waveform enforcement and is identical to APqrPID, except that in APqrLE, V_m setpoint values (i.e., the model APs) are stored on the computer and used for the calculation of V_m error. In addition, blue light output (0.5 mW/cm^2, 10-ms duration) is generated at 1 Hz to initiate APs by means of optogenetic pacing. When an AP upstroke is detected, correction is applied as specified for APqrPID.

QUANTIFICATION AND STATISTICAL ANALYSIS

All measured variables, including AP properties and V_m error were determined by analyzing raw data using custom-written Python scripts uniformly applied to all experiments, thereby eliminating observer bias. Data are represented as mean ± standard deviation (SD), unless otherwise specified. Normal distribution of data was verified by the Shapiro-Wilk test, after which group means were statistically compared by two-way analysis of variance assuming sphericity, followed by Tukey‘s *post-hoc* test for multiple comparisons by using GraphPad Prism 9 (GraphPad Software, LLC).

Cell Reports Methods, Volume *3*

Supplemental information

Opto-electronic feedback control of membrane potential for real-time control of action potentials

Balázs Ördög, Tim De Coster, Sven O. Dekker, Cindy I. Bart, Juan Zhang, Gerard J.J. Boink, Wilhelmina H. Bax, Shanliang Deng, Bram L. den Ouden, Antoine A.F. de Vries, and Daniël A. Pijnappels

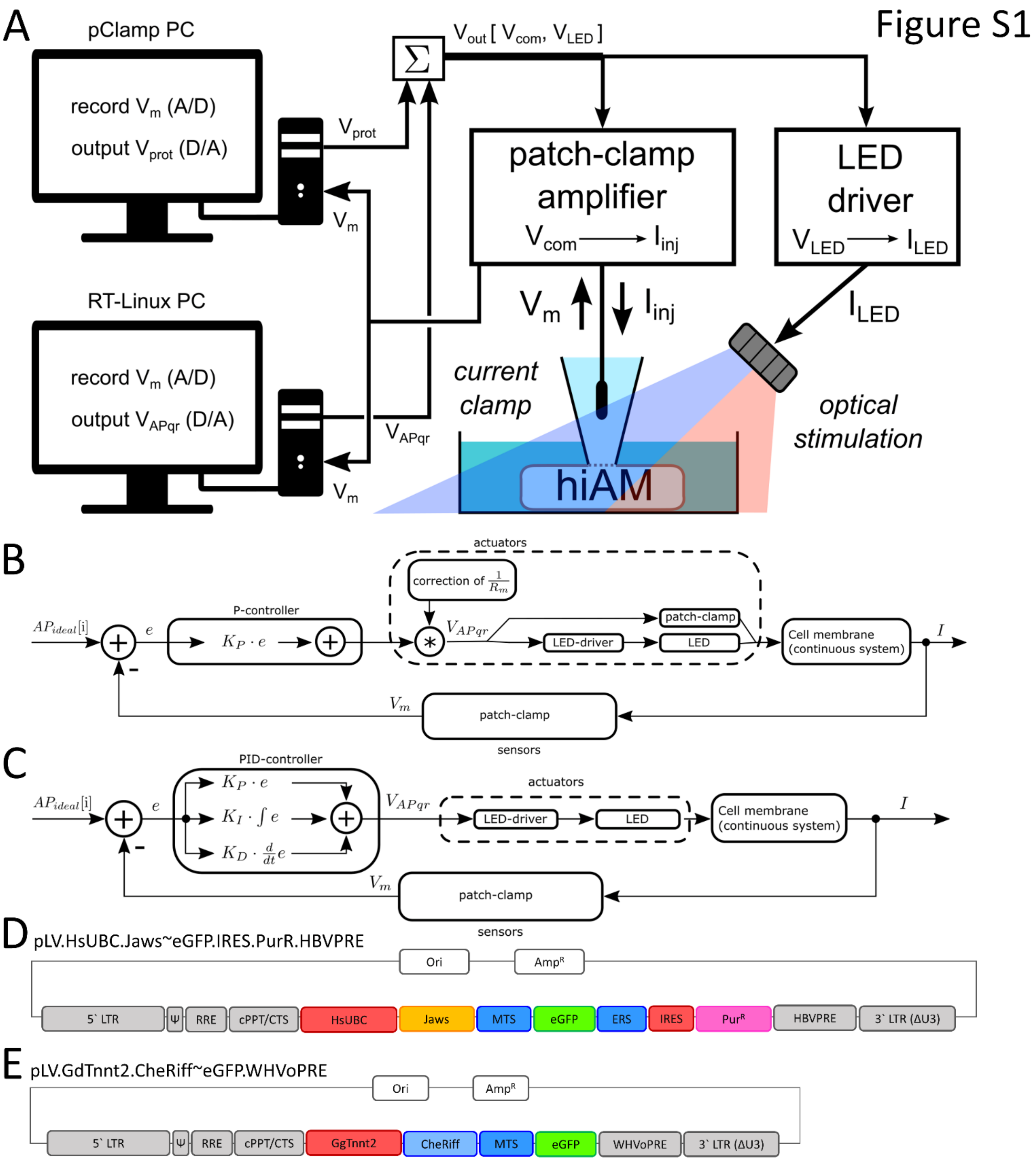

Figure S1 Schematics of the experimental setup, closed-loop controller algorithm and lentiviral vectors. Related to Figure 1 and STAR Methods. **(A)** Schematics of the experimental system and design. Real-time V_m data is obtained by the whole-cell mode of the patch clamp technique in single hiAMs or hiAM monolayers and is fed to two separate computers via analog-digital interfaces. A computer running the pClamp software package (pClamp PC) is used to record V_m and to create the preprogrammed analog output V_{prot}. The real-time computer (RT-Linux PC) also samples V_m and produces the dynamic analog output signal V_{APqr}. In our system, V_m can be targeted by direct current injection or by means of optogenetics. To generate electrical output, V_{prot} and V_{APqr} are summed by a custom-built summing amplifier (Σ) to yield V_{com} that serves as command input for the patch clamp amplifier to create I_{inj}. For optical output, V_{prot} and V_{APqr} may be summed or used separately in V_{LED} for the modulation of LED drivers generating I_{LED}. (**B**) Closed-loop controller used in single cell experiments. The output `I` was used either as command signal for the patch clamp amplifier to generate direct current injection or as control signal for the 617-nm LED driver. (**C**) Closed-loop controller used in dual-actuator optogenetic V_m control for AP restoration and for the enforcement of AP morphologies. **(D and E)** Schematic maps of lentiviral vector shuttle constructs used in the study for the over-expression of Jaws (D) and CheRiff (E). Indicated are viral vector elements (gray), including the human immunodeficiency virus type 1 5` long terminal repeat (LTR) containing the Rous sarcoma virus LTR promoter (5` LTR), the

U3 region-deleted 3` LTR (3` LTR [ΔU3]), packaging signal (Ψ), Rev-response element (RRE), central termination site and polypurine tract (cPPT/CTS) and the woodchuck hepatitis virus post-transcriptional regulatory element (WHVoPRE) or the human hepatitis B virus posttranscriptional regulatory element (HBVPRE), bacterial replication origin (Ori) and *Escherichia coli* β-lactamase gene (Amp^R). Jaws expression was driven by the human ubiquitin C promoter (HsUBC) and coupled with the expression of puromycin-N-acetyltransferase (PurR) via an internal ribosomal entry site (IRES). Both CheRiff and Jaws were C-terminally fused to the enhanced green fluorescent protein (eGFP). Jaws was equipped with the plasma membrane trafficking signal (MTS) and ER exit signal (ERS) of Kir2.1 [S1], whereas CheRiff was only equipped with the MTS of Kir2.1 [S2]. The expression of the CheRiff cassette was driven by the chicken troponin T promoter (GgTnnt2).

Figure S2

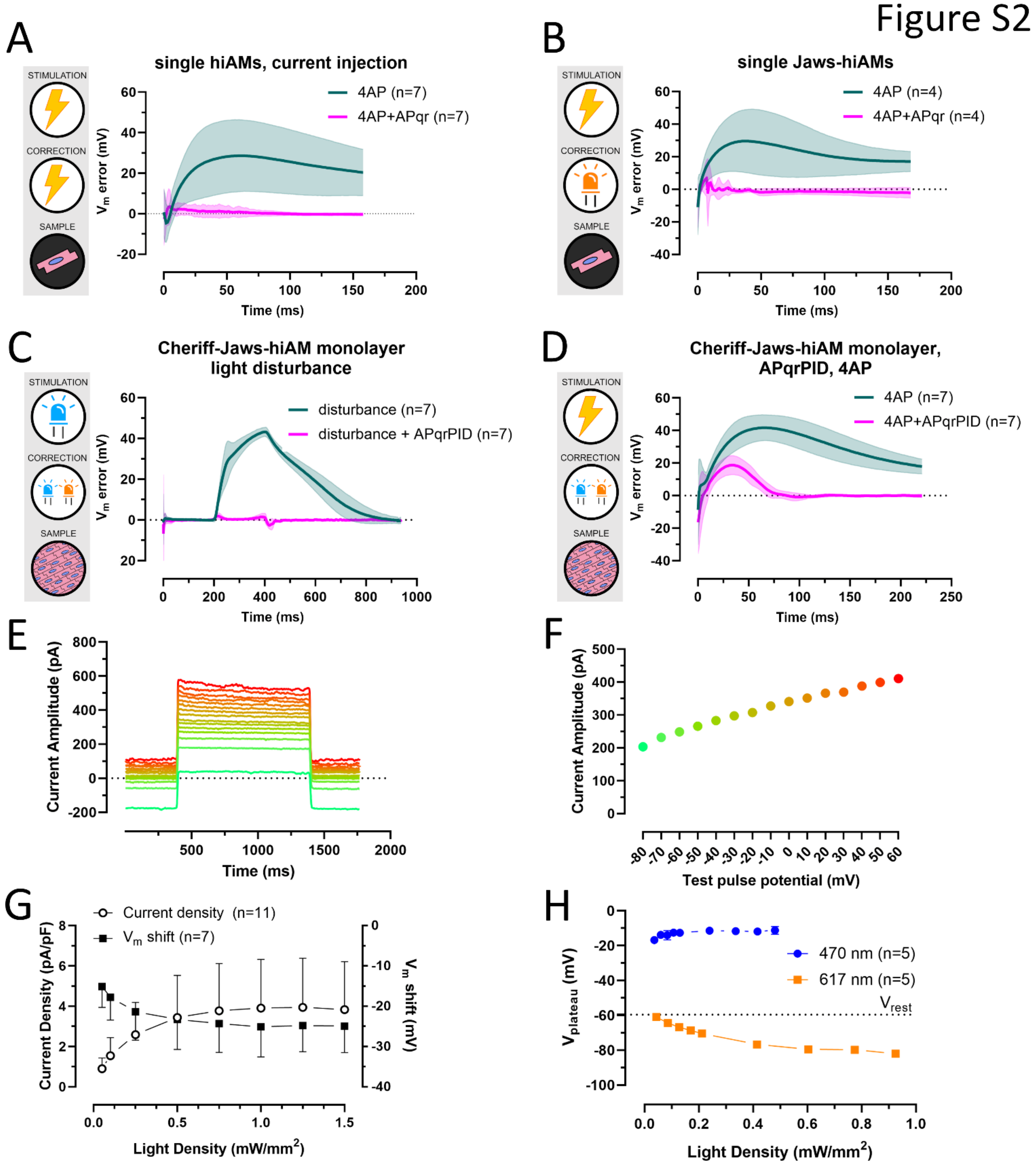

Figure S2 Extended data on V_m error during AP shape restoration and characterization of light response in optogenetically modified hiAM cells and monolayers. Related to Figures 2–7. (**A**) Average V_m error following 4AP administration (4AP) and during direct current injection-mediated AP restoration in the presence of 4AP (4AP+APqr) in n=7 single hiAMs. (**B**) Average V_m error following 4AP administration (4AP) and during light-mediated AP restoration in the presence of 4AP (4AP+APqr) in n=4 single hiAMs expressing Jaws. (**C**) Average V_m error in the presence of an optogenetically induced AP abnormality (disturbance) and during light-mediated AP restoration (dist.+APqrPID) in n=7 hiAM monolayers expressing CheRiff and Jaws. (**D**) Average V_m error following 4AP administration (4AP) and during light-mediated AP restoration (4AP+APqrPID) in n=7 hiAM monolayers expressing Cheriff and Jaws. In panels A to D, data are shown as mean ± SD, represented by continuous lines and shaded areas, respectively. A–D related to Figures 2–5. (**E**) Representative recording of Jaws current elicited by 565 nm light (1 s, 1.5 mW/mm^2) in a single hiAM from test potentials between -80 and 60 mV in 10 mV increments. (**F**) Current-voltage relationships obtained from the recording shown in panel E. (**G**) Whole cell current density (open circles, left axis) and V_m shift from V_{rest} (filled squares, right axis) observed at the end of 617-nm light pulses of 1 s and various intensities from 0.05 to 1.5 mW/mm^2 in single hiAMs expressing Jaws. E–G related to Figure 3. (**H**) $V_{plateau}$, representing V_m observed at the end of 470- and 617-nm light pulses of 1 s and various intensities from 0.04 to 0.48 mW/mm^2 and from 0.04 to 0.93 mW/mm^2, respectively, in hiAM monolayers expressing CheRiff and Jaws. Figure H related to Figures 4–7.

Figure S3

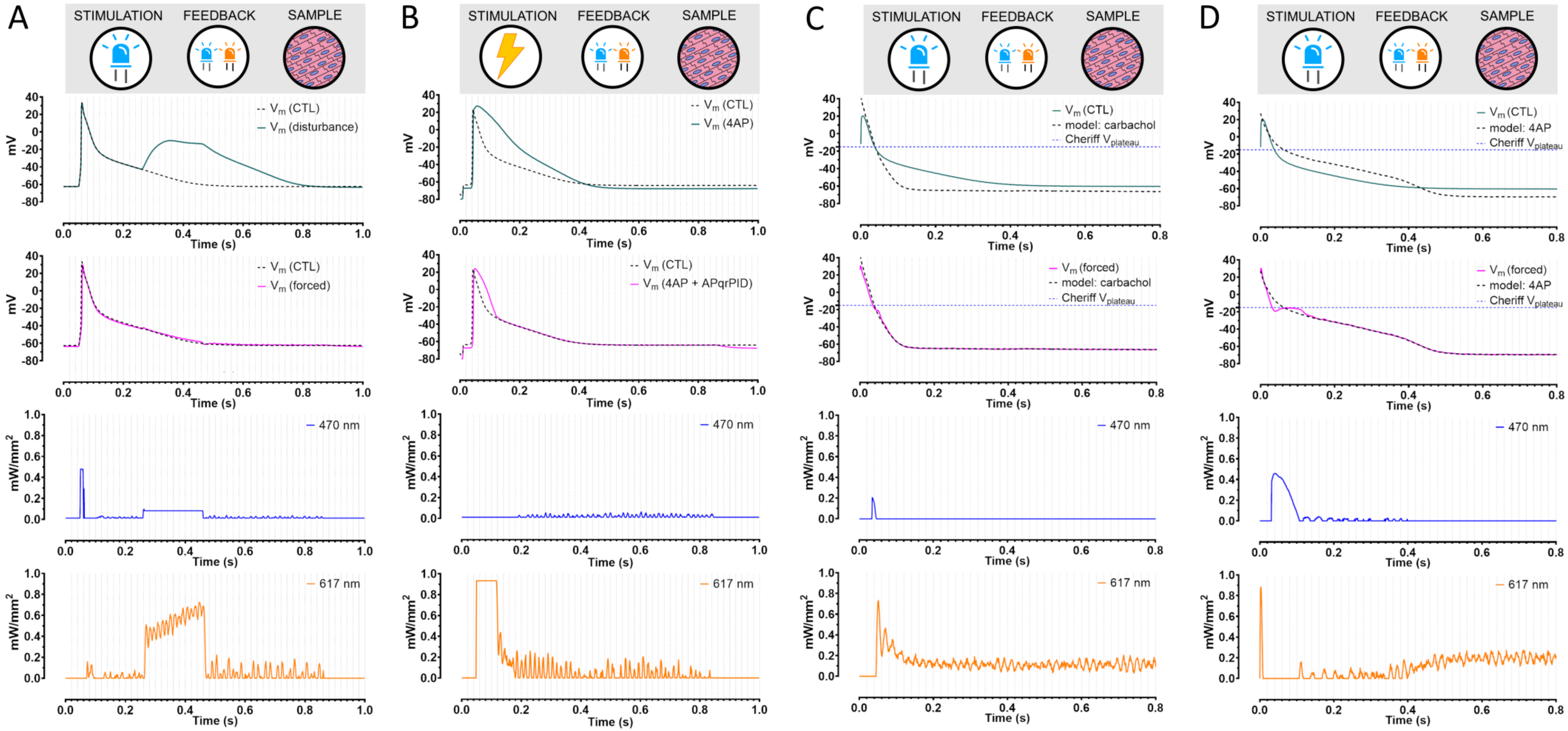

Figure S3 V_m and light output during AP shape restoration and enforcement experiments. Related to Figures 4, 5 and 6. (**A**) Representative recordings from the dual-actuator optogenetic AP restoration experiment in the context of a light-induced electrical disturbance (also shown in Figure 4C). (**B**) Representative recordings from the dual-actuator optogenetic AP restoration experiment in the context of a drug-induced electrical disturbance (also shown in Figure 5C). (**C**) Representative recordings from the dual-actuator optogenetic AP morphology enforcement of the 'carbachol' model APs (also shown in Figure 6B). (**D**) Representative recordings from the dual-actuator optogenetic AP morphology enforcement of the '4AP' model APs (also shown in Figure 6C).

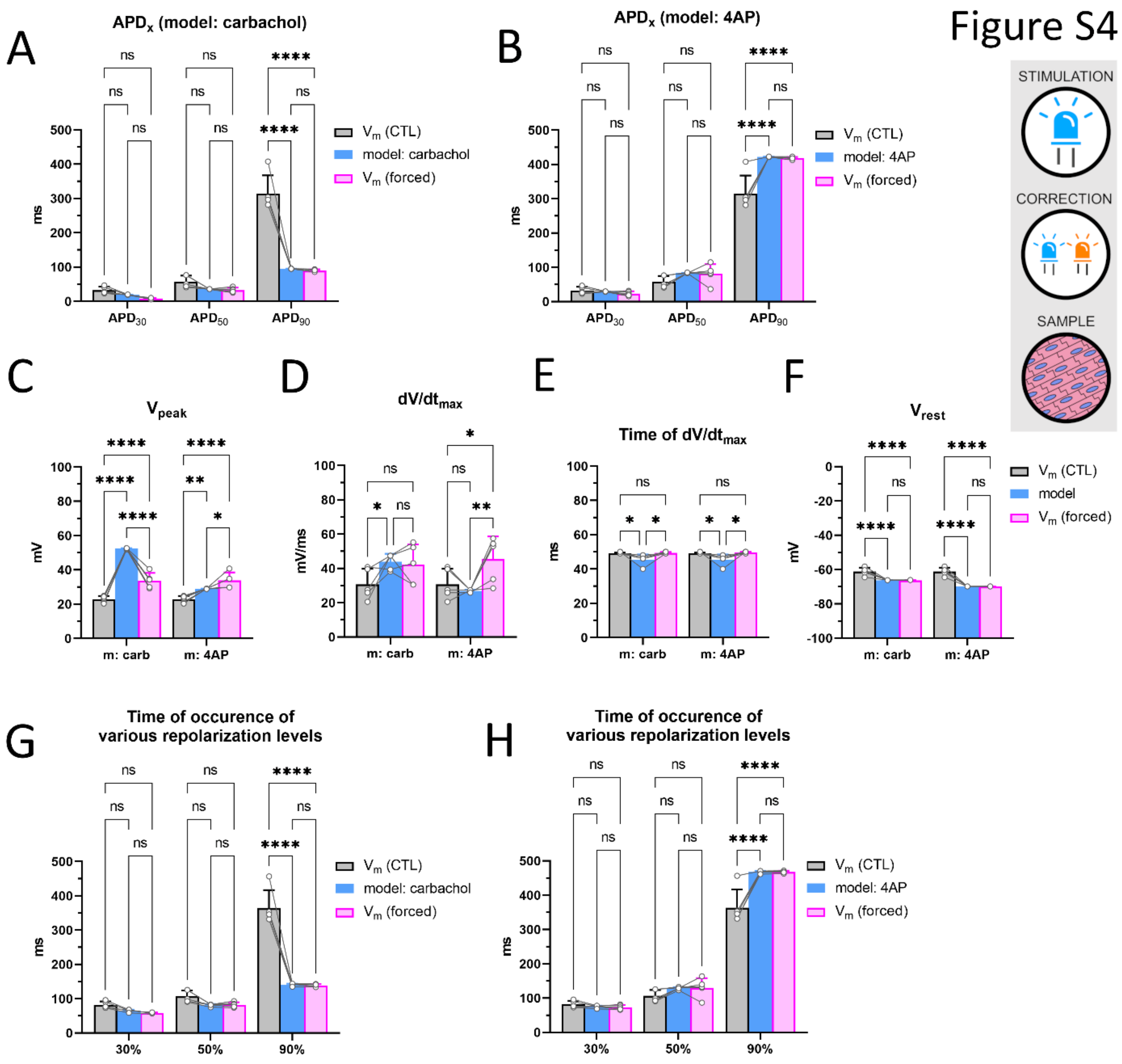

Figure S4 Extended data on AP parameters during enforcement of drug-induced AP abnormality models. Related to Figure 6. (**A and B**) Mean APD values ± SD measured at 20, 50 and 90% repolarization. (**C**) V_{peak} representing the most positive V_m value during an AP. (**D**) dV/dt_{max} representing maximum upstroke velocity. (**E**) Time to reach dV/dt_{max} from an arbitrarily chosen time point preceding the AP. (**F**) Resting potential. (**G** and **H**) The moment when 30%, 50% or 90% repolarization is reached during enforcement of the carbachol and the 4AP models. Data are shown as mean ± SD. *: $P < 0.05$, **: $P < 0.01$, ****: $P < 0.0001$, ns: not significant.

Figure S5

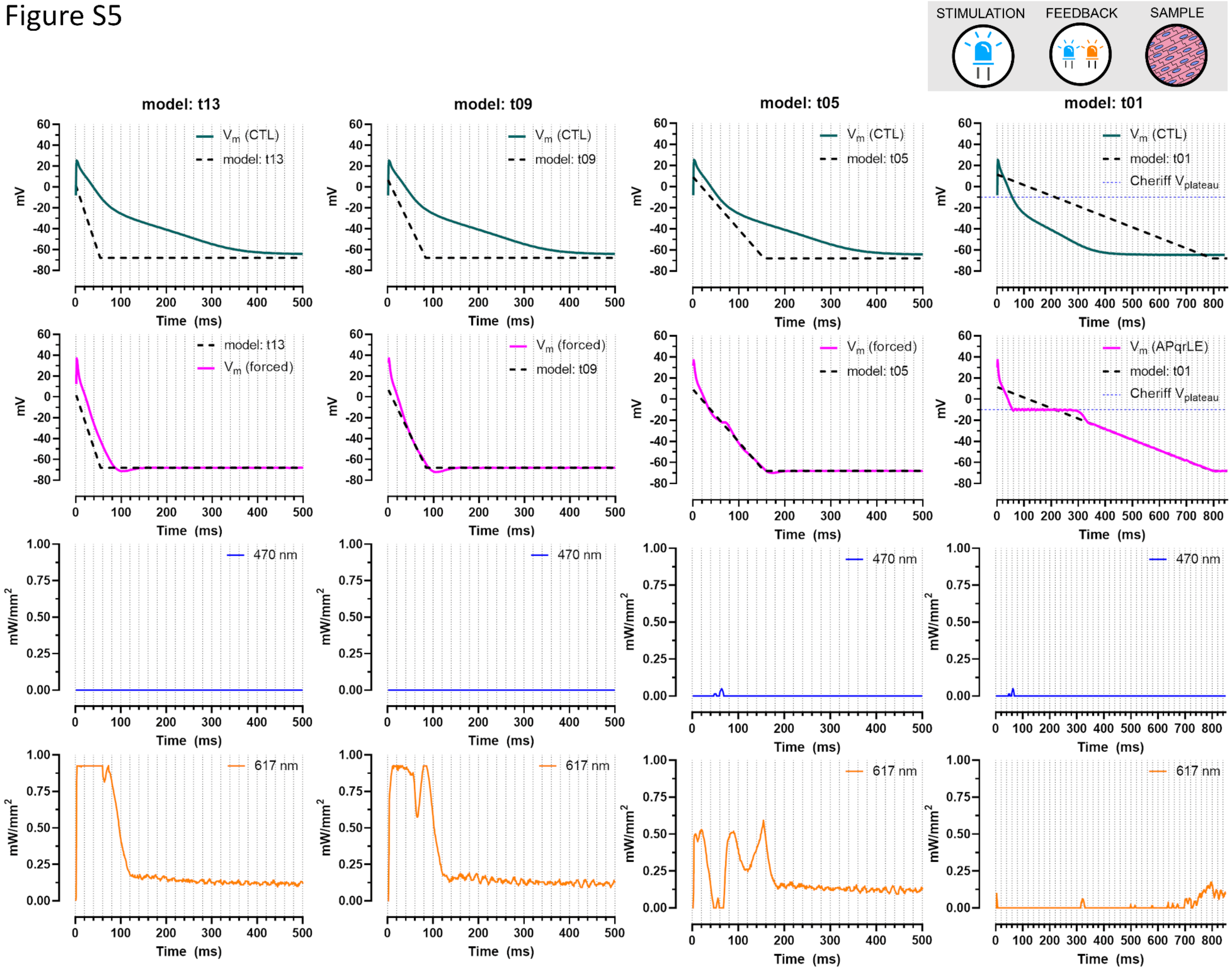

Figure S5 Representative recordings from the dual-actuator optogenetic AP morphology enforcement of the ‘t13’, ‘t09’, ‘t05’ and ‘t01’ model APs. Related to Figure 7.

Figure S6

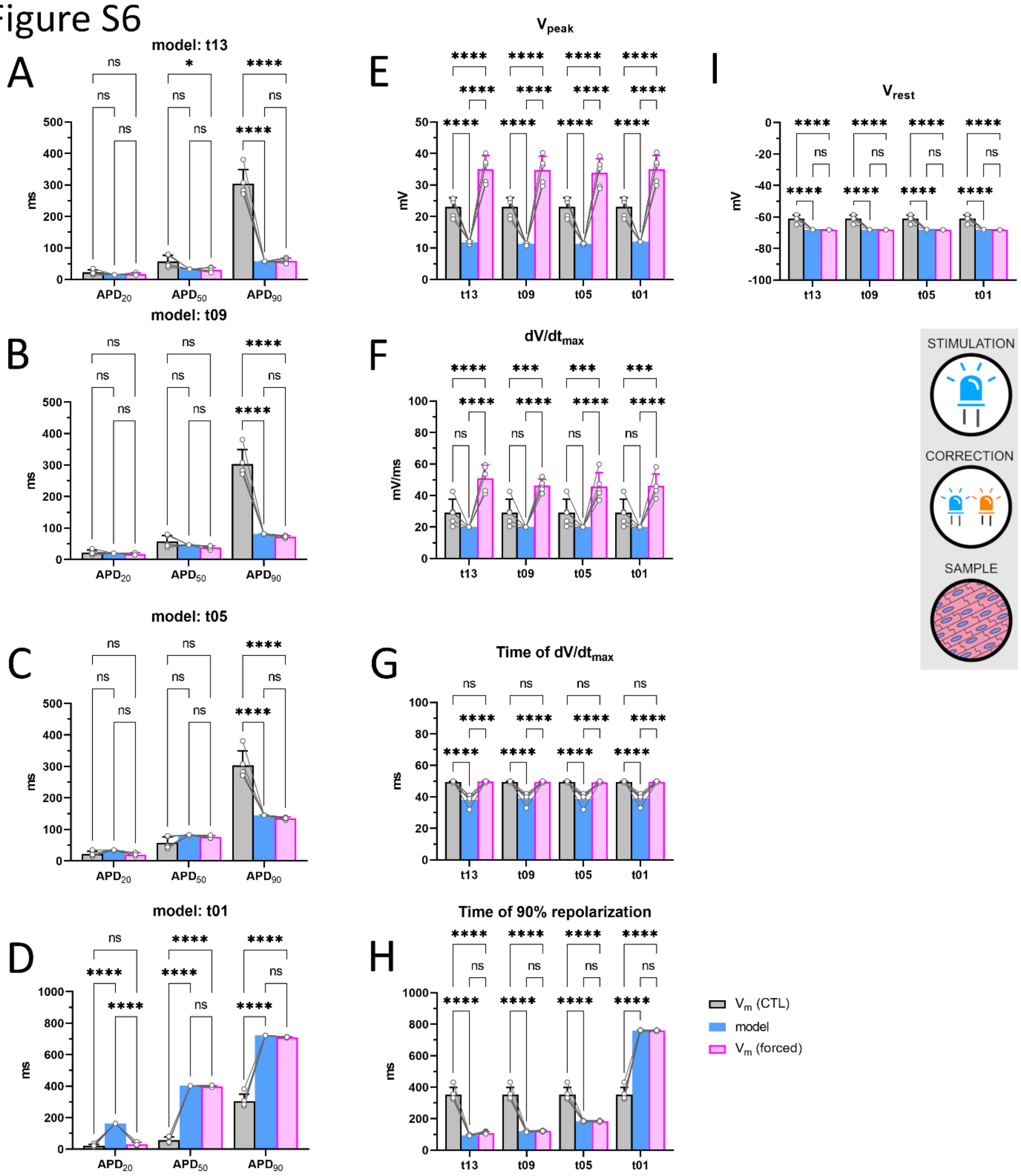

Figure S6 Extended data on enforcement of simplified AP models. The endogenous APs (CTL) were compared to model (model) and enforced (forced) APs. Related to Figure 7. (**A**–**D**) APD values measured at 20, 50 and 90% repolarization for the models t13, t09, t05 and t01, respectively. (**E**) V_{peak} representing the most positive V_m value during an AP. (**F**) dV/dt_{max} representing maximum upstroke velocity. (**G**) Time to reach dV/dt_{max} from an arbitrarily chosen time point preceding the AP. (**H**) The moment when 90% repolarization is reached. (**I**) Resting potential. Data are shown as mean ± SD. *: P < 0.05, ***: P < 0.001, ****: P < 0.0001, ns: not significant. Column labels for each panel are as shown in panel **H**.

Figure S7

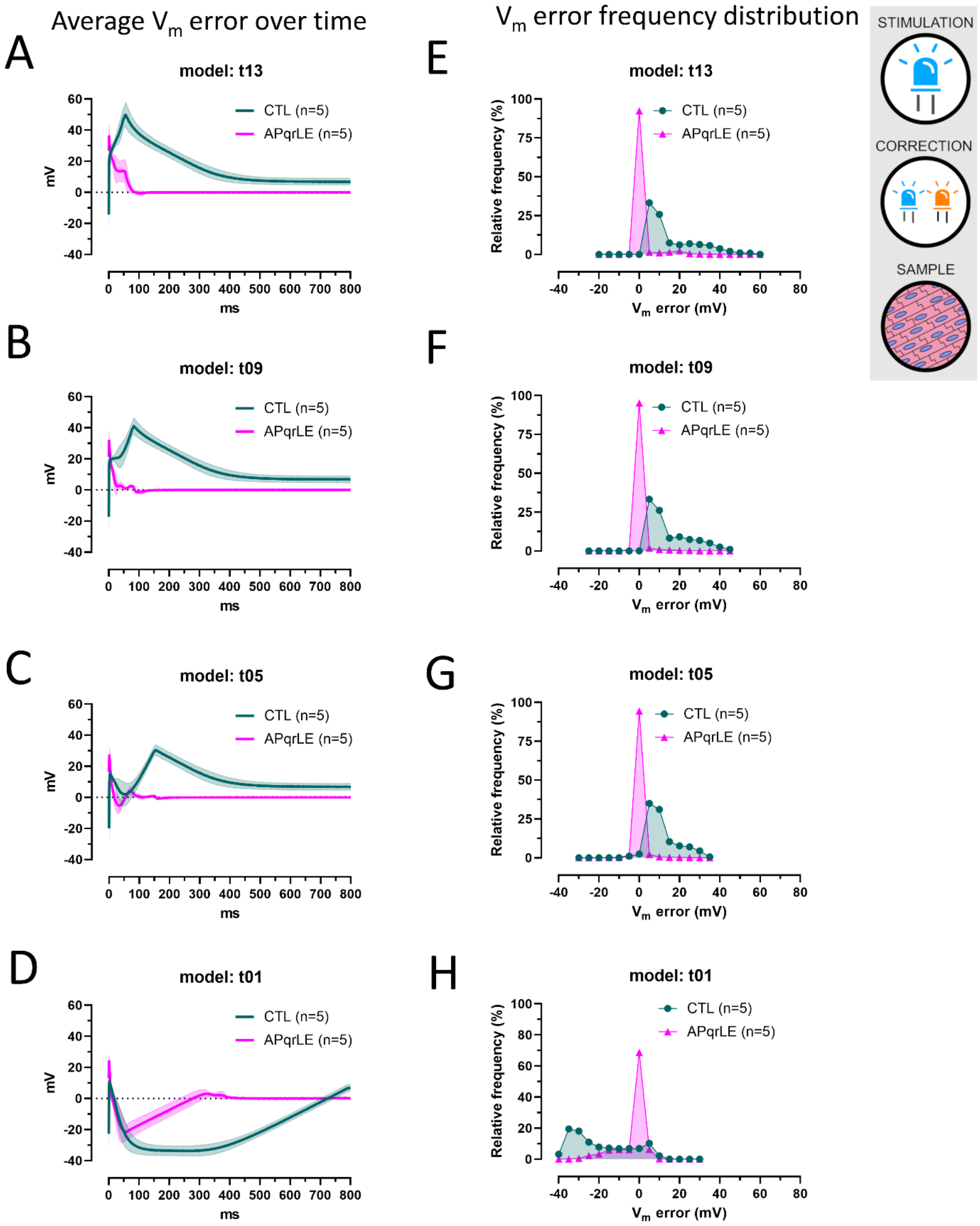

Figure S7 Extended data on enforcement of simplified AP models. Related to Figure 7. (**A**-**D**) V_m error representing the difference over time between model vs endogenous APs (CTL) and model vs enforced APs (APqrLE) for AP models t13, t09, t05 and t01, respectively. Data are shown as mean (continuous lines) ± SD (shaded areas). (**E**-**H**) Frequency distribution of V_m error for n=5 monolayers with a 5-mV bin width for AP models t13, t09, t05 and t01, respectively.

References

S1. Chuong, A.S., Miri, M.L., Busskamp, V., Matthews, G.A., Acker, L.C., Sorensen, A.T., Young, A., Klapoetke, N.C., Henninger, M.A., Kodandaramaiah, S.B., et al. (2014). Noninvasive optical inhibition with a red-shifted microbial rhodopsin. Nat Neurosci 17, 1123-1129. 10.1038/nn.3752.

S2. Hochbaum, D.R., Zhao, Y., Farhi, S.L., Klapoetke, N., Werley, C.A., Kapoor, V., Zou, P., Kralj, J.M., Maclaurin, D., Smedemark-Margulies, N., et al. (2014). All-optical electrophysiology in mammalian neurons using engineered microbial rhodopsins. Nat Methods 11, 825-833. 10.1038/nmeth.3000.
